# High-Resolution Human Kidney Molecular Histology by Imaging Mass Spectrometry of Lipids

**DOI:** 10.1021/acs.analchem.1c00649

**Published:** 2021-06-30

**Authors:** Lucía Martín-Saiz, Lorena Mosteiro, Jon D. Solano-Iturri, Yuri Rueda, Javier Martín-Allende, Igone Imaz, Iván Olano, Begoña Ochoa, Olatz Fresnedo, José A. Fernández, Gorka Larrinaga

**Affiliations:** † Department of Physical Chemistry, Faculty of Science and Technology, University of the Basque Country (UPV/EHU), Barrio Sarriena, s/n, Leioa 48940, Spain; ‡ Service of Anatomic Pathology, Cruces University Hospital, University of the Basque Country (UPV/EHU), Cruces (Barakaldo) 48903, Spain; § BioCruces Health Research Institute, Cruces (Barakaldo) 48903, Spain; ∥ Department of Physiology, Faculty of Medicine and Nursing, University of the Basque Country (UPV/EHU), Barrio Sarriena, s/n, Leioa 48940, Spain; ⊥ Service of Urology, Cruces University Hospital, Cruces (Barakaldo) 48903, Spain; # Department of Nursing I, Faculty of Medicine and Nursing, University of the Basque Country (UPV/EHU), Barrio Sarriena, s/n, Leioa 48940, Spain

## Abstract

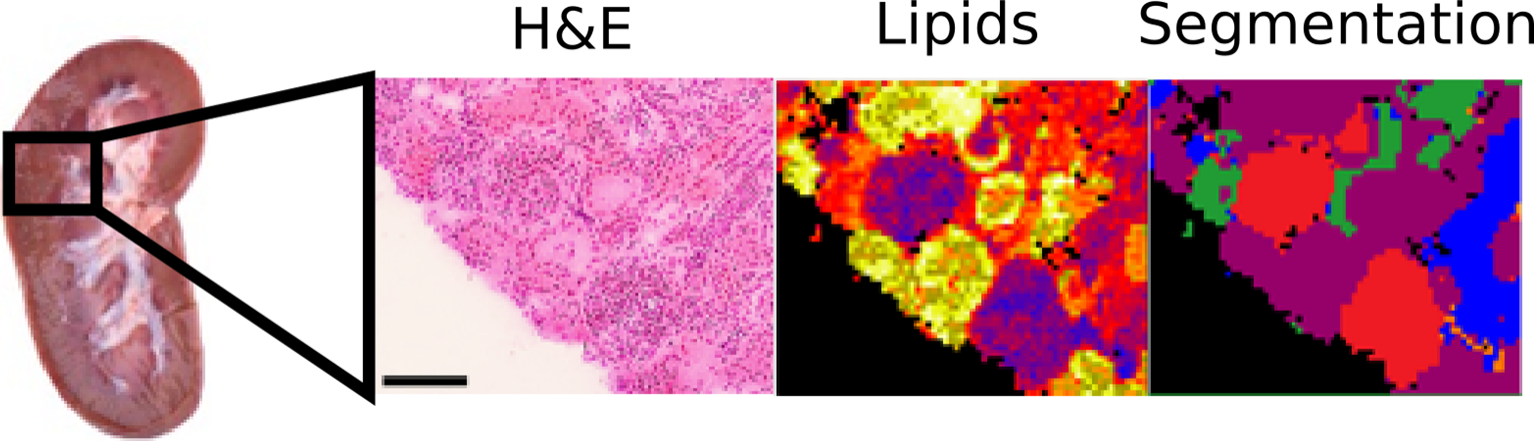

For many years, traditional histology has been the gold standard for the diagnosis of many diseases. However, alternative and powerful techniques have appeared in recent years that complement the information extracted from a tissue section. One of the most promising techniques is imaging mass spectrometry applied to lipidomics. Here, we demonstrate the capabilities of this technique to highlight the architectural features of the human kidney at a spatial resolution of 10 μm. Our data demonstrate that up to seven different segments of the nephron and the interstitial tissue can be readily identified in the sections according to their characteristic lipid fingerprints and that such fingerprints are maintained among different individuals (*n* = 32). These results set the foundation for further studies on the metabolic bases of the diseases affecting the human kidney.

## Introduction

For many years, traditional histology has been the gold standard for the diagnosis of many diseases. The importance of this technique is reflected in the large number of chemical and immunohistochemical (IHC) procedures developed, which enable highlighting multiple aspects of the architecture of a tissue.^1,2^ In recent years, new image techniques have appeared that add a different point of view and try to avoid the use of labels for the visualization of the tissues, precluding their alteration. Perhaps, the most impressive advances in histological images have come from the relatively new technique of matrix-assisted laser desorption/ionization-imaging mass spectrometry (MALDI-IMS) (or abbreviated, IMS).^3^ This technique enables visualization of proteins and metabolites in frozen tissue sections with minimal sample preparation, avoiding rapid metabolic changes, with spatial resolutions ranging from ∼100 to 1 μm/pixel.^4−6^ In this technique, the sample is freshly frozen (Figure S1). Then, sections of the sample are obtained using a cryomicrotome and are covered with a suitable matrix that enables the extraction of analytes using a laser. To explore samples using a mass spectrometer, a grid of coordinates is defined, which will become the pixels of the final images. Then, the spectrometer records a mass spectrum at each coordinate of the grid. Finally, the image of the distribution of all the species detected is reconstructed using a specialized software.

Probably, the main complication of the technique lies in hardware development and data interpretation. To fully develop IMS, it is necessary and important to make an effort to design new mass spectrometers that can record up to 50 pixels/s, yielding enough information to achieve precise identification of multiple species directly extracted from the tissue.^7,8^ The development of new and imaginative protocols for sample preparation is also necessary.^9,10^

Regarding data interpretation, there are still open questions: normalization, segmentation of the images, and identification of the species are still not fully resolved questions.^11,12^ Even with these pending tasks, IMS shows tremendous potential to characterize the proteomics and lipidomics of tissue sections. An increasing number of studies show, for the first time, the tissue architecture through the eyes of (mainly) lipids and proteins, unveiling features that were not previously described. For example, the exquisite regulation of the lipid phenotype during colonocyte maturation^13^ or the differential lipid expression of epidermis, dermis, and melanocytes in nevus cryosections.^14^ All these studies are the foundation and the next step in the understanding of the metabolic traits of the diseases.

The pathological diagnosis of kidney functions and disease is currently based on the examination of tissue biopsies by optic and electron microscopy and immunofluorescence (IF).^2^ However, the incorporation of innovative techniques based on transcriptomic characterization of isolated cells or microdissected tubule segments has facilitated the development of new approaches to classify renal structures and their specific diseases.^1,15^ In this context, MALDI-IMS emerges as a technique that enables the exploration of kidney molecular features and that has a great potential as a complement for imaging techniques in clinical routine.^16^

Here, we present a description of the architecture of the human kidney from the point of view of lipids. Several studies have already been reported, in which IMS has been used either to describe the lipidome of healthy rat^17,18^ and mouse^16,19−21^ kidney or in the context of a disease.^22^ However, none of them was carried out with enough spatial resolution to establish how large the differences in lipid expressions are between the different sections of a nephron. This is an important issue as each specific segment of the nephron is susceptible to different mechanisms of damage that leads to both nonneoplastic^23^ and neoplastic diseases.^24,25^ Thus, mapping the lipidome of these cells and understanding their differences is an important step forward toward shedding light on the metabolic origins of the diseases affecting the kidney.

## Materials and Methods

### Materials and Reagents

1,5-Diaminonaphthalene (DAN), hematoxylin, eosin, ethanol (99.99% purity), HCl, toluene (analytical standard), ammonium formiate (99.999%), and xylene for histological staining were purchased from Sigma-Aldrich (Steinheim, Germany). Water, methanol, 2-propanol, and formic acid (optima quality) were purchased from Fisher Scientific (Fair Lawn, NJ, USA).

Kidney sample collection: All the experiments carried out in this study comply with the current Spanish and European Union legal regulations. Samples and data from patients were provided by the Basque Biobank for Research-OEHUN (www.biobancovasco.org). All patients were informed about the potential use of their surgically resected tissues for research, and they manifested their consent by signing a specific document approved by the Ethical and Scientific Committees of the Basque Country Public Health System (PI+CES-BIOEF 2018–04).

Kidney samples were obtained prospectively from a series of nephrectomies from 32 renal cancer patients (18 males and 14 females, age: 65 ± 8 years) in the University Hospitals of Cruces (Barakaldo, Spain) and Basurto (Bilbao, Spain). The uninvolved part of the kidney was stored fresh frozen (−80 °C), and contiguous sections of 16 and 3 μm thicknesses were obtained for MALDI-IMS and preliminary histological analyses, respectively. The latter allowed us to identify the areas that contained the most valuable information about the composition of the nephrons, which are found in the renal cortex and medulla, and select them for subsequent MALDI-IMS exploration. One must take into account that the whole area of the sections could not be scanned because of the speed of the mass spectrometer.

After exploration by MALDI-IMS, the sections were stained with hematoxylin and eosin (HE), so the pathologists could annotate the histological areas and structures in order to correlate them with the segments obtained by MALDI-IMS. It is important to highlight that HE staining was performed on the same sections used for MALDI-IMS, and not in paraffin sections, because the complexity of the tissue makes it impossible to correlate the structures in two sequential sections; MALDI-IMS has to be carried out in fresh tissues. This procedure limited the degree of the details the pathologists could extract from the histological analysis of kidney structures. Such information was complemented with additional IF experiments (description of the IF protocols can be found in the Supporting Information).

### MALDI-IMS Experiments

Histological sections from 32 different patients were prepared and analyzed by MALDI-IMS, as described by Garate et al.^26^ Detailed description of the protocol can be found in the Supporting Information. Briefly, DAN was used as the matrix for negative-ion detection and was deposited with the aid of our in-house-designed sublimator.^9^ The sections were scanned in negative-ion mode using the orbitrap analyzer of a MALDI-LTQ-Orbitrap XL (Thermo Fisher, San Jose, CA, USA), equipped with a modified MALDI source.^27^

Data were acquired with a mass resolution of 60,000 at *m/z* = 400. Two microscans of 10 laser shots were recorded for each pixel using a 10 μm raster size. Spectra were processed using an in-house-developed software, built using Matlab (MathWorks, Natick, USA). Lipid assignment was achieved using the m/z value, the “on-tissue” MS/MS and MS^3^ data, and the ultrahigh performance liquid chromatography/electrospray ionization-mass spectrometry (UHPLC/ESI-MS/MS) results (see the Supporting Information). With this procedure, it was not possible to distinguish between ether and vinyl-ether lipids.^28^

Regarding lipid abundance, the MALDI-IMS protocol used in this work only gives relative abundance within each lipid class. This means that signal intensity cannot be translated directly into lipid abundance. Therefore, one must limit the discussion to analyze relative variations in the abundance of the species of a given family.

Data from each section were analyzed using a segmentation algorithm (HD-RCA) to isolate and identify the lipid signatures of each histological area in the section^29−31^ (see additional methods in the Supporting Information). To establish the number of segments on each image, a heuristic approach was used: the initial number of segments was set to 5 for cortical and medullary samples and to 8 for cortical-medullary transition sections. Then, the segments suggested by the algorithm were verified by examining their correlation: those segments whose correlation was higher than 95% were grouped together because such a high correlation seems to indicate that they define similar histological areas. In the end, the total number of segments found in all the samples was 131, which were divided into eight histological areas. The signatures from the final histological areas were later used in subsequent multiexperiment analysis.

The evaluation of the statistical significance of the differences in lipid fingerprints among the eight identified histological areas, Levene test, analysis of variance (ANOVA) univariate statistical analysis, and Tukey/Games-Howell post hoc were computed using SPSS Statistics 17.0 (IBM, Armonk, NY, USA).^32^ The Levene test determines the homogeneity (*H*_0_ = groups have equivalent variance) to choose the post hoc method: Tukey, if Levene *p* ≥ 0.05 and Games-Howell, if Levene *p* ≤ 0.05. Principal component analysis (PCA) and classification models were carried out using Orange Biolab 2.7.8 (Ljubljana, Slovenia).^33^

## Results

### Identification of Kidney Histological Structures by IMS of Lipids

**Figure 1:**
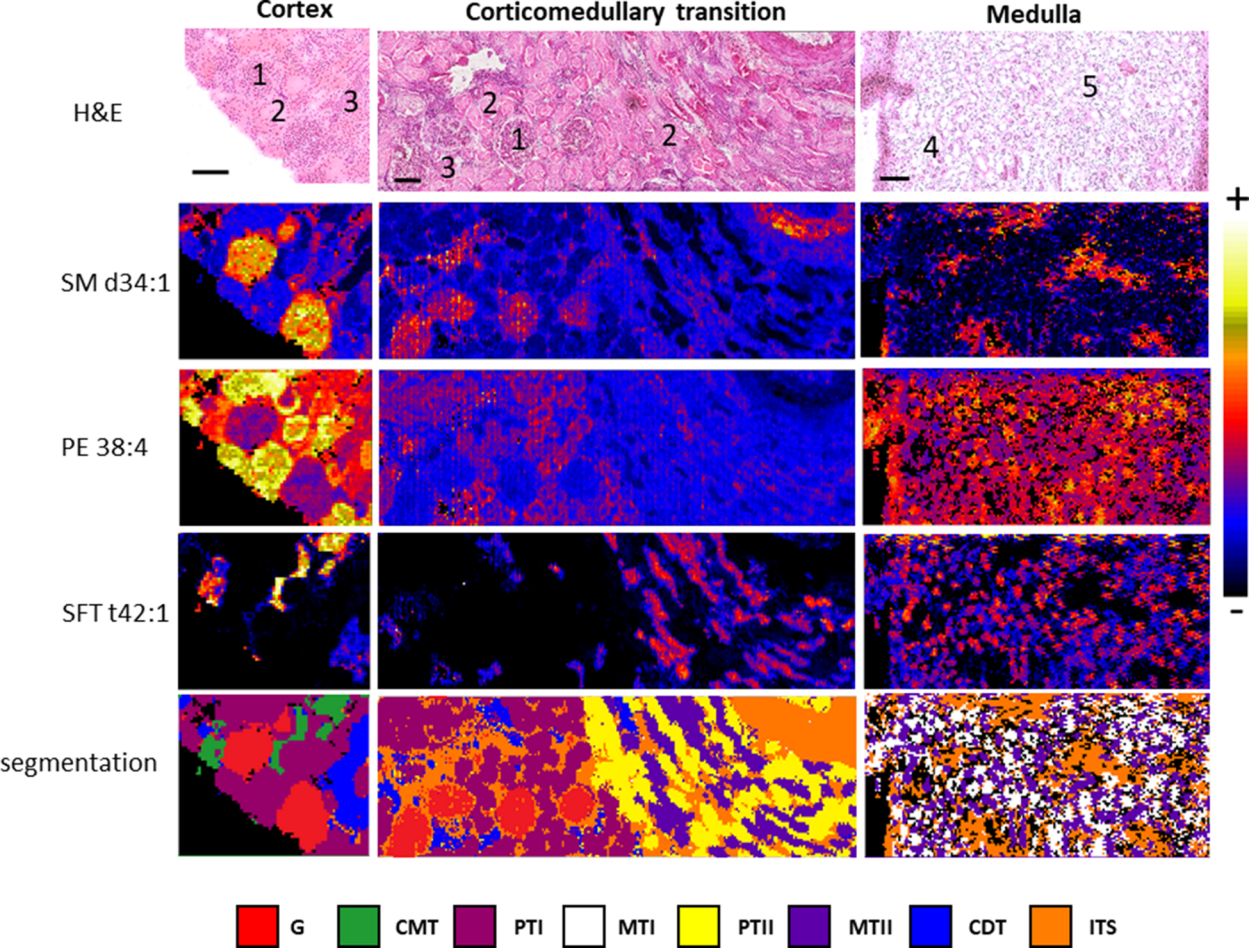
Distribution of three representative lipids over three example sections of the human kidney cortex, corticomedullary transition, and medulla, together with the segmentation analysis. The number on the HE-stained optical images represents five different histological structures: (1) glomeruli, (2) proximal tubules, (3) distal tubules, (4) medullary tubules, and (5) interstitium. Abbreviations: HE, hematoxylin and eosin; G: glomeruli; CMT: corticomedullary tubules; PTI: proximal tubules I; MTI: medullary tubules I; PTII: proximal tubules II; MTII: medullary tubules II; ITS: interstitial vascular structures. Images were recorded in negative-ion mode at a pixel size of 10 μm. Scale bar = 150 μm.

Figure 1 shows the optical image of three HE-stained sections of the cortex, corticomedullary transition, and medulla of the human kidney. Glomeruli, proximal and distal tubules, collecting ducts, and interstitial vessels were identified by optical microscopy. The same sections were scanned in IMS experiments using a pixel size of 10 μm in negative-ion mode. The intensity of 130 lipid species was directly captured from the fresh tissue sections, obtaining a distribution map for each of the species. Example images for the distribution of sphingomyelin (SM) d34:1, phosphatidylethanolamine (PE) 38:4, and sulfatide (SFT) t42:1 are also shown in Figure 1. These lipids are not uniformly distributed. SM d34:1 is preferentially found in glomeruli, but it is also found in the interstitial vascular structures surrounding glomeruli and tubules from both the cortex and medulla of the kidney. On the other hand, PE 38:4 shows higher abundance in proximal tubules, while the SFT t42:1 relative concentration is higher in medullary tubules.

The differential lipid fingerprint at each pixel was further analyzed to define areas with similar lipid profiles. For this purpose, a segmentation algorithm was applied to group the pixels according to the similarity of their lipid fingerprints, defining the segments of pixels with a common lipid fingerprint. An example of the result from this analysis can be found in Figure 1, while the images for all the samples analyzed in this work (*n* = 32) are presented in the Supporting Information (Figures S2–S4).

**Figure 2:**
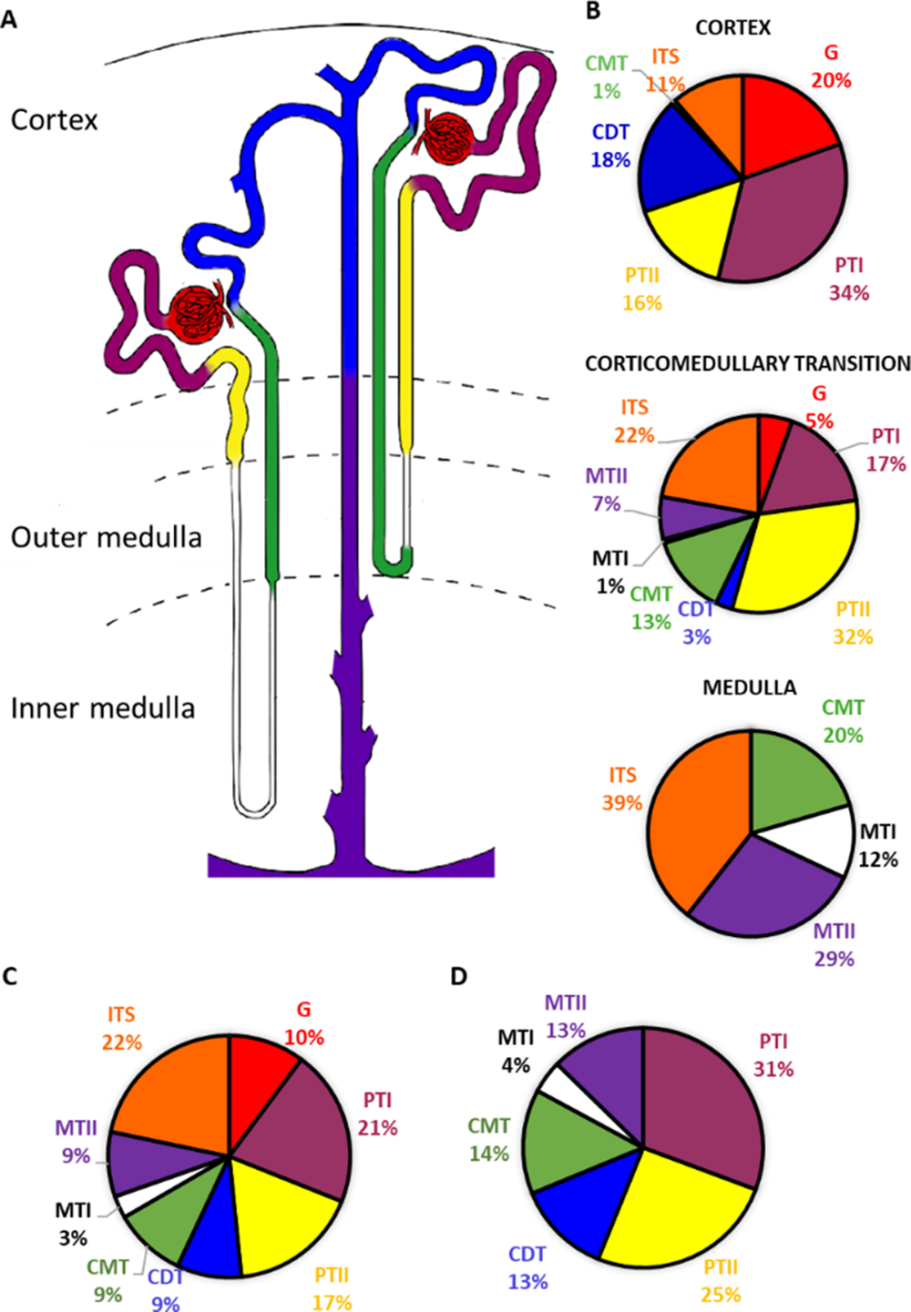
Renal tissue characterization by MALDI-IMS of lipids. (A) Sketch of a nephron, with the different segments colored following the code shown in Figure 1. The scheme shows a hypothetical distribution of the seven renal areas with the characteristic lipid profile detected by IMS. The eighth structure corresponds to ITS; therefore, it does not appear in this diagram; (B) Ratio of the total area covered by each histological structure in the cortex, corticomedullary, and medullary samples, including the vascular structures (G and ITS); (C) Similar analysis, but performed over all the samples, including vascular structures; (D) Same analysis as in (C), but excluding vascular structures. The analysis in (B)–(D) was performed with the final number of segments (131) obtained from the IMS images. Drawing follows the original Renal Commission of the International Union of Physiologic Sciences publication^38,42^ and was adopted from ref (38). with permission from John Wiley and Sons.

Up to eight different fingerprints (segments) were found in the sections measured, using the segmentation algorithm. The analysis of their localization in the sample and comparison with the observations from the pathologists enabled their assignment to (Figures 1 and 2) glomeruli (G), proximal tubules (PTI and PTII), cortical distal tubules (CDTs), corticomedullary tubules (CMTs), medullary tubules (MTI and MTII), and interstitial structures (ITS). All these structures are distributed among cortical, corticomedullary transition, and medullary samples. A summary of all the information from the IMS images is shown in Figure 2A in the form of a colored nephron.

We also calculated the percentage that each structure with a specific lipid fingerprint represents in the total structures detected by IMS in all the samples (Figure 2B and C and Figure S5). Thus, lipid fingerprints corresponding to proximal tubules (PTI and PTII) represent 56% of the total epithelial structures detected. CMTs represent 14%, CDTs 13%, MTIIs 13%, and MTIs 4%. If we take into account epithelial and vascular structures, these ratios vary slightly, and both G and ITS account for a similar percentage, 9% of the total structures detected with IMS (Figure 2C and D).

A closer look at the IMS results reveals the existence of additional information in the images. Certainly, the large differences between glomeruli and tubules hide other subtle differences. When each of the segments found were isolated and reanalyzed, the results shown in Figure S6 were obtained. The images show the existence of further differences in the lipid profile inside each histologic area that may correspond to different cell subpopulations and cross-sectional heterogeneity. Such diversity is already observed in the distribution of the example lipids shown in Figure S7 for one cortex and one corticomedullary transition section. For example, a thorough analysis of the renal corpuscles shows a new structure surrounding the glomeruli that may correspond to the Bowman’s capsule (Figure S8). Although this structure has a lipid profile close to that of glomeruli, IMS experiments can distinguish between both structures in several samples when the segments are reanalyzed and subdivided.

### Statistical Analysis of the Differences Observed between Histological Areas

**Figure 3:**
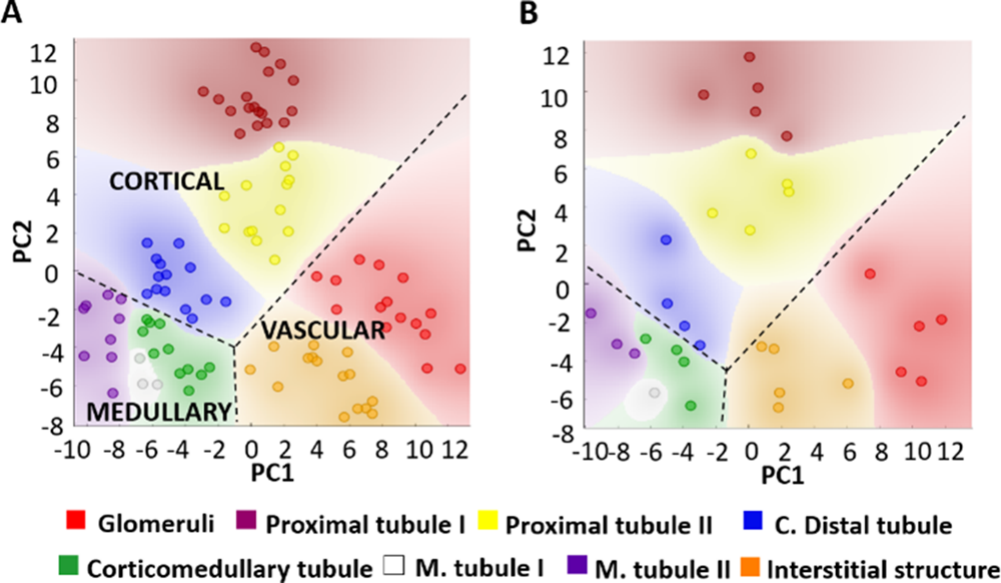
PCA of the segments obtained from the IMS images. The segments were randomly divided into discovery (*n* = 99) and validation (*n* = 32) groups. (A) Using all the species identified in the 32 tissue sections, a perfect separation among the eight IMS histological structures was achieved in the discovery group. An additional division can be established between the cortical, medullary, and vascular clusters; (B) PCA of the segments in the validation group using the significant lipids deduced from the discovery group. The full list of lipid species can be found in Supplementary Table S1.

To test if the differences in lipid fingerprints between the histological areas defined by IMS are statistically significant, the segments obtained from the renal samples were randomly divided into a discovery group and a validation group. Then, PCA was carried out on the first group, obtaining the neat separation shown in Figure 3A. The lipid species that experienced a significant variation between tissue areas (Table S1 and Figures S9–S19) were then used to analyze the validation group, resulting in a clear separation between the areas (Figure 3B). The confusion matrix and the performance of each statistical model tested are shown in Figure S20. The method that achieved the best results was random forest, with an AUC (area under the curve) of 0.987, a precision of 0.885, and a recall of 0.844. All these values indicate that the lipid fingerprints of the histological areas found are representative of each area and invariant among individuals. Furthermore, additional information can be extracted from Figure 3A: the proximity among the colored areas in the PCA means a higher lipid correlation among the histological structures found in the IMS segmentation images. Consequently, the lipid expression of predominant cortical tubules PTI, PTII, and CDT is more similar and differs from that of the prevalent medullar tubules CMT, MTI, and MTII. Vascular structures, including G and ITS, present closer profiles of the lipid expression.

### Analysis of the Differences in Lipid Composition between the Histological Areas

**Figure 4:**
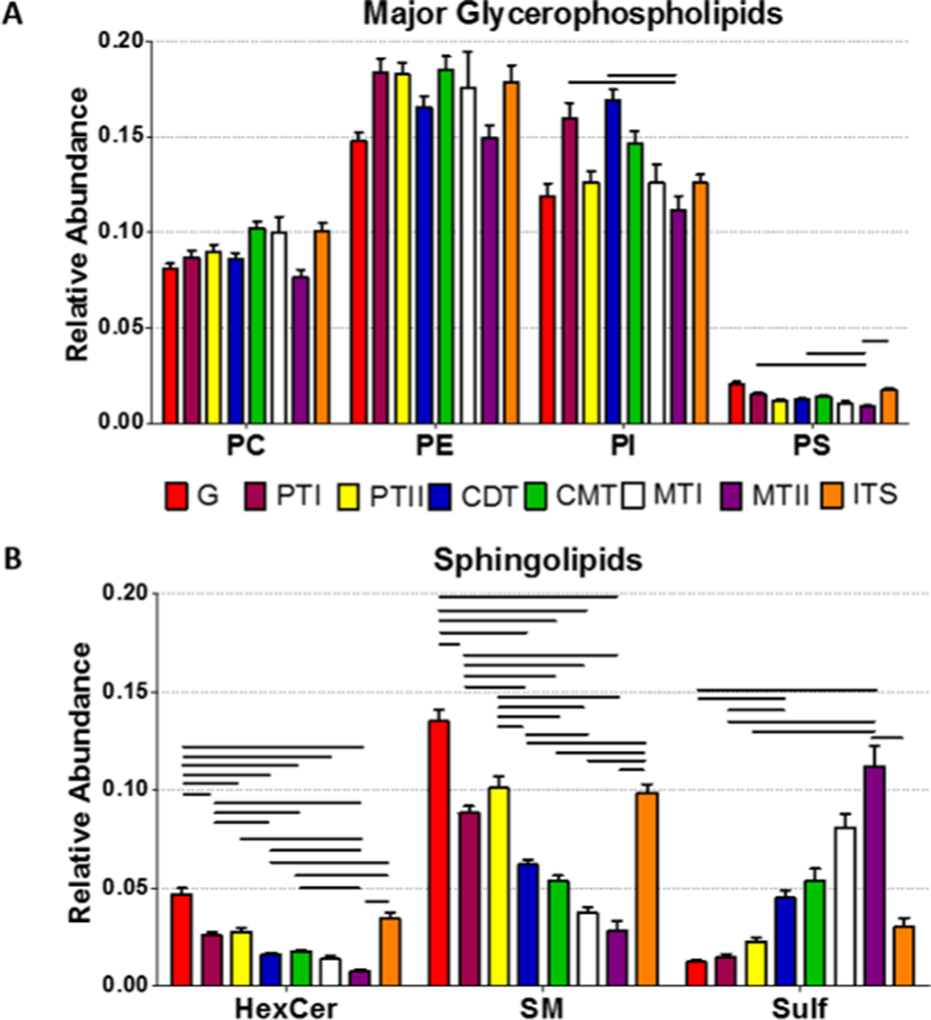
Relative abundance of main glycerophospholipids (A) and sphingolipid (B) families in the eight histological structures identified in the IMS images. Abbreviations: PC, phosphatidylcholine; PE, phosphatidylethanolamine; PI, phosphatidylinositol; PS, phosphatidylserine; HexCer, hexosylceramides; SM, sphingomyelin; SFT, sulfatide; G, glomeruli; PTI, proximal tubule I; PTII, proximal tubule II; CDT, cortical distal tubule; CMT, corticomedullary tubule; MTI, medullary distal tubule I; MTII, medullary distal tubule II; ITS, interstitial structures. Values are expressed as mean ± SEM. The statistical significance bars were assessed using post-hoc analysis with a *p*-value ≤0.0005. Complete statistical analysis is reported in Supplementary Table S1.

The histology of the kidney is readily identified in the IMS images because of a differential lipid expression at the cellular level. Figure 4 shows the summation of intensities of the lipid species corresponding to major subclasses of glycerophospholipids and sphingolipids detected in each of the histological areas depicted in Figure 2A (relative abundance of all the species identified is shown in Figures S9–S19). Statistically significant differences have been observed among the kidney structures for most of the lipid subclasses analyzed (Table S1). Notably, a gradient in the expression of SM, SFT, and hexosylceramides (HexCer) exists along the epithelial structures of the nephron, following the path from the cortex to the medulla. While SM and HexCer relative abundances are higher in cortical structures, SFT is mainly found in medullar tubules (MTI and MTII). As a particular case, vascular structures are characterized by a higher abundance of SM, which would explain the similarity between the G and ITS lipid profiles, as depicted in the proximity of the colored G (red) and ITS (orange) areas in the PCA, as shown in Figure 3.

### Distribution Patterns of Selected Lipids in Proximal Tubules

**Figure 5:**
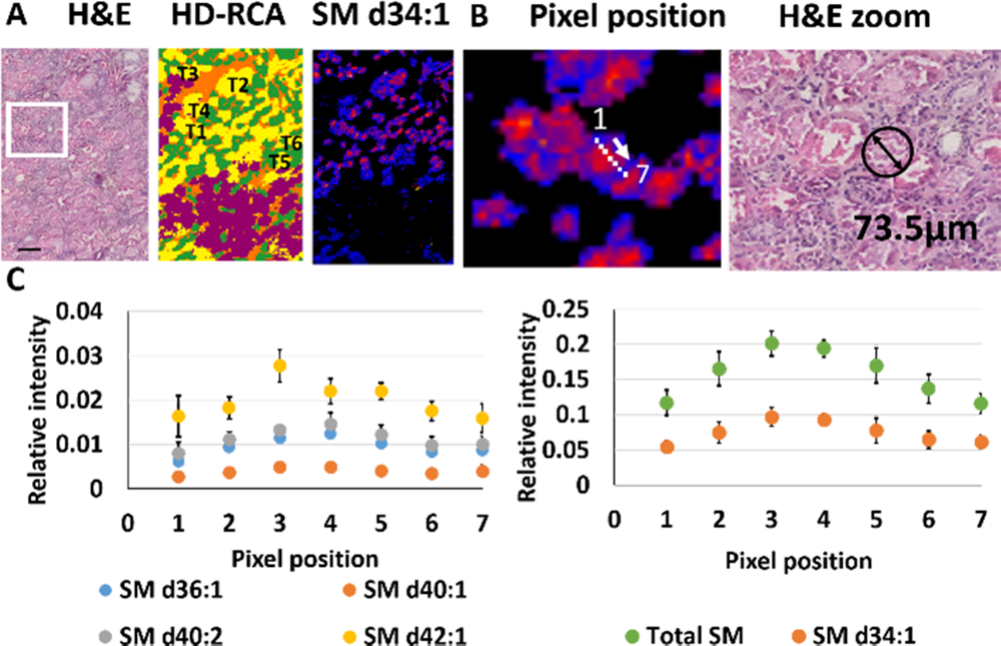
Distribution of sphingomyelins in renal sections. (A) Comparison between a cortical-medullary transition renal sample, its segmentation analysis (HD-RCA), and the distribution of SM d34:1 and d34:2 in proximal tubules II (PTII). (B) Path depicted along the cross-section of a proximal tubule and HE image with the size of a tubule. (C) Changes in SM along the path drawn in B. The left panel shows the variation of SM d34:1 and of the total SM, while the right panel shows the changes in four SM species. There is clearly a higher abundance of SM in the apical membrane. Images were recorded in negative-ion mode at a pixel size of 10 μm. Scale bar = 150 μm. HE, hematoxylin and eosin.

A closer look at the IMS images shows that lipid gradients are also observed along the tubules. The analysis of the cross sections of the specific areas shown in Figure 5 allows for differentiating a significant gradient in the distribution of SMs in proximal tubules. For example, in PTII (cortical yellow areas), SM d34:1 levels are higher in the luminal/apical side, where proximal tubules display a highly developed plasma membrane system,^34^ than in basolateral membranes. A deeper analysis of other segments also points to the existence of subtle differences within the structures reported in this work (Figure S6). Understanding the nature of such structures requires the use of additional techniques, such as IHC, to correlate those lipid signatures with specific cell types or substructures.

## Discussion

The kidney is an organ with a high metabolic activity.^35^ Although there is evidence that different metabolic pathways predominate in each segment of the nephron, very few studies have investigated this issue using imaging techniques in human biopsies.^16^ Regarding lipid metabolism, studies in animal models have described significant differences in the level of some lipid species between the renal cortex and medulla.^17,36^ However, the resolution achieved in such studies did not allow the authors to deeply investigate the different structures of the nephron. This is the first MALDI-IMS systematic study conducted at a high-enough spatial resolution to clearly identify up to eight histological areas based on their in situ lipid signature. In addition, the significant number of samples included in the study, sections from 32 patients, allowed us to demonstrate that the lipid fingerprint of each histological area is maintained among different individuals. A clear correlation between the areas identified based on the lipid profiles and the canonical histological areas of the nephron can be readily established, as shown below.

The renal corpuscle is the blood-filtering component of the nephron and is composed of two structures, the glomerulus and Bowman’s capsule.^34^ MALDI-IMS shows a characteristic lipid fingerprint for the glomerulus and even enables the differentiation of Bowman’s capsule in some samples. Interestingly, interstitial vascular structures surrounding the glomerulus (afferent and efferent arterioles) and from the medullary interstitium (peritubular capillaries and vasa recta) also have a specific lipid profile, which is similar to that of the glomerular capillary tufts. These structures present higher levels of SMs. Additional experiments are needed to establish the functional significance of this lipid family in the vascular structures of the kidney, but it is known that its imbalance could be a biomarker of kidney damage. In this regard, Miyamoto et al.^37^ described, using MALDI-IMS at a spatial resolution of 25 μm, high levels of SM in the glomeruli. Furthermore, they demonstrated in mice and in cell cultures that the accumulation of SM d18:1/16:0 in the glomerulus is involved in the physiopathology of diabetic nephropathy.

The data presented here also demonstrate the existence of proximal tubules with two different lipid fingerprints, which we designated PTI and PTII. The former is located in the renal cortex, near the glomeruli, while the latter is found on both sides of the transition zone. These tubular structures fit well with the classical histology,^38^ which describes two clearly differentiated parts: the convoluted part of the tubule, located more externally in the cortex, and the straight part, which reaches the outer medulla (Figure 2). The characterization of the lipidome of proximal tubules is important to better understand the neoplastic processes that originate in this part of the nephron, such as clear cell renal cell carcinomas (ccRCC) and papillary renal cell carcinomas (pRCC).^24^ A recent study using single-cell RNA sequencing demonstrated that the cells of the first segment of the convoluted part have common transcriptional characteristics with ccRCC and pRCC cells.^39^ Studies specifically designed to this purpose would allow us to demonstrate if these common genomic roots also have a reflection in the lipid signature of both PTI cells and tumor cells. The association between the lipidomic profile of ccRCC and the Bowman’s capsule should not be discarded either because this nephron structure has been recently proposed as another possible origin of the most common subtype of RCC.^25,40^

Limited to the cortex, we detected another tubular structure with a specific lipid profile, labeled as CDT. In the inner part of the cortex and in the outer medulla, there was another class of tubule with a specific lipid fingerprint (CMT). Finally, limited to the medulla, especially in the inner areas, we detected two other tubule types, MTI and MTII. These four tubular structures present similarities in their lipid fingerprints. However, there is an important characteristic that distinguishes them: as the tubules progress into the medulla, the levels of SFT increase. This gradient between the cortex and medulla was previously described in the rodent kidney.^17,36^ The presence of this family of lipids is known to be necessary for maintaining a high concentration of ammonium in the medullary interstitium and for a correct function of medullary collecting ducts, which are responsible for the secretion of ammonium to urine, both under basal conditions and under metabolic acidosis.^41^

**Figure 6:**
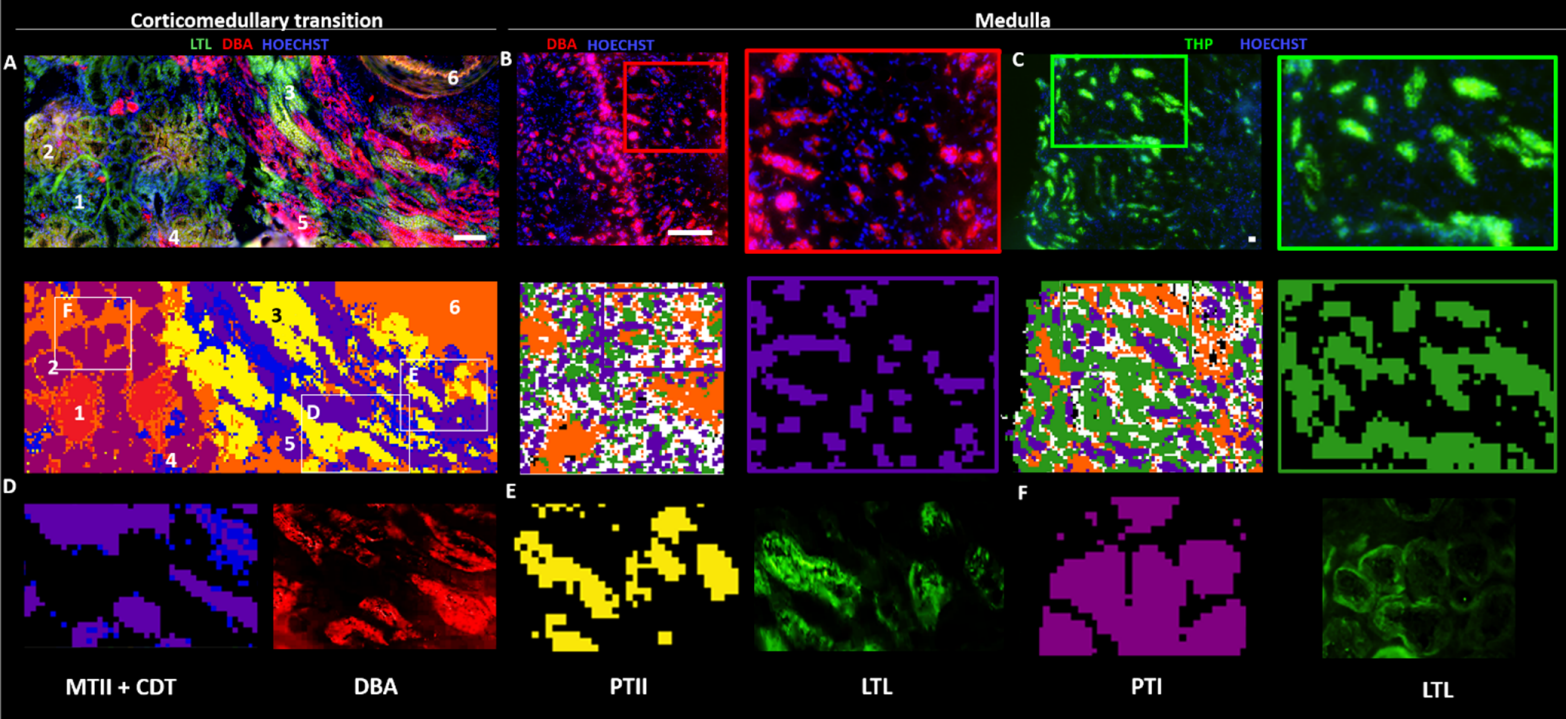
Comparison between the IF staining and the segmentation analysis of the IMS experiment carried out over the same section. (A) Section of a cortical-medullary transition. Segment-specific tubular markers used are as follows: proximal tubule, *Lotus tetragonolobus* lectin (LTL); DCTs and collecting ducts, *Dolichos biflorus* agglutinin (DBA). The correspondence of the numbers are (1) glomerulus; (2) proximal tubule I (PTI); (3) proximal tubule II (PTII); (4) cortical distal tubule (CDT); (5) collecting duct (MTII); and 6) capillary; (B) Comparison between the IF of a section of a medullary biopsy and the corresponding MS image. DBA-positive collecting duct tubules correlate with the purple segment, which is attributed to MTII based on its lipid signature. (C) Comparison between the segmentation analysis of an IMS experiment over a section of the medulla and the THP (Horse Tamm Horsfall Protein)/Hoesch IF image. The THP-positive tubules correspond to thick ascending Henle limbs and correlate with the cluster correctly attributed to CMT (green segment) in the IMS experiment; (D–F) Zoomed areas of the figures in panel A showing the good correlation between the areas highlighted in the IF experiment and the segments found by IMS. Scale bar = 150 μm. An enlarged version of this figure can be found in the Supporting Information (Figure S21).

One of the problems faced during this work to correlate the collection of lipid fingerprints detected by IMS with well-known histological areas was the difficulty in identifying the architecture of the tissue with a level of detail similar to that offered by IMS. One must keep in mind that the application of this technique is limited to fresh tissues; therefore, it was not possible to use fixed tissue sections or sections embedded in paraffin, which allows pathologists to discern the histological structures with higher precision. Therefore, additional IF experiments were carried out to further test the identity of the sections of the nephron located using lipid signatures. Figure 6 shows the comparison between the IF staining of various segment-specific tubular markers and the segmentation of the IMS experiment recorded over the same tissue. In the corticomedullary transition sample (Figure 6A), proximal tubules are LTL-positive, and they correlate with the dark red and yellow IMS segments named PTI and PTII (numbered as 2 and 3 in the figures), confirming the assignment based on the lipid signature. Additionally, distal convoluted tubules (DCTs) and collecting ducts are DBA-positive and show a clear correlation with the blue and purple segments named as CDT (number 4) and MTII (number 5). Furthermore, the same correlation between MTII and the DBA-positive tubules is shown in the medullary section, allowing us to confirm the assignment of these tubules as collecting ducts.

Figure 6C also shows the THP (Horse Tamm Horsfall Protein)/Hoesch IF experiment over a section of the kidney medulla. The THP-positive tubules (in green) correspond to thick ascending Henle limbs. These structures perfectly match the location of the green segments in the corresponding segmentation image, which were attributed to CMT (see Figure 2). As it can be seen, descendent thin limbs are not stained in any of the images, and they very well match the attribution of the white segment in the IMS experiments to this section of the nephron.

The location of the segments identified in the renal tissue and the differences in the SFT expression levels allow us to hypothesize about these four tubular structures detected by MALDI-IMS (see Figure 2): MTII are present in higher proportion in the inner medulla and have the highest level of SFTs, suggesting that they correspond to the medullary portion of the collecting ducts, the most abundant structures in this area of the kidney.^38,42^ MTI are also found in the inner medulla, but in lesser proportion, and have high levels of SFTs, indicating that they could correspond to the thin descending limbs of Henle’s loops of the yuxtamedullary nephrons. Because of its high presence at the corticomedullary level and their content in SFTs, which is intermediate between those of the exclusively cortical and medullary tubules, CMT could correspond to the thick ascending limb. Finally, CDTs are only found in the renal cortex and have lower SFT levels, which suggests that they correspond to the cortical portion of the distal nephron, which comprises DCT, connecting tubules (CNTs), and cortical collecting ducts (CCT).^42^ All these assumptions match very well with the IF experiments presented in Figure 6.

Comparison between the relative abundance of these structures in the sections analyzed in the present work and the ratios reported in the mouse kidney^42,43^ shows similar proportions in both cases. For example, proximal tubules are the most abundant epithelial cell type in the mouse kidney, amounting to 44%, whereas the lipid fingerprints of PTIs and PTIIs reached 56% of the total detected epithelial structures in the human kidney. The cortical and medullary thick ascending limb (CTAL and MTAL) cells represent 21% in mouse kidney, while according to this work, CMTs represent 14% in the human kidney. Lipid fingerprints of CDTs represent 13% of the area of our sections, while the cortical portion of the distal nephron in the mouse kidney constitutes around 22% of total epithelial cells. Furthermore, cells from thin limbs and medullary collecting ducts in the mouse kidney amount to 6 and 7% of the total cellularity, respectively, whereas lipid fingerprints of MTI and MTII represent 4 and 13% of our sections, respectively. These similarities between our data and the previously reported cell-type distributions^42^ in mice^42^ also support the assignment proposed here.

The high spatial resolution achieved in our MALDI-IMS experiments allowed us to take a step forward and to analyze the variation of lipid relative abundance across the sections of PTII. For example, there is a clear gradient in the relative abundance of SMs from the apical membrane to the basal side of the proximal tubules (Figure 5). These lipids may play an important role in sodium-coupled reabsorption mechanisms.^44^ Furthermore, it is tempting to speculate that the defined eight structures with a characteristic lipid fingerprint could correspond to the previously described cell subpopulations from each part of nephron^42^ or to the cells from the same subpopulation in different metabolic states (Figure S6). Future studies of isolating kidney cells, analyzing their lipidome by MALDI-IMS, and associating these results with the transcriptome of each subpopulation will shed light on these questions.

## Conclusions

Here, we present a detailed study on the architecture of the human kidney by MALDI-IMS, which can be taken as a kind of molecular histology. Using this technology, it is possible to determine lipid distribution maps that describe the histology of the tissue from a metabolic point of view, without the drawbacks associated to cell-isolation procedures. In comparison with the optical images, the IHC experiments and the description in the literature of the nephron structure allowed us to demonstrate that at least seven different parts of the nephron present characteristic and unique lipid fingerprints, which are also different from those of the interstitium. The lipid signatures described are maintained among different individuals. The differences in lipid profiles are related to the differences in the cell composition between the histological areas. Upon further exploiting the high spatial resolution achieved in the experiment, we can demonstrate the existence of gradients in the lipid expression along the cells of the different segments of the nephron. This work is a starting point for further studies in which precise identification of even more histological areas of the nephron by comparison with immunocytochemistry images will be tackled. It also sets the foundation for further studies on the metabolic basis of the diseases affecting the human kidney.

## Supporting Information Available

MALDI-IMS experiments; data analysis; high-performance liquid chromatography/mass spectroscopy; IF microscopy; statistical analysis of the lipid fingerprints; statistical analysis of the lipid family fingerprint of the discovery group; acylic chains of the detected lipids species; and additional figures (PDF)
