# Supplementary material for: High-Resolution Human Kidney Molecular Histology by Imaging Mass Spectrometry of Lipids
Source: Anal Chem. 2021 Jun 30;93(27):9364–72. doi: 10.1021/acs.analchem.1c00649 (PMC8922278; doi:10.1021/acs.analchem.1c00649)
Supplement: Supplementary file 1 — ac1c00649_si_001.pdf [file ac1c00649_si_001.pdf]

Supplemental Information for:

HIGH RESOLUTION HUMAN KIDNEY MOLECULAR HISTOLOGY BY IMAGING MASS SPECTROMETRY OF LIPIDS

Lucía Martín-Saiz[1], Lorena Mosteiro[2], Jon Danel Solano-Iturri[2,3], Yuri Rueda[4], Javier Martín-Allende[1], Igone Imaz[2], Iván Olano[5], Begoña Ochoa[4], Olatz Fresnedo[4], José A. Fernández[1*] and Gorka Larrinaga[3,4,6*]

[1]Department of Physical Chemistry, Faculty of Science and Technology, University of the Basque Country (UPV/EHU), Barrio Sarriena, s/n, Leioa 48940, Spain.

[2]Service of Anatomic Pathology, Cruces University Hospital, University of the Basque Country (UPV/EHU), Cruces (Barakaldo) 48903, Spain.

[3]BioCruces Health Research Institute, Cruces (Barakaldo) 48903, Spain.

[4]Department of Physiology, Faculty of Medicine and Nursing, University of the Basque Country (UPV/EHU), Barrio Sarriena, s/n, Leioa 48940, Spain.

[5]Service of Urology, Cruces University Hospital, Cruces (Barakaldo) 48903, Spain.

[6]Department of Nursing I, Faculty of Medicine and Nursing, University of the Basque Country (UPV/EHU), Barrio Sarriena, s/n, Leioa 48940, Spain.

[(*)]Corresponding authors:

Gorka Larrinaga.

Department of Nursing I. Faculty of Medicine and Nursing. University of the Basque Country (UPV/EHU). Barrio Sarriena, s/n, Leioa 48940, Bizkaia, Spain. Phone: +34.94.601.8076.

e-mail: gorka.larrinaga@ehu.eus

José Andrés Fernández.

Department of Physical Chemistry, Faculty of Science and Technology, University of the Basque Country (UPV/EHU), Barrio Sarriena, s/n, Leioa 48940, Spain. Phone: +34.94.601.5387

e-mail: josea.fernandez@ehu.es

Additional Methods

MALDI-IMS experiments

Histological sections from 32 different patients were prepared and analyzed by MALDI-IMS as described in Garate et al[1]. DAN was used as matrix for negative-ion detection and deposited with the aid of our in-house designed sublimator[2]. Sections of kidney biopsies were scanned in negative-ion mode, using the orbitrap analyzer of a MALDI-LTQ-Orbitrap XL (Thermo Fisher, San Jose, CA, USA). The MALDI source used in this study was an in-house modified version of the originaly provided by the manufacturer,[3] which is equipped with a N_2 laser (LTB, Berlin, model MNL 100, 100 μJ max power, elliptical spot, 60 Hz repetition rate), and a very simple optical arrangement, consisting of two mirrors and a single focusing lens of f = 125 mm. After the modification, the laser spot size at the sample was < 10 μm.

Data were acquired with a mass resolution of 60000 at *m/z* = 400. Two microscans of 10 laser shots were recorded for each pixel and the raster size used was 10 μm. The observation window was chosen as a compromise between covering a representative portion of lipid classes while maintaining high enough sensitivity as to detect low-abundance species. Therefore, it was set to *m/z* = 550-1000. In this way, the appearance window of most glycerophospholipids and sphingolipids was covered, at expenses of missing other lipid classes such as cardiolipins or fatty acids. Spectra were aligned and analyzed using in-house developed software, built in Matlab (MathWorks, Natick, USA). Lipid assignment was based on the comparison between the experimental *m/z* and the species in the software's database (> 33,000 lipid species plus adducts) and in the lipid maps database (www.lipidmaps.org). Mass accuracy was always better than 9 ppm and was typically better than 3 ppm. For *m/z* channels with several candidates for the assignment, "on-tissue" MS/MS and MS^3 was carried out to reach a univocal assignment. Moreover, fatty acid chains were obtained from an UHPLC/ESI-MS/MS study of lipid homogenates from 11 human renal samples. With this procedure, it is not possible to distinguish between ether and vinyl-ether lipids.

Detailed description of data processing may be found in ref 1, but briefly, the spectra were normalized using a total ion current (TIC) algorithm, and aligned using the Xiong method during the parsing stage[4]. All peaks with intensity values lower than 0.5% of the intensity of the strongest peak were filtered to reduce the number of *m/z* values and speed up the analysis. Unwanted peaks due to fragmentation, matrix and other species that only introduce noise in the analysis were removed[5]. Only reproducible lipid species were used in multiple comparative studies to minimize the inter-patient variability. For that, the species whose standard deviation was larger than 80% of the peak intensity and the ones that were detected in less than 80% of a

sample group were also filtered. Regarding lipid abundance, the MALDI-IMS protocol used in this work only gives relative abundances within each lipid class. This means that signal intensity cannot be translated directly into lipid abundance, whereby one must limit the discussion to analyze relative variations in the abundance of the species of a given family.

Data Analysis.

Data from each section were analyzed using segmentation algorithm (HD-RCA) to isolate and identify the lipid signatures of each histological area in the section. The fundamentals were already described in a previous publication[6]. The segmentation algorithm, *RankCompete*[7], is based on the properties of *Markov Chains*[8] to define *Random Walkers*[9] competing to divide the imaging experiment into a number of segments with enough variability to describe independent histologic areas. The starting point for each walker was decided using a variation of the Divisive Analysis algorithm (DIANA). Thus, the final software is a segmentation algorithm based on DIANA and using RankCompete as split function. In this way, it is possible to use the correlation value between the segments to assign a color to each segment using a color scale and 1-correlation between the segments. Thus, the two segments that present the lowest correlation occupy the two extremes of the scale and those segments with more similar average spectra receive colors that are closer in the scale.

To establish the number of segments on each image, an additional heuristic approach was used; the initial number of segments was set in 5 for cortical and medullary samples and 8 for cortical-medullary transition sections. Then, the segments suggested by the algorithm were verified by examining their correlation. The segments whose correlation was higher than 95% were grouped together. In the end, the total number of segments found in all the samples was 131, divided in eight histologic areas. The signatures from the final histological areas were later used in subsequent multi-experiments analysis.

To statistically evaluate the differences in the lipid fingerprints among the eight identified histological areas, Levene test, ANOVA univariate statistical analysis and Tukey /Games Howell post hoc were computed using SPSS Statistics 17.0 (IBM, Armonk, NY, USA)[10]. Levene test determines the homogeneity (H_0 = groups have equivalent variance) to choose the post hoc method: Tukey if Levene $p \geq 0.05$ and Games-Howell if Levene $p \leq 0.05$. PCA analysis and separation models were carried out by Orange Biolab 2.7.8 (Ljubljana, Slovenia)[11].

HPLC-MS/MS

Internal standards: Splash Lipidomix, Cer/Sph mixture I, Cardiolipin Mix I, L-carnitine 24:0(d4), monosulfogalactosyl-β-ceramide d18:1/12:0 (NH_4 salt) and oleic acid (d9) were purchased from Avanti Polar Lipids (Alabaster, Alabama).

Biopsies from 11 patients were washed twice in ice-cold PBS, weighed and randomly pooled to generate three samples containing 60-80 mg of tissue. Samples were homogenized on ice in 10 vol PBS with 3 bursts of 10 seconds on-and-off using a Polytron homogenizer (Kinematika, Switzerland). Protein in the homogenate was estimated in triplicate by the bicinconinic acid protein assay (Thermo Scientific, IL, USA) using BSA as standard. Lipids from aliquots of the homogenates (~2.5 mg protein) were thoroughly extracted following a Bligh and Dyer[12] protocol, adapted to small volume samples[13]. Solvents were evaporated in a Savant SpeedVac concentrator (Thermo Scientific, IL, USA) and the dried extracts were immediately placed into N_2 gas atmosphere and stored at -80 ºC until use.

Prior to UHPLC, lipid extracts were dissolved in 150 µL of MeOH:Toluene (9:1, v/v), transferred to maximum recovery chromatography vials using a micropipette and injected into the UHPLC-Q-Orbitrap HF-X. Quality control samples (QC) were prepared by pooling 25 µL aliquots from all the samples and it was injected at the beginning, the end and during the analysis sequence to estimate the variation in signal intensity, establish the stability of the analysis and identify possible tendencies. Blank samples were MeOH:Toluene (9:1, v/v) and were injected at the beginning and at the end of the run.

Chromatography was carried out by using an ACQUITY UHPLC HSS T3 2.1x 100 mm, 1.8 µm (Waters, Milford, MA, USA), heated to 65ºC. Mobile phases consisted of acetonitrile and water with 10 mM ammonium acetate (40:60, v/v, phase A) and acetonitrile and isopropanol with 10 mM ammonium acetate (10:90, v/v, phase B). Separation took 13 min under the following conditions: 0–10 min, linear gradient from 40% to 100% B; 10–11 min, 100% B; and finally, re-equilibration of the system with 40% B (v/v) for 2 min, prior to the next injection. Flow rate was 0.5 ml/min and injection volume was 7.5 µl. All samples were kept at 4 ºC during the analysis.

All UHPLC-MSE data were acquired on a SYNAPT G2 HDMS, with a Q-ToF configuration (Waters MS Technology, MA, USA) equipped with an electrospray ionization (ESI) source that can be operated in both positive- and negative-ion modes. The capillary voltage was set to 0.7 kV (ESI+) or 0.5 kV (ESI−). Nitrogen was used as desolvation and cone gas, at flow rates of 900 l/h and 30 l/h, respectively. The source temperature was 120 ºC, and the desolvation temperature was 400 ºC. Further details may be found in ref 13.

An acquisition mass window of 250-200 Da was used both in positive- and negative-ion modes. Mass resolution was set to 120 000 (FWHM) at m/z = 200. MS/Ms data collection was carried out with and isolation window of 1 Da.

Chromatogram alignment, identification and quantification of the lipid species detected was carried out using LipidSearch Ver. 4.2.2, using a 5 ppm mass tolerance for the parent ion and for

the fragment. All main glycerophospholipid, esfingolipid and glycerolipid families were considered during assignment, together with cholesterol esters and acyl carnitines.

Immunofluorescence microscopy

Sections analyzed by MALDI-IMS were fixed with 4 % paraformaldehyde in phosphate buffered saline) (Sigma Aldrich, MO,USA) for 10 min at room temperature and washed in cold methanol/acetone (1:1) (Minimum purity 99.5%,Scharlab, BA,Spain) for 5 min in order to remove the MALDI matrix. Corticomedullary sections were stained with Lotus tetragonolobus lectin (LTL)-fluorescein and dolichos biflorus agglutinin (DBA)-rhodamine (both at 1:33 dilution) (Vector Laboratories,CA,USA) essentially as described[14]. In addition to DBA, medullary tissue sections were immunostained to detect tamm horsfall glycoprotein (THP). Samples were incubated at room temperature for 2 h with mouse anti-human THP (B-2) primary antibody (1:200) (Santa Cruz Biotechnology,TX,USA) and, afterward, with Alexa Fluor 488-labeled goat anti-mouse IgG secondary antibody (1/200) (Invitrogen) for 1 h. All the sections were counterstained with 10 µg/ml Hoechst-33342 (Invitrogen,CA,USA). After mounting (ProLong Gold, Life Technologies,CA,USA), stained sections were analyzed with a Nikon-Ti-U fluorescence microscope (Nikon instruments,NY,USA) and processed with Nikon Ds-Qi2 and Nikon DS-Fi1 softwares.

References

1. Garate, J.; Fernández, R.; Lage, S.; Bestard-Escalas, J.; Lopez, D. H.; Reigada, R.; Khorrami, S.; Ginard, D.; Reyes, J.; Amengual, I. Imaging mass spectrometry increased resolution using 2-mercaptobenzothiazole and 2, 5-diaminonaphtalene matrices: application to lipid distribution in human colon. *Anal. Bioanal. Chem.* **2015**, *407*, 4697-4708.

2. Fernández, R.; Garate, J.; Martín-Saiz, L.; Galetich, I.; Fernández, J. A. Matrix Sublimation Device for MALDI mass spectrometry imaging. *Anal. Chem.* **2018**, *91*, 803-807.

3. Montero, R.; Abad-García, B.; Garate, J.; Martín-Saiz, L.; Barceló-Coblijn, G.; Fernández, J. A. Improving Spatial Resolution of a LTQ Orbitrap MALDI Source. *J. Am. Soc. Mass Spectrom.* **2020**, *31*, 1755-1758.

4. Xiong, X.; Xu, W.; Eberlin, L. S.; Wiseman, J. M.; Fang, X.; Jiang, Y.; Huang, Z.; Zhang, Y.; Cooks, R. G.; Ouyang, Z. Data processing for 3D mass spectrometry imaging. *J. Am. Soc. Mass Spectrom.* **2012**, *23*, 1147-1156.

5. Garate, J.; Lage, S.; Martín-Saiz, L.; Perez-Valle, A.; Ochoa, B.; Boyano, M. D.; Fernández, R.; Fernández, J. A. Influence of Lipid Fragmentation in the Data Analysis of Imaging Mass Spectrometry Experiments. *J. Am. Soc. Mass Spectrom.* **2020**, *31*, 517-526.

6. Fernández, R.; Garate, J.; Tolentino-Cortez, T.; Herraiz, A.; Lombardero, L.; Ducrocq, F.; Rodriguez-Puertas, R.; Trifilieff, P.; Astigarraga, E.; Barreda-Gómez, G. Microarray and Mass Spectrometry-Based Methodology for Lipid Profiling of Tissues and Cell Cultures. *Anal.Chem.* **2019**, *91*, 15967-15973.

7. Cao, L.; Jin, X.; Yin, Z.; Del Pozo, A.; Luo, J.; Han, J.; Huang, T. S. Simultaneous ranking and clustering of information networks. *Neurocomputing.* **2012**, *95*, 98-104.

8. Markov Chains. Kemeny, J. G. and Snell, J. L. *Markov Chains,* ed.; Springer-Verlag, New York: 1976.

9. Random Walk. Spitzer, F. *Principles of Random Walk,* ed.; Springer Science & Business Media: **2013**.

10. Corportation I. IBM SPSS Statistics for Windows *NY: IBM Corp.* **2017.**

11. Orange: data mining toolbox in Python. Demšar, J.; Curk, T.; Erjavec, A.; Gorup, Č; Hočevar, T.; Milutinovič, M.; Možina, M.; Polajnar, M.; Toplak, M.; Starič, A. *the Journal of machine Learning research.* **2013**, *14*, 2349-2353.

12. Bligh, E. G.; Dyer, W. J. A Rapid Method of Total Lipid Extraction and Purification. Canadian Journal of Biochemistry and Physiology. **37,** 911-917 (1959).

13. Fernández, R.; Garate, J.; Abad, B.; Ochoa, B.; Fernández, J. A. Mapping Lipid Distribution in Rat Sciatic Nerve using Imaging Mass Spectrometry. In *Myelin: Methods and Protocols,* Woodhoo, A., Ed.; Springer New York, New York, NY, **2018**; p. 51.

14. Jonay Poveda , Ana B Sanz, Susana Carrasco, Marta Ruiz-Ortega, Pablo Cannata-Ortiz, Maria D Sanchez-Niño, Alberto Ortiz. Bcl3: a regulator of NF-κB inducible by TWEAK in acute kidney injury with anti-inflammatory and antiapoptotic properties in tubular cells *Exp.Mol.Med.* **2017**,*49*, e352-e352.

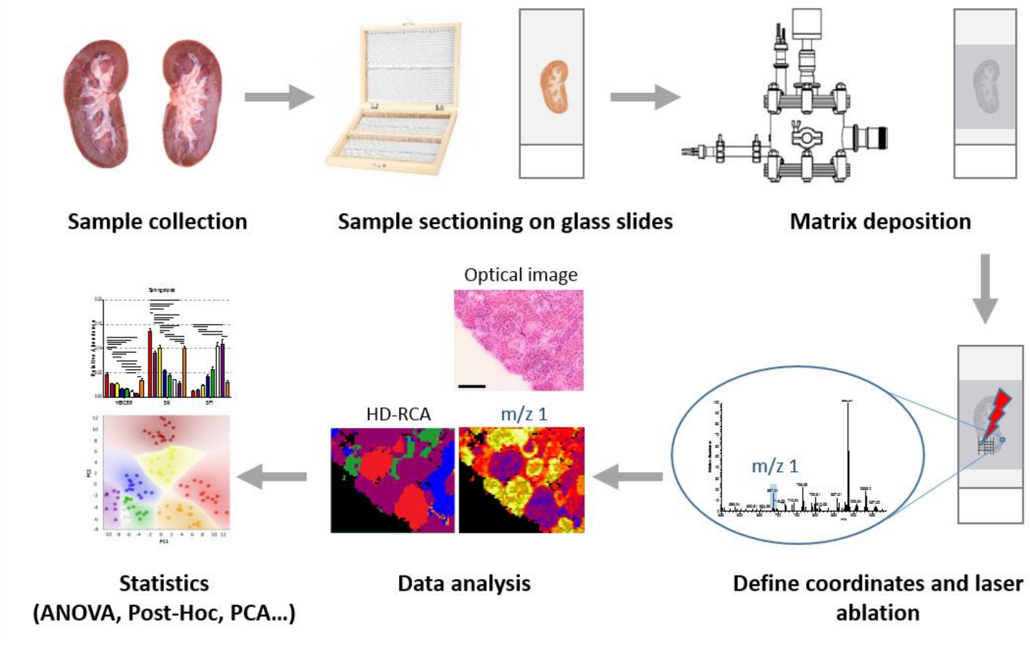

Figure S1. Workflow of a matrix-assisted laser desorption/ionization imaging mass spectrometry experiment. After collection, renal samples were snap-frozen in liquid nitrogen, sectioned, and thaw-mounted on plain glass microscope slides. Slides were then coated with the matrix, which enables lipid extraction. Then, a grid of coordinates was defined over the tissue and a mass spectrum was acquired at each coordinate. The combination of all the spectra permits to visualize the distribution of all the mass channels detected. Once the IMS experiment was completed, matrix was removed and the slide was hematoxylin and eosin stained for structure identification. Finally, segmentation algorithm (HD-RCA) and further statistical analysis were used to identify the tissue's architecture from its lipid fingerprint, and to identify the lipid species whose relative abundances differed between the classified regions. ANOVA, analysis of variance; PCA, principal component analysis.

Cortex samples

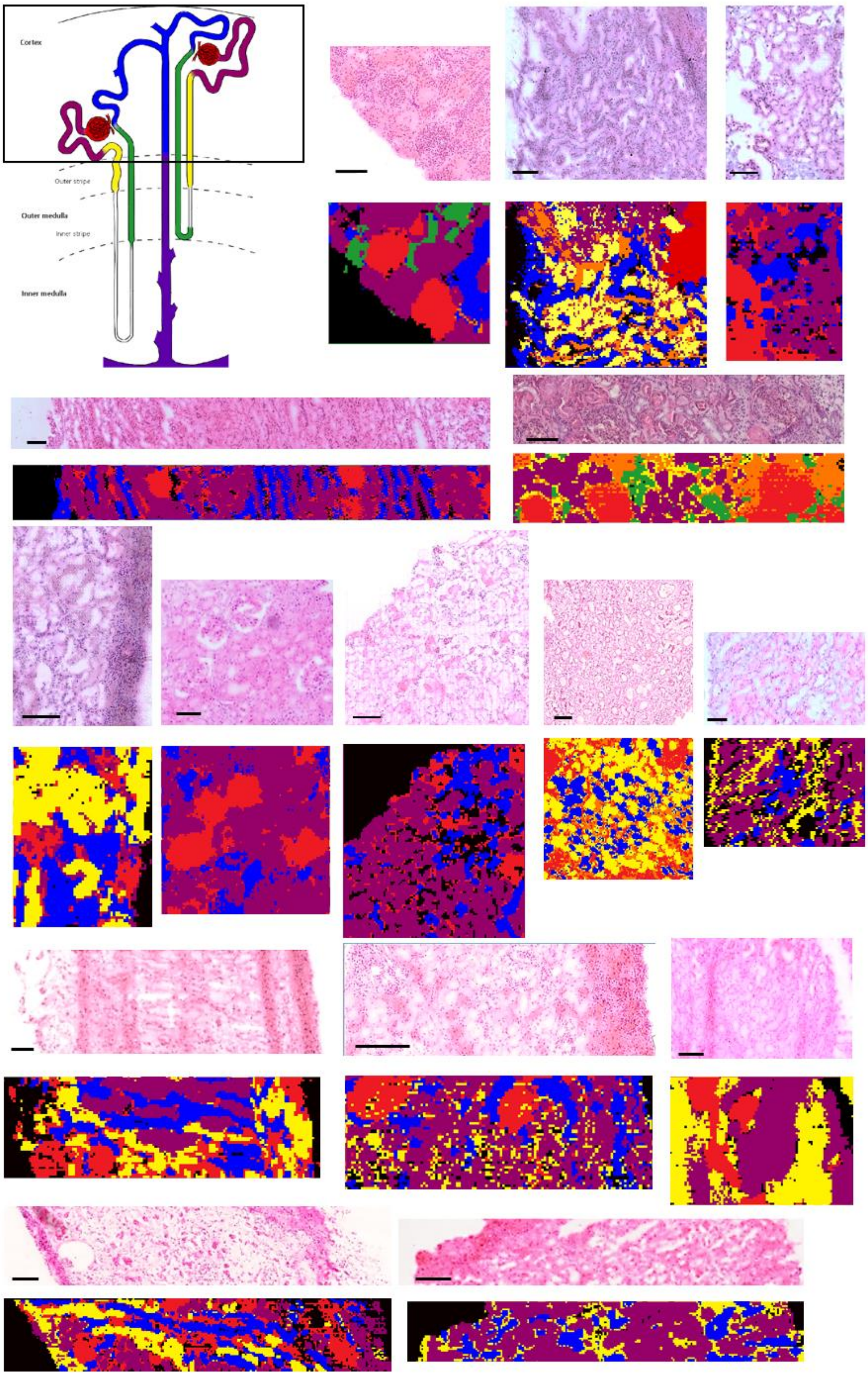

Figure S2. Comparison between H&E optical images of 15 human cortical renal samples and their corresponding image of the segmentation analysis based on their lipid fingerprint obtained by IMS. The colored nephron scheme clarify the renal area which the samples belong. The Images were recorded in negative-ion mode at a pixel size of 10µm. Scale bar=150 µm. H&E, hematoxylin and eosin.

Corticomedullary samples

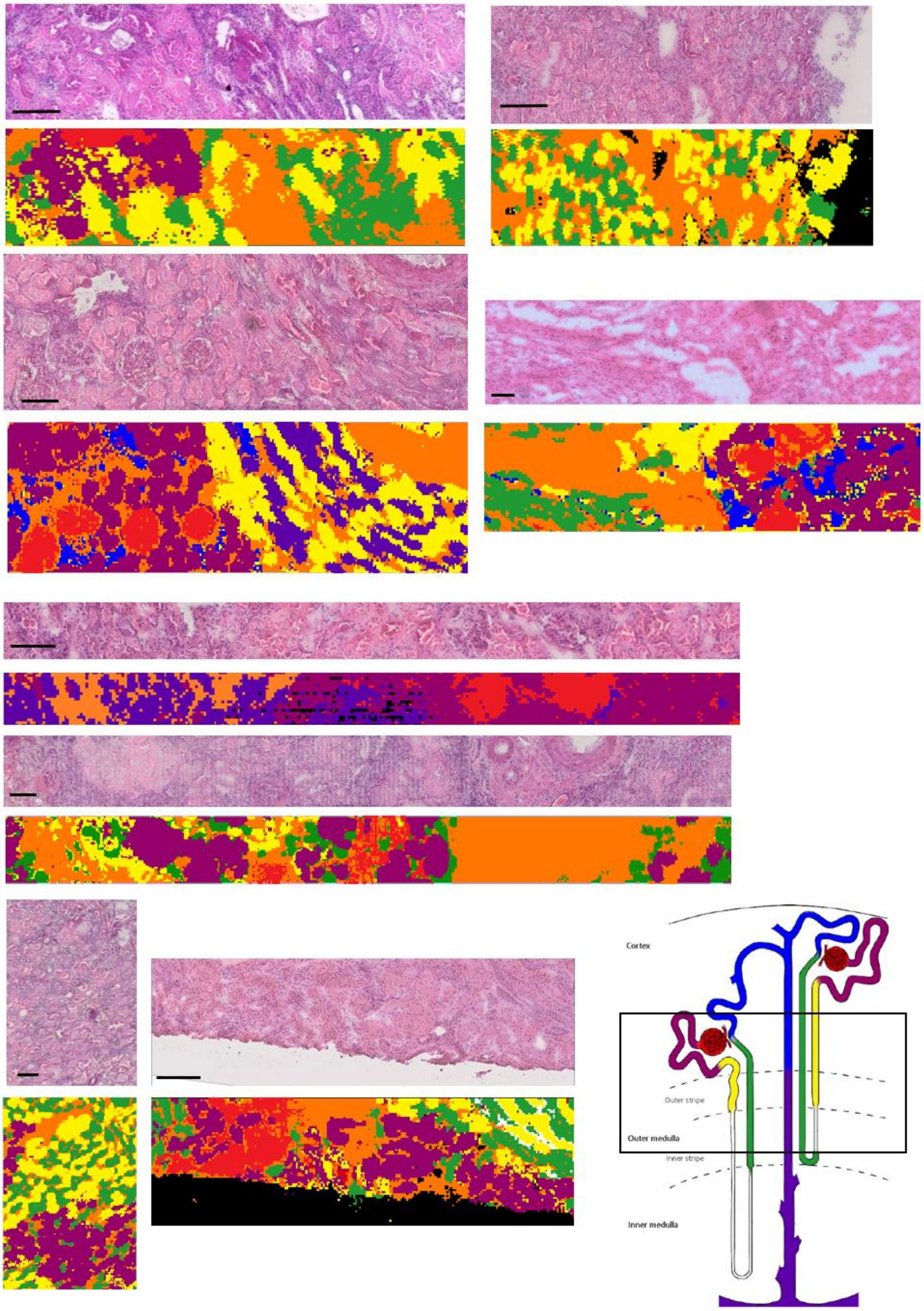

Figure S3. Comparison between H&E optical images of 8 human corticomedullary renal samples and their corresponding image of the segmentation analysis based on their lipid fingerprint obtained by IMS. The colored nephron scheme clarify the renal area which the samples belong. The Images were recorded in negative-ion mode at a pixel size of 10µm. Scale bar=150 µm. H&E, hematoxylin and eosin.

Medullary samples

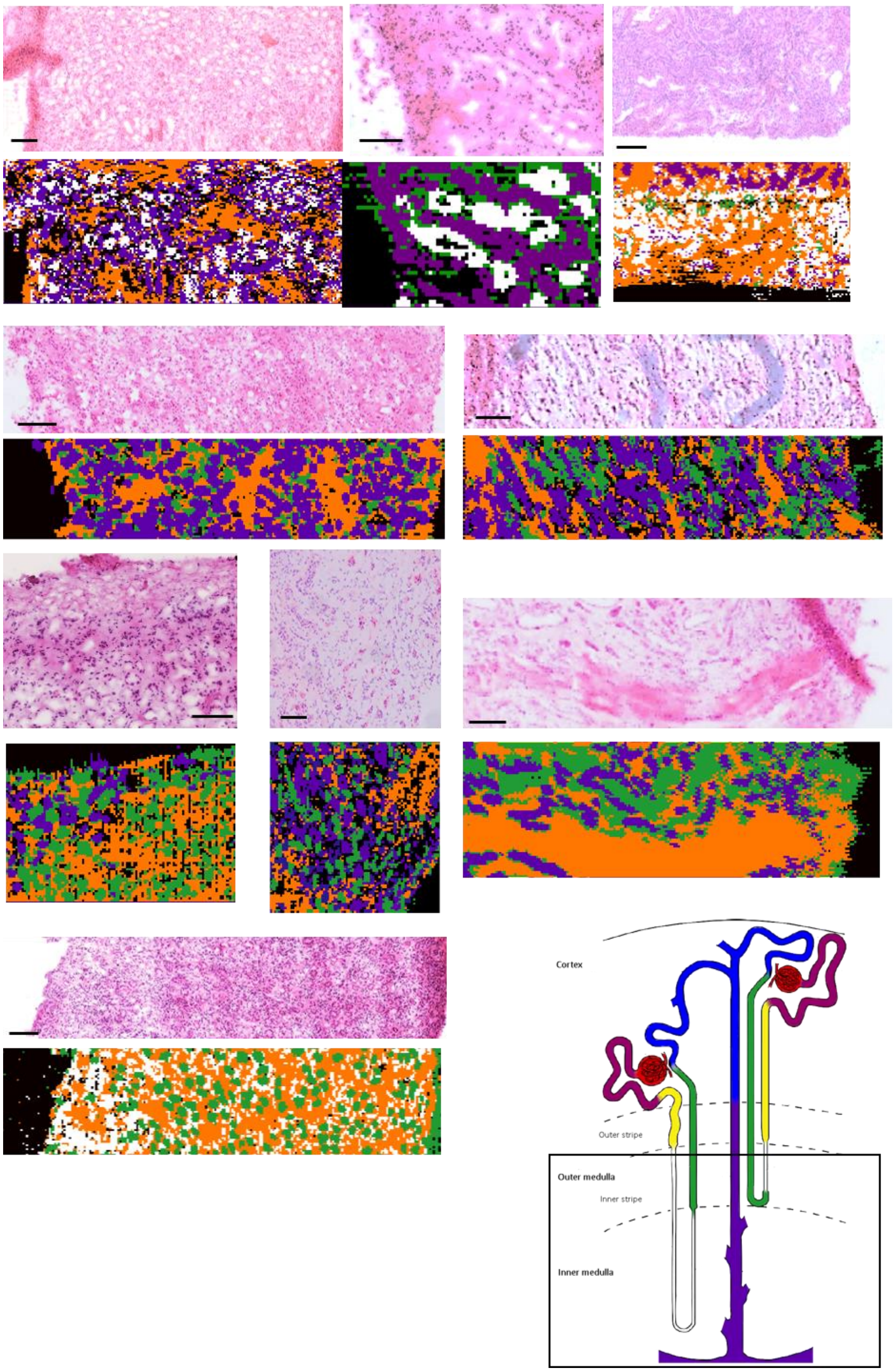

Figure S4. Comparison between H&E optical images of 9 human medullary renal samples and their corresponding image of the segmentation analysis based on their lipid fingerprint obtained by IMS. The colored nephron scheme clarify the renal area which the samples belong. The Images were recorded in negative-ion mode at a pixel size of 10µm. Scale bar=150 µm. H&E, hematoxylin and eosin.

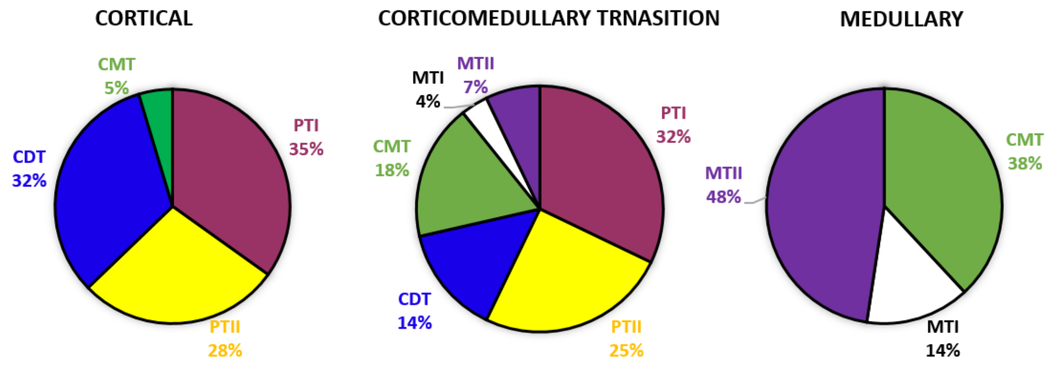

Figure S5. Percentage of the total area covered by each histologic structure in cortex, corticomedullary and medullary samples, excluding the vascular structures (G and ITS).

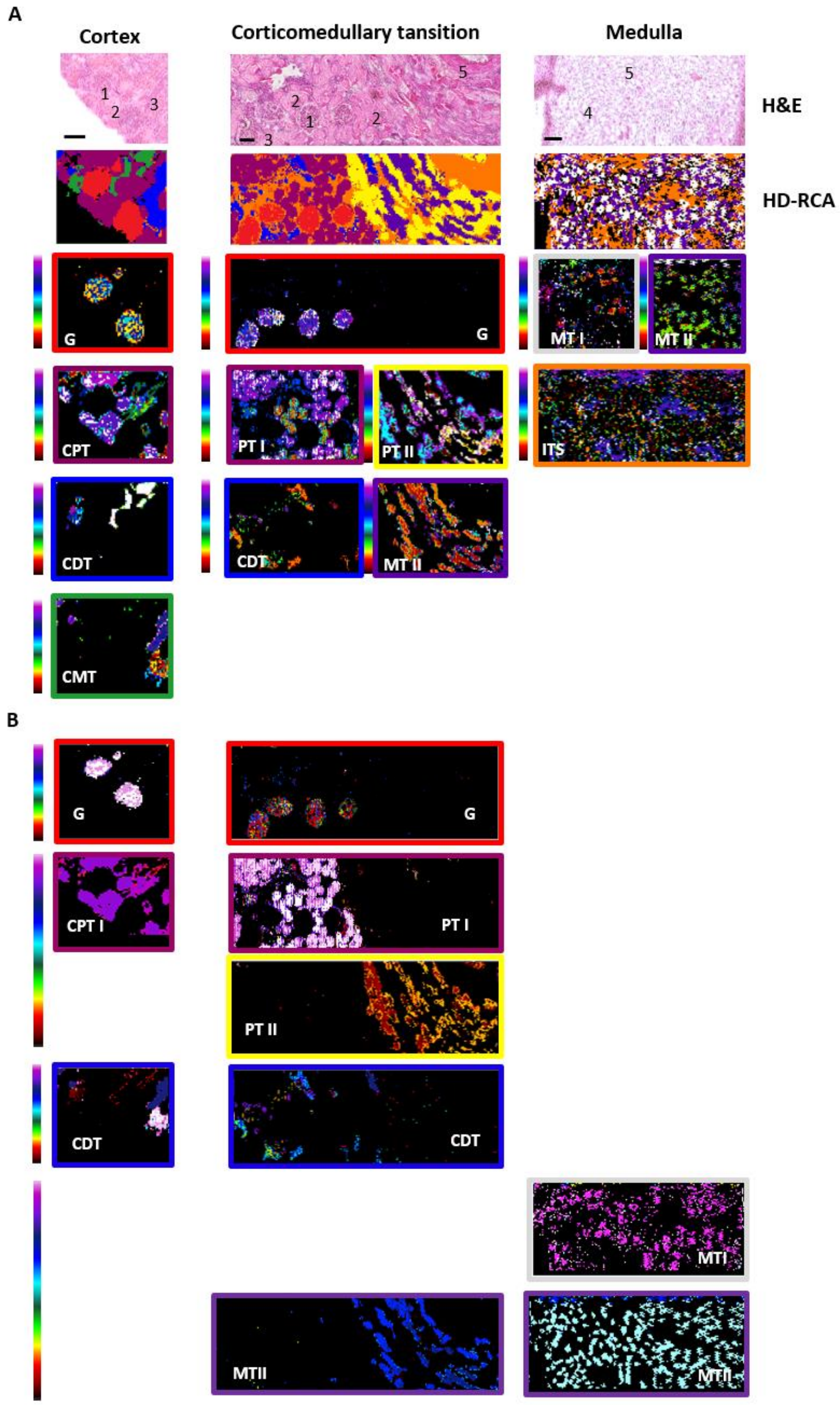

Figure S6. Separate renal segmentation of each IMS histological area of one cortical, corticomedullary transition and medullary sample. Number of segments was set in 10. Clustering results are represented with a cluster separation of 1-correlation between groups, meaning that the clusters with more similar lipid fingerprint receiving colors that are closer in the colorbar. **(A)** Each image has its own scale bar.**(B)** Each structure has its own bar scale which makes easy to see the differences among the same structure in different samples.

Abbreviations; G, Glomeruli; PTI, Proximal tubule I; PTII, Proximal tubule II; CDT, Cortical distal tubule; CMT, Corticomedullar tubule; MTI, Medullary distal tubule I; MTII, Medullary distal tubule II; ITS, interstitial structure. Images were recorded in negative-ion mode at a pixel size of 10 µm. Scale bar = 150 µm. H&E, hematoxylin and eosin, HD-RCA, Segmentation algorithm used.

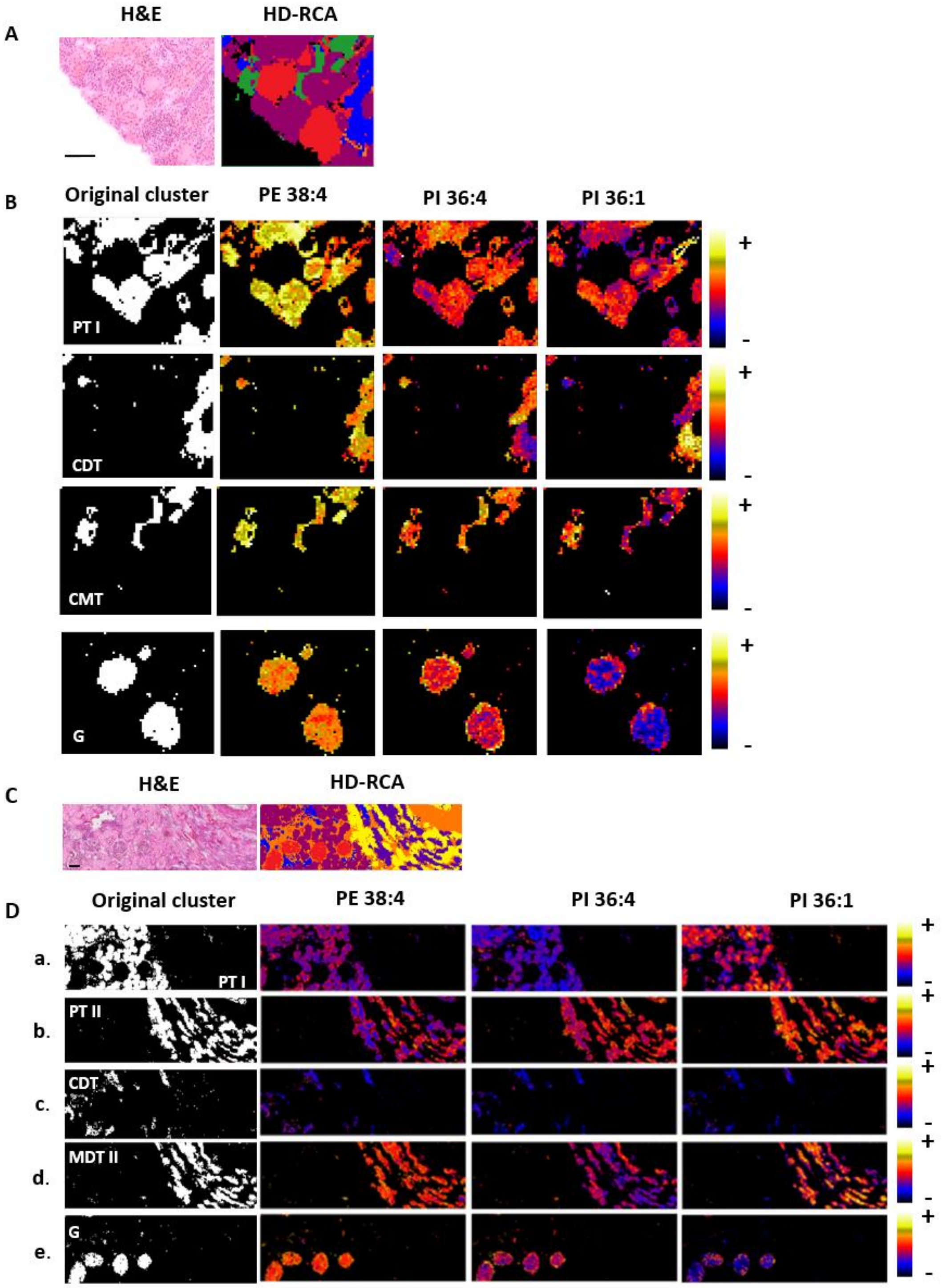

Figure S7. Histological segmentation of human renal sample. **(A)** Comparison between H&E optical image of cortical renal sample and segmentation analysis of the lipid fingerprint at each pixel (HD-RCA)*. **(B)** Separate renal segments and distribution of PE 38:4, PI 36:4 and PI 36:1 lipids over each renal cluster. **(C)** Comparison between H&E optical image of corticomedullary transition renal sample and segmentation analysis of the lipid fingerprint at each pixel (HD-RCA)*. **(D)** Separate renal segments and distribution of PE 38:4, PI 36:4 and PI 36:1 lipids over each renal cluster.

Images were recorded in negative-ion mode at a pixel size of 10 µm. Scale bar = 150 µm. *The algorithm automatically detected eight histologic areas: Glomeruli (G,red), Proximal tubules I (PT I, garnet), Proximal tubules II (PT II, yellow), Cortical distal tubules (CDT, blue), Corticomedullary tubules (CMT, green), Medullary distal tubules II (MT II, purple) and interstitial structure (orange). Abreviations: H&E, hematoxylin and eosin.

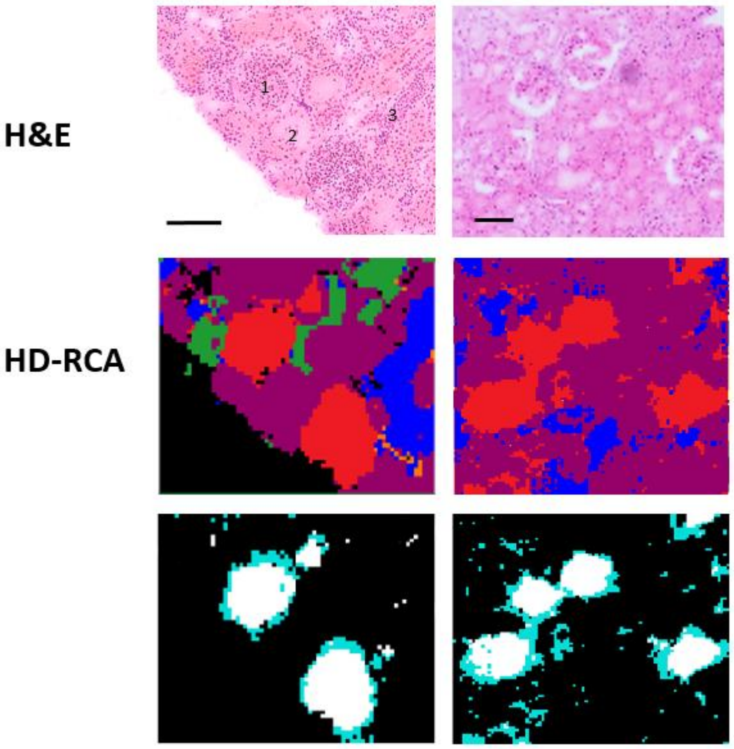

Figure S8. Separate renal segmentation of renal corpuscule IMS histological area in two cortical samples. Clustering results setting the number of segments in two represent the posible separation between glomerully (white) and Bowman's capsule (blue). Abbreviations; H&E, hematoxylin and eosin, HD-RCA, Segmentation algorithm used. Images were recorded in negative-ion mode at a pixel size of 10 µm. Scale bar = 150 µm.

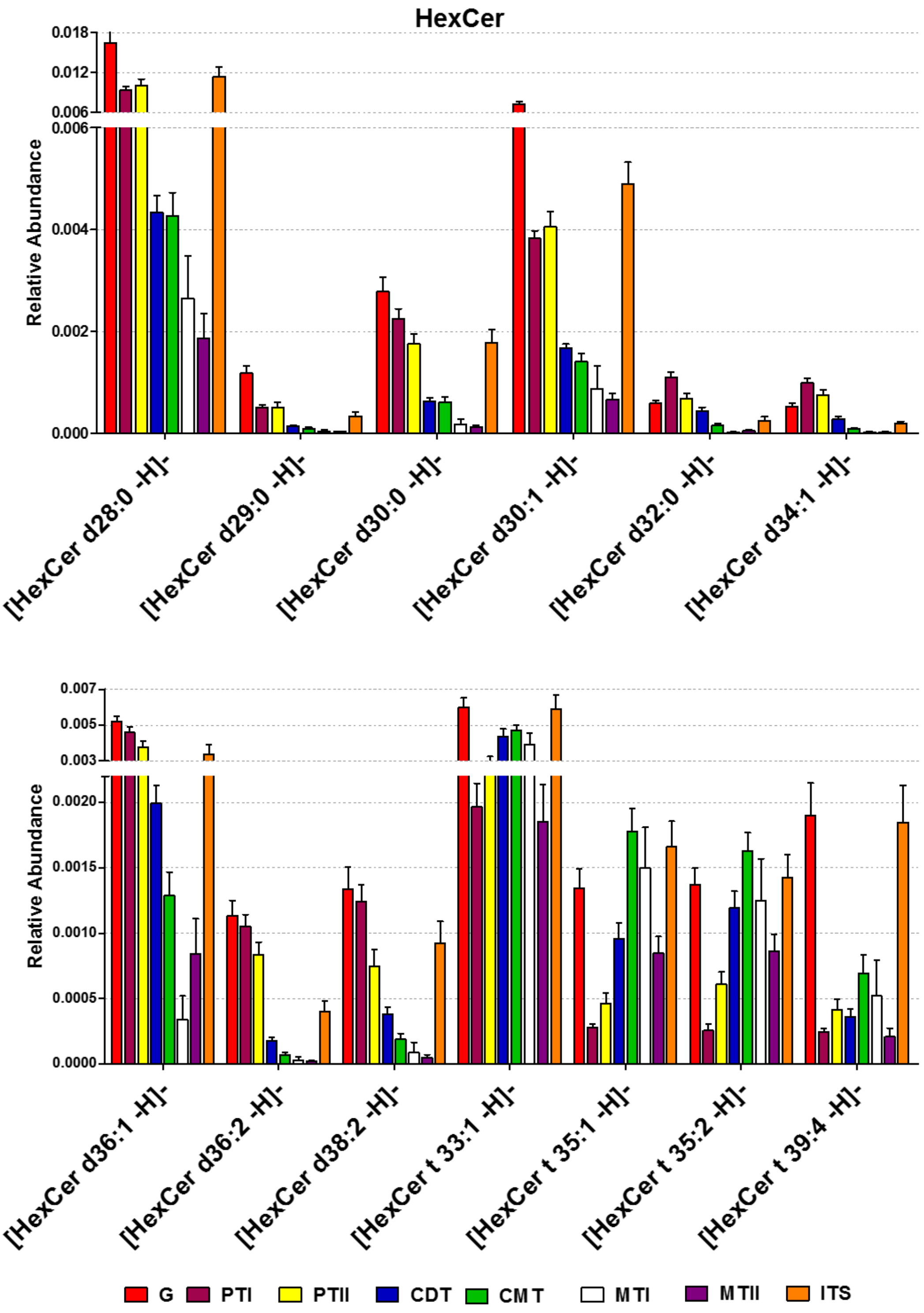

Figure S9. Bar charts of the relative abundance variation of the Hexo Ceramides lipid species in the eight histological found regions. Abbreviations; G, Glomeruli; PTI, Proximal tubule I; PTII, Proximal tubule II; CDT, Cortical distal tubule; CMT, Corticomedullar tubule; MTI, Medullary distal tubule I; MTII, Medullary distal tubule II; ITS, interstitial structure. Values are expressed as mean ± SEM. Statistical analysis reported in **Supplementary table S1.**

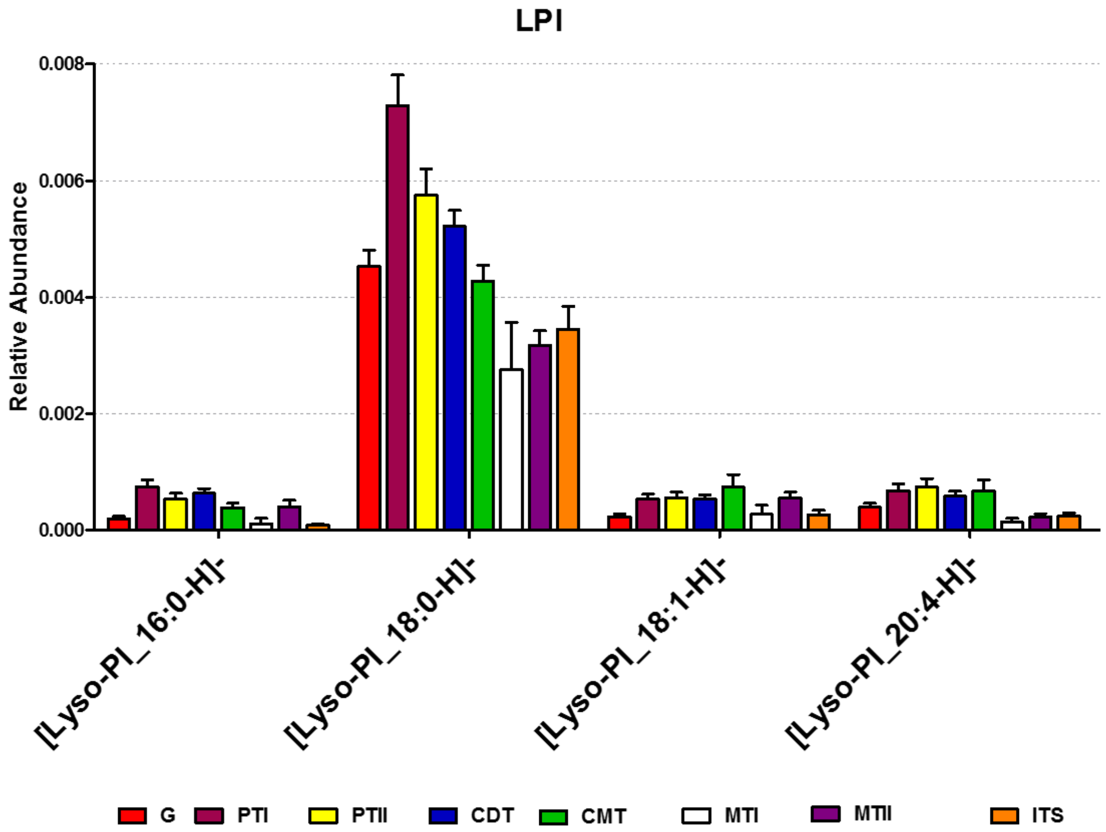

Figure S10. Bar charts of the relative abundance variation of the Lyso-Phosphatidylinositol lipid species in the eight histological found regions. Abbreviations; G, Glomeruli; PTI, Proximal tubule I; PTII, Proximal tubule II; CDT, Cortical distal tubule; CMT, Corticomedullar tubule; MTI, Medullary distal tubule I; MTII, Medullary distal tubule II; ITS, interstitial structure. Values are expressed as mean ± SEM. Statistical analysis reported in **Supplementary table S1.**

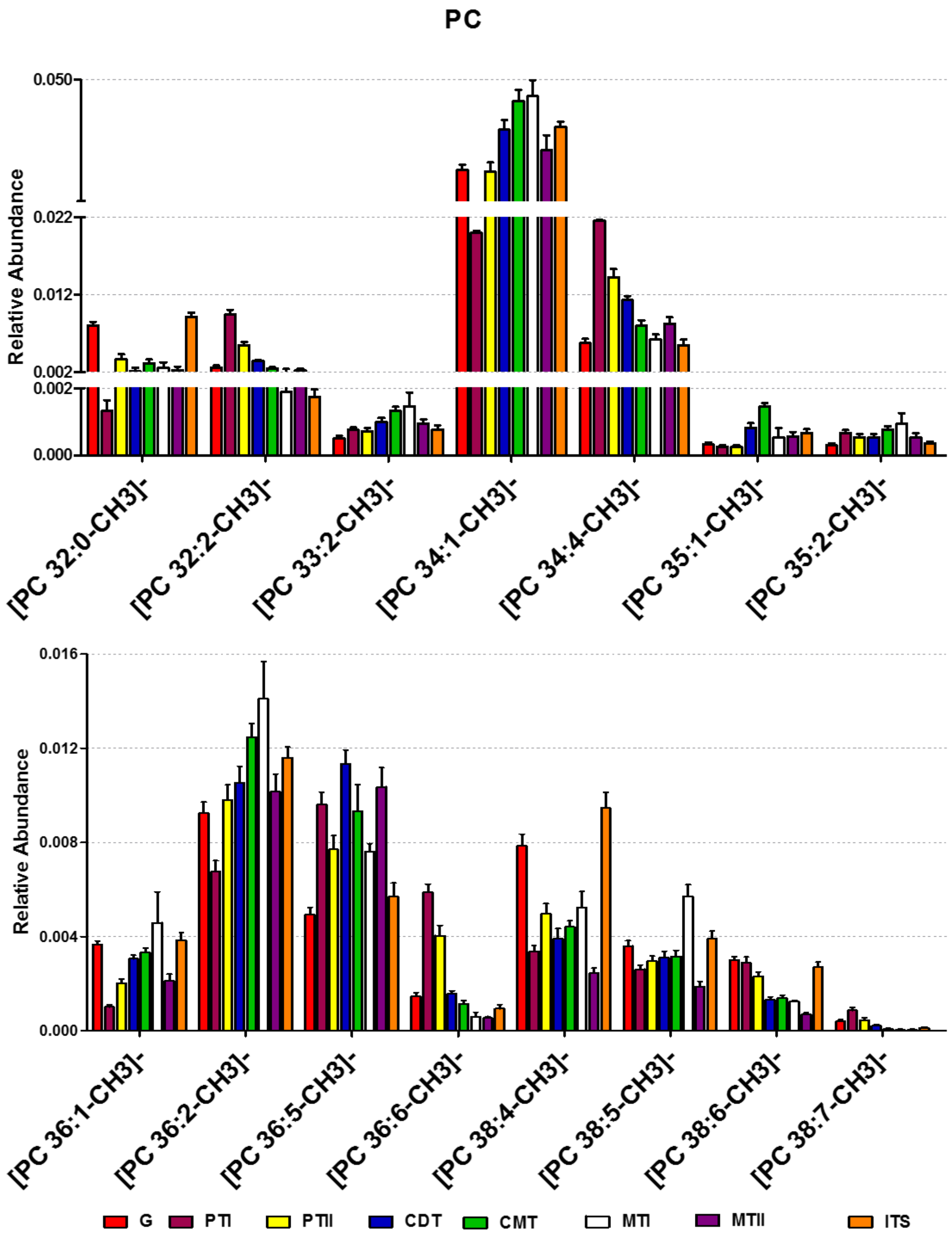

Figure S11. Bar charts of the relative abundance variation of the Phosphatidylcholine lipid species in the eight histological found regions. Abbreviations; G, Glomeruli; PTI, Proximal tubule I; PTII, Proximal tubule II; CDT, Cortical distal tubule; CMT, Corticomedullar tubule; MTI, Medullary distal tubule I; MTII, Medullary distal tubule II; ITS, interstitial structure. Values are expressed as mean ± SEM. Statistical analysis reported in **Supplementary table S1.**

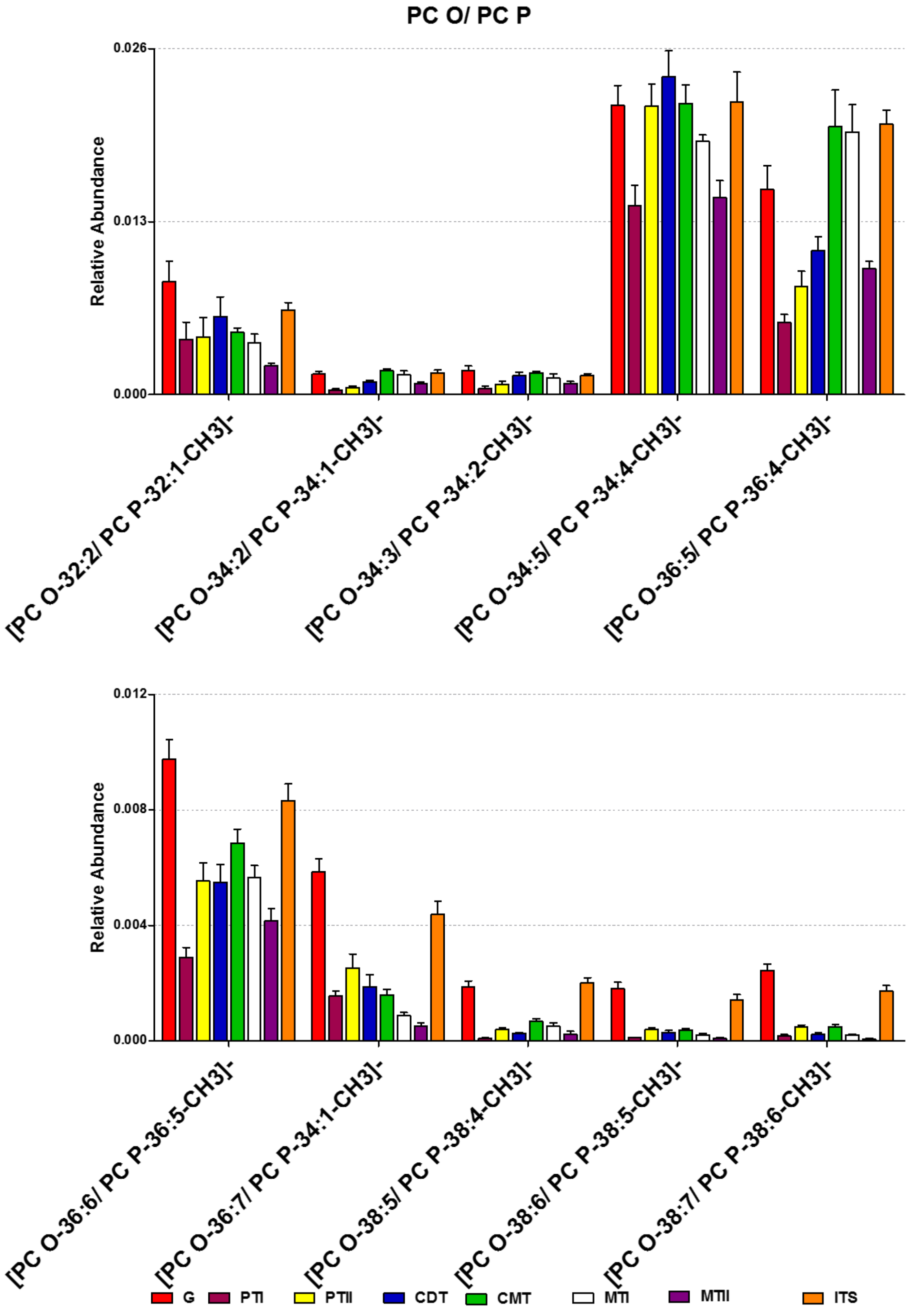

Figure S12. Bar charts of the relative abundance variation of the Phosphatidylcholine plasmalogen lipid species in the eight histological found regions. Abbreviations; G, Glomeruli; PTI, Proximal tubule I; PTII, Proximal tubule II; CDT, Cortical distal tubule; CMT, Corticomedullar tubule; MTI, Medullary distal tubule I; MTII, Medullary distal tubule II; ITS, interstitial structure. Values are expressed as mean ± SEM. Statistical analysis reported in **Supplementary table S1.**

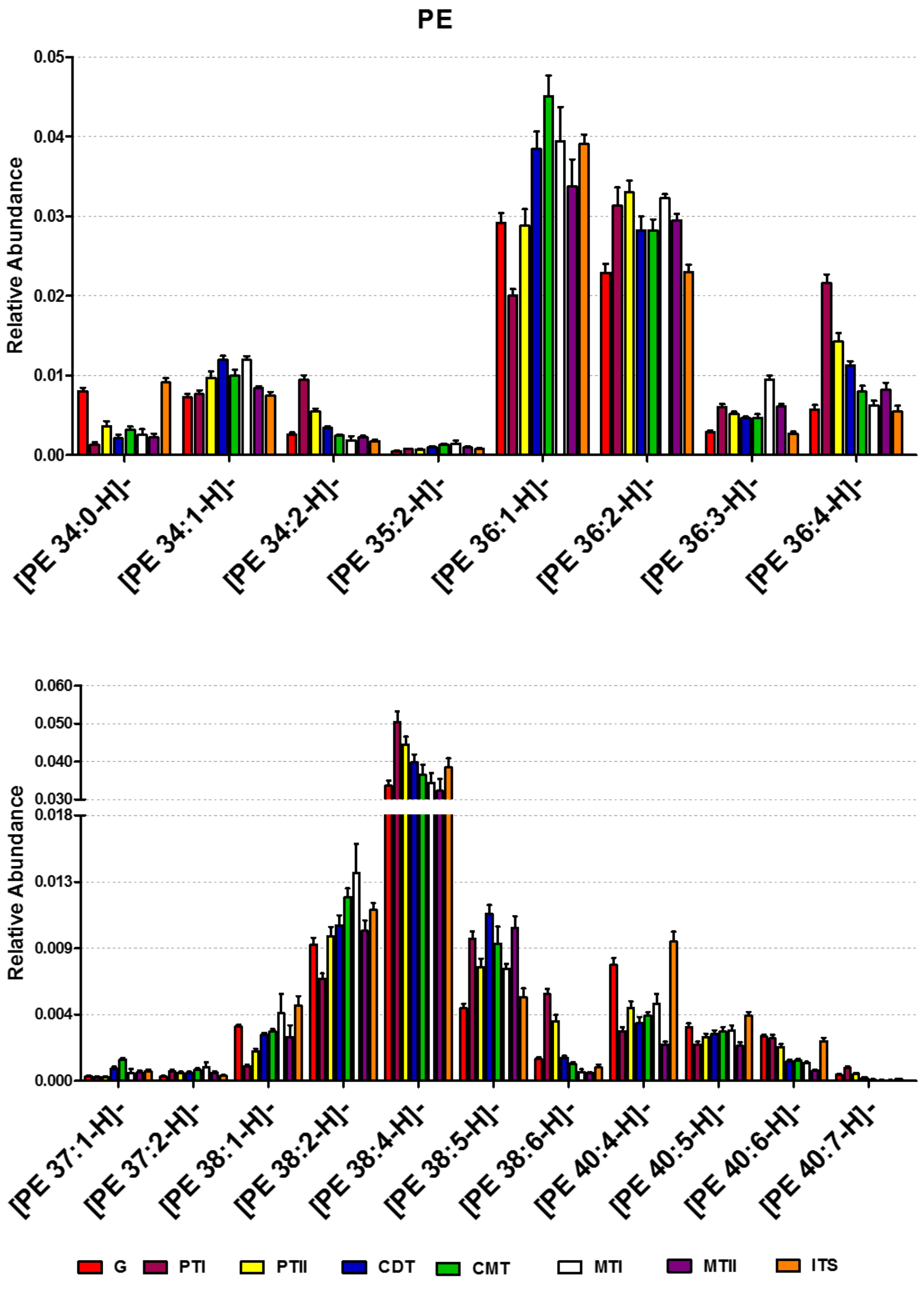

Figure S13. Bar charts of the relative abundance variation of the Phosphatidylethanoamine lipid species in the eight histological found regions. Abbreviations; G, Glomeruli; PTI, Proximal tubule I; PTII, Proximal tubule II; CDT, Cortical distal tubule; CMT, Corticomedullar tubule; MTI, Medullary distal tubule I; MTII, Medullary distal tubule II; ITS, interstitial structure. Values are expressed as mean ± SEM. Statistical analysis reported in **Supplementary table S1.**

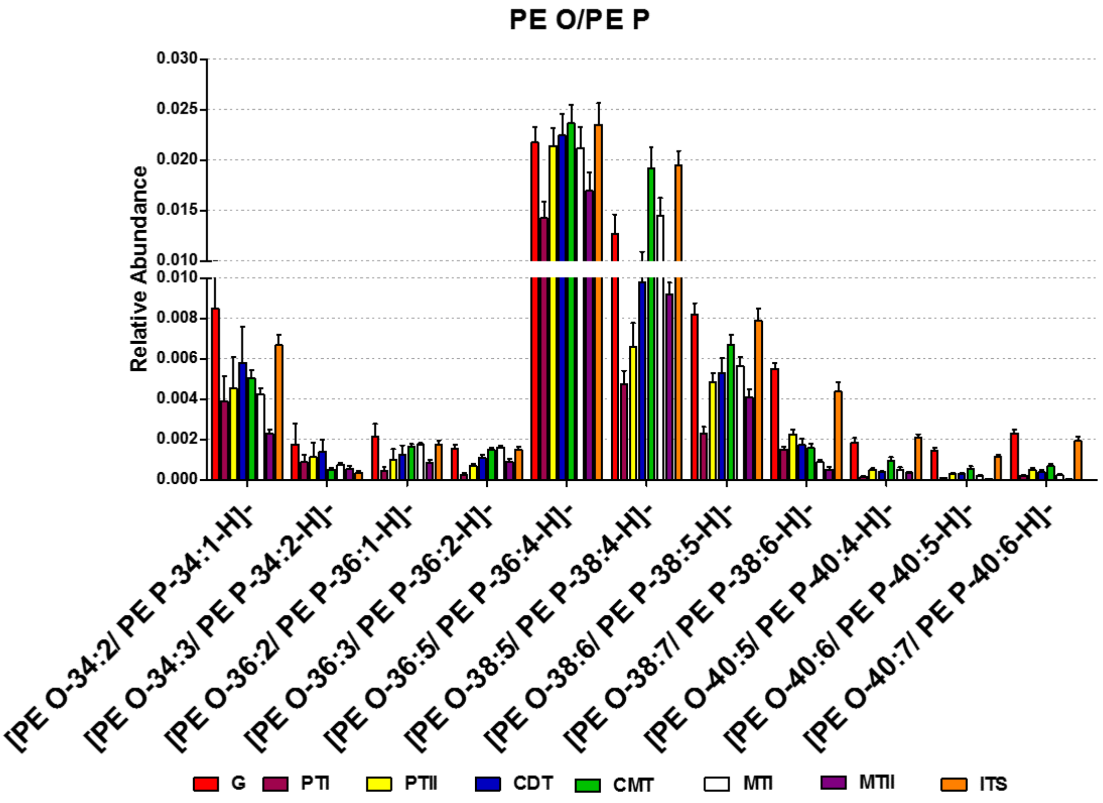

Figure S14. Bar charts of the relative abundance variation of the Phosphatidyletholamine plasmalogen lipid species in the eight histological found regions. Abbreviations; CPTI, Cortical proximal tubule I,; CPTII, cortical proximal tubule II; CDT, cortical distal tubule; G, Glomeruli; ITS, interstitial structure; MTI, Medullary distal tubule I; MTII, Medullary distal tubule II. Values are expressed as mean ± SEM. Statistical analysis reported in **Supplementary table S1.**

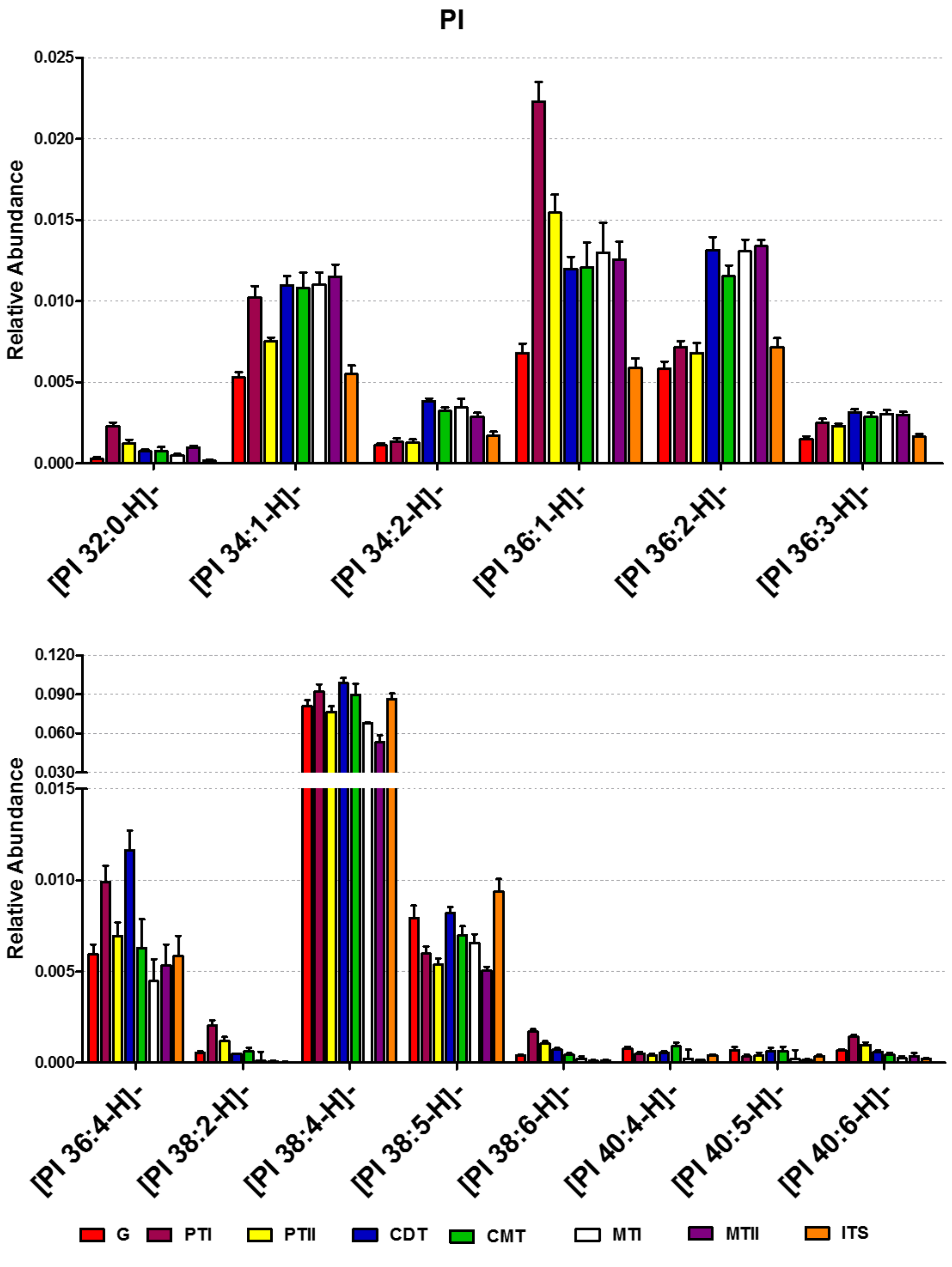

Figure S15. Bar charts of the relative abundance variation of the Lyso-Phosphatidylinositol and Phosphatidylinositol lipid species in the eight histological found regionsAbbreviations; G, Glomeruli; PTI, Proximal tubule I; PTII, Proximal tubule II; CDT, Cortical distal tubule; CMT, Corticomedullar tubule; MTI, Medullary distal tubule I; MTII, Medullary distal tubule II; ITS, interstitial structure. Values are expressed as mean ± SEM. Statistical analysis reported in **Supplementary table S1.**

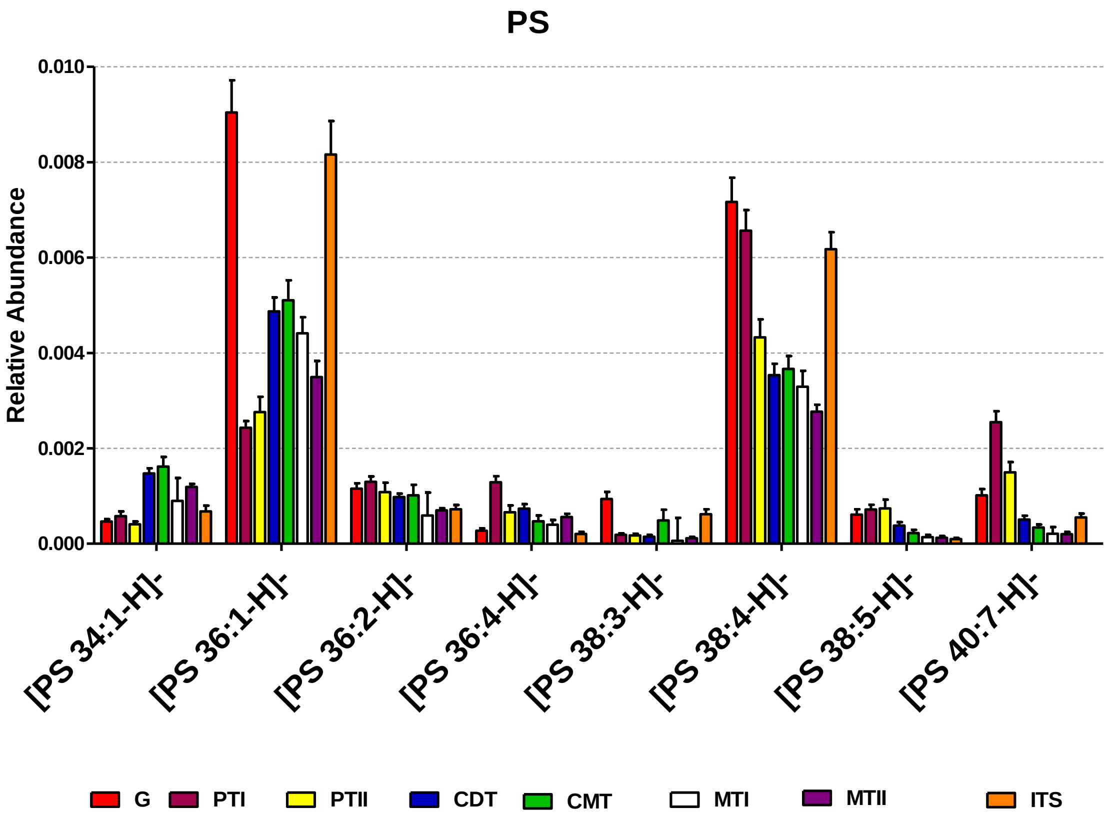

Figure S16. Bar charts of the relative abundance variation of the Phosphatidylserine lipid species in the eight histological found regions. Abbreviations; G, Glomeruli; PTI, Proximal tubule I; PTII, Proximal tubule II; CDT, Cortical distal tubule; CMT, Corticomedullar tubule; MTI, Medullary distal tubule I; MTII, Medullary distal tubule II; ITS, interstitial structure. Values are expressed as mean ± SEM. Statistical analysis reported in **Supplementary table S1.**

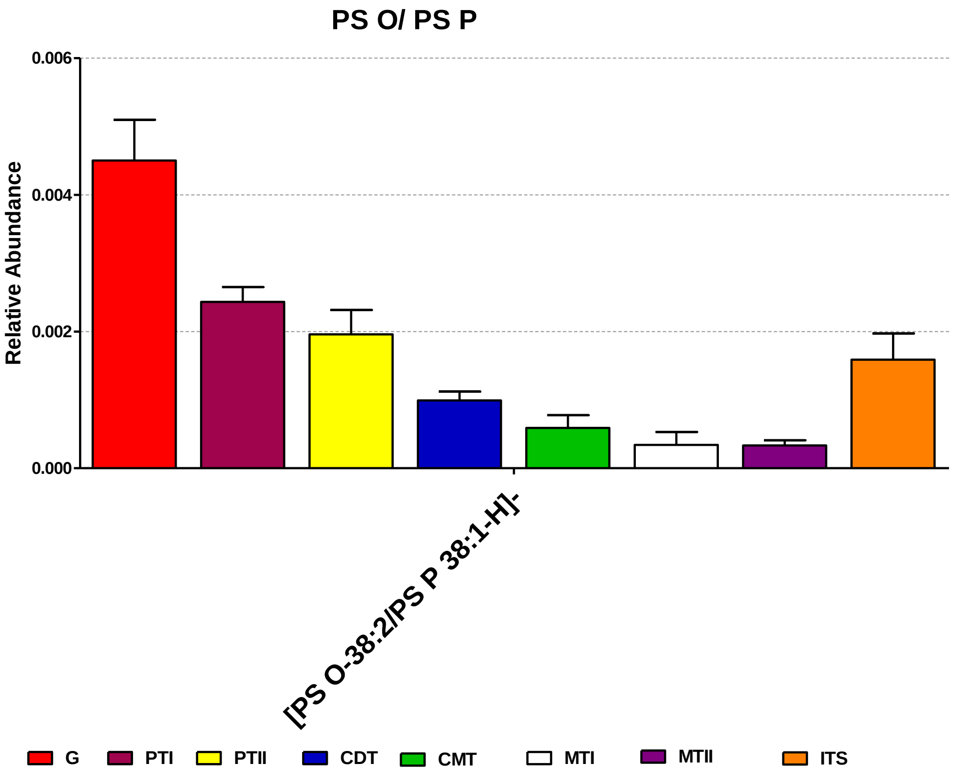

Figure S17. Bar charts of the relative abundance variation of the Phosphatidylserine plasmalogen lipid species in the eight histological found regions. Abbreviations; G, Glomeruli; PTI, Proximal tubule I; PTII, Proximal tubule II; CDT, Cortical distal tubule; CMT, Corticomedullar tubule; MTI, Medullary distal tubule I; MTII, Medullary distal tubule II; ITS, interstitial structure. Values are expressed as mean ± SEM. Statistical analysis reported in **Supplementary table S1.**

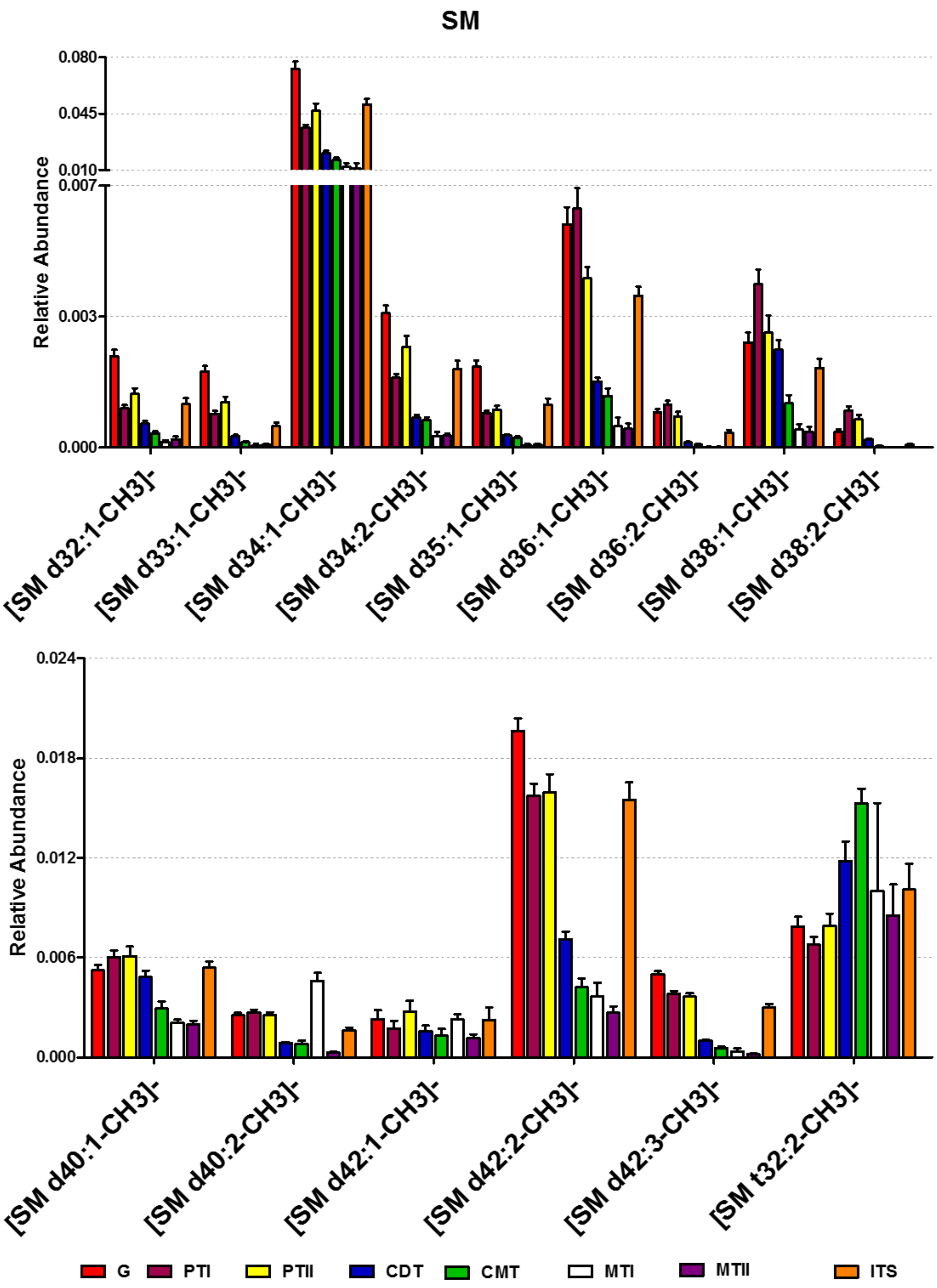

Figure S18. Bar charts of the relative abundance variation of the Sphingomieline lipid species in the eight histological found regions. Abbreviations; G, Glomeruli; PTI, Proximal tubule I; PTII, Proximal tubule II; CDT, Cortical distal tubule; CMT, Corticomedullar tubule; MTI, Medullary distal tubule I; MTII, Medullary distal tubule II; ITS, interstitial structure. Values are expressed as mean ± SEM. Statistical analysis reported in **Supplementary table S1**

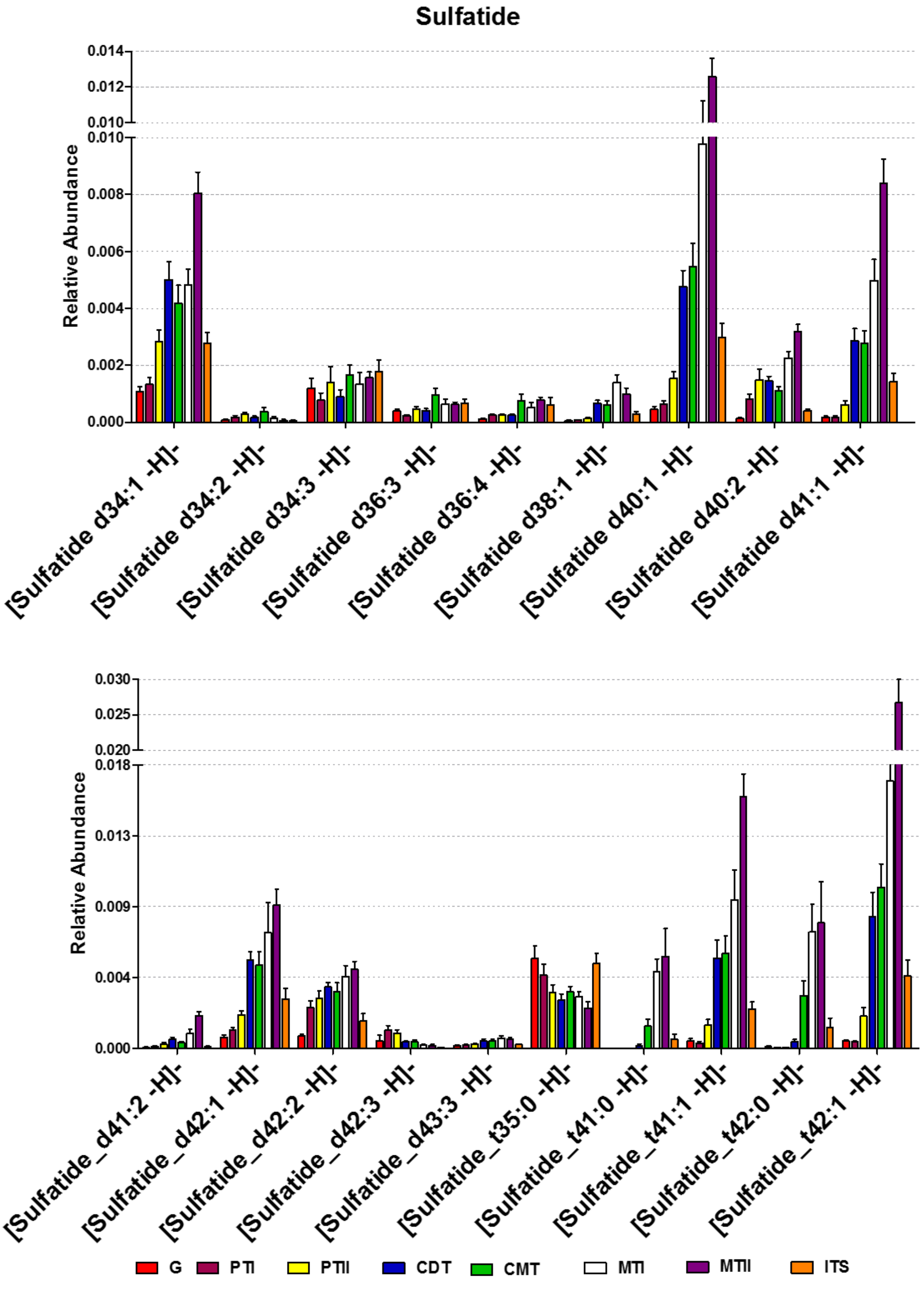

Figure S19. Bar charts of the relative abundance variation of the Sulfatide lipid species in the eight histological found regions. Abbreviations; G, Glomeruli; PTI, Proximal tubule I; PTII, Proximal tubule II; CDT, Cortical distal tubule; CMT, Corticomedullar tubule; MTI, Medullary distal tubule I; MTII, Medullary distal tubule II; ITS, interstitial structure. Values are expressed as mean ± SEM. Statistical analysis reported in **Supplementary table S1.**

A

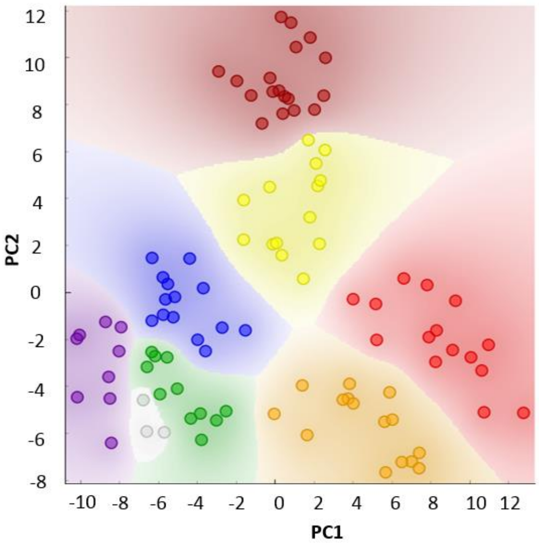

D

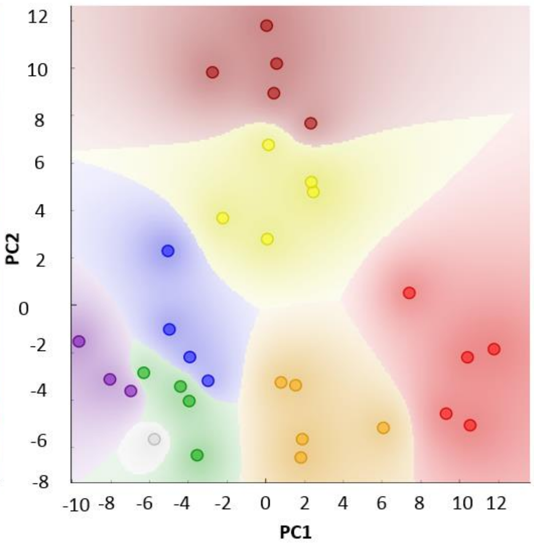

B

Scores

Method	AUC	CA	F1	Precision	Recall
SVM	0.988	0.949	0.937	0.929	0.949
Random Forest	1.000	1.000	1.000	1.000	1.000
Logistic Regression	1.000	1.000	1.000	1.000	1.000

E

Scores

Method	AUC	CA	F1	Precision	Recall
Logistic Regression	0.952	0.781	0.772	0.784	0.781
SVM	0.981	0.812	0.803	0.819	0.812
Random Forest	0.987	0.844	0.843	0.885	0.844

C

Actual \ Predicted	CDN	MTII	G	ITS	CMT	PTI	PTII	MTI	Σ
CDN	14	0	0	0	0	0	0	0	14
MTII	0	9	0	0	0	0	0	0	9
G	0	0	15	0	0	0	0	0	15
ITS	0	0	0	15	0	0	0	0	15
CMT	0	0	0	0	11	0	0	0	11
PTI	0	0	0	0	0	18	0	0	18
PTII	0	0	0	0	0	0	14	0	14
MTI	0	0	0	0	0	0	0	3	3
Σ	14	9	15	15	11	18	14	3	99

F

Actual \ Predicted	CDN	MTII	G	ITS	CMT	PTI	PTII	MTI	Σ
CDN	4	0	0	0	0	0	0	0	4
MTII	0	2	0	0	0	0	0	1	3
G	0	0	3	1	0	0	0	0	4
ITS	0	0	0	5	0	0	0	0	5
CMT	1	0	0	0	3	0	0	0	4
PTI	0	0	0	0	0	6	0	0	6
PTII	1	0	1	0	0	0	3	0	5
MTI	0	0	0	0	0	0	0	1	1
Σ	6	2	4	6	3	6	3	2	32

Figure S20. Statistical analysis of the lipid fingerprints from renal samples. The 131 different-enough clusters were divided randomly into discovery and validation group (75%-25% respectively). (**A**). Principal component analysis visually shows the separation tendencies of the eight histological areas found in the discovery group, based on their differential biomarkers expression. The analysis was carried out using the identified lipid species whose identities are reported in **Supplementary Table S1**. (**B**). Summary of the models' performance classifications. (**C**) Confusion matrix showing multi-class classification results by Random Forest. (**D**). Principal component analysis of the validation group using the biomarkers found in the training group. An almost perfect separation was obtained. (**E**). Summary of the performance of the classification methods. (**F**) Confusion matrix showing multi-class classification results by Random Forest.

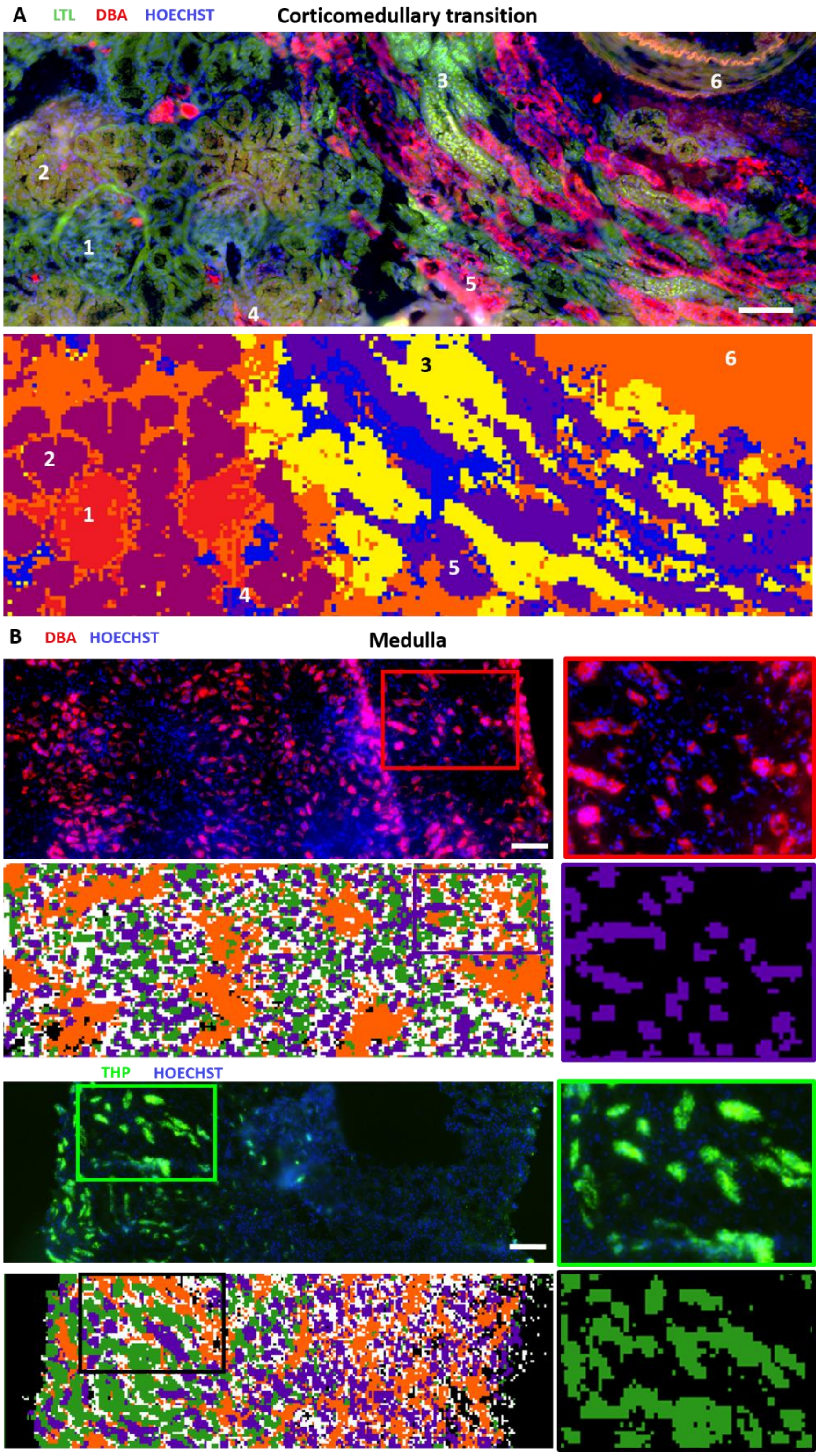

Figure S21. Comparison between the IF staining and the segmentation analysis of the IMS experiment carried out over the same histological section. A) Section of a cortical-medullary transition. Segment-specific tubular markers used the following: proximal tubule, *Lotus tetragonolobus* lectin (LTL), distal convoluted tubules and collecting ducts, *Dolichos biflorus* agglutinin (DBA). Numbers indicate: 1: glomerulus; 2: proximal tubule I (PTI), 3: proximal tubule II (PTII), 4: Cortical distal tubule (CDT), 5: Collecting duct (MTII) and 6: capillary; B) Section of a medullary biopsy. DBA-positive collecting ducts correlate with the purple segment, attributed to MT II based on its lipid signature. Scale bar = 150 µm

Supplementary Table S1. Statistical analysis of the lipid fingerprints

Lipid specie	Levene test	ANOVA	G vs PTI	G vs PTII	G vs CDT	G vs CMT	G vs MTI	G vs MTII	G vs ITS	PTI vs PTII	PTI vs CDT	PTI vs CMT	PTI vs MTI	PTI vs MTII	PTI vs ITS
[HexCer d28:0 -H]-	1.50E-04	1.45E-17	5.00E-03	3.70E-02	0.00E+00	0.00E+00	0.00E+00	0.00E+00	2.43E-01	9.77E-01	0.00E+00	0.00E+00	2.40E-02	0.00E+00	6.37E-01
[HexCer d29:0 -H]-	3.73E-09	5.55E-18	1.00E-03	5.00E-03	0.00E+00	0.00E+00	0.00E+00	0.00E+00	0.00E+00	1.00E+00	0.00E+00	0.00E+00	0.00E+00	0.00E+00	7.88E-01
[HexCer d30:0 -H]-	3.35E-06	1.50E-16	6.22E-01	9.10E-02	0.00E+00	0.00E+00	0.00E+00	0.00E+00	1.39E-01	8.10E-01	0.00E+00	0.00E+00	0.00E+00	0.00E+00	8.95E-01
[HexCer d30:1 -H]-	1.91E-06	1.10E-33	0.00E+00	0.00E+00	0.00E+00	0.00E+00	0.00E+00	0.00E+00	5.00E-03	9.73E-01	0.00E+00	0.00E+00	8.30E-02	0.00E+00	1.05E-01
[HexCer d32:0 -H]-	1.56E-04	3.18E-15	3.00E-03	9.90E-01	8.06E-01	0.00E+00	0.00E+00	0.00E+00	2.60E-02	9.20E-02	0.00E+00	0.00E+00	0.00E+00	0.00E+00	0.00E+00
[HexCer d34:1 -H]-	1.82E-07	6.92E-19	3.00E-03	5.74E-01	6.40E-02	0.00E+00	0.00E+00	0.00E+00	2.00E-03	5.67E-01	0.00E+00	0.00E+00	0.00E+00	0.00E+00	0.00E+00
[HexCer d36:1 -H]-	9.49E-03	1.14E-19	6.40E-01	3.10E-02	0.00E+00	0.00E+00	0.00E+00	0.00E+00	4.20E-02	7.42E-01	0.00E+00	0.00E+00	0.00E+00	0.00E+00	5.06E-01
[HexCer d36:2 -H]-	3.44E-06	1.95E-21	9.95E-01	4.91E-01	0.00E+00	0.00E+00	0.00E+00	0.00E+00	0.00E+00	8.16E-01	0.00E+00	0.00E+00	0.00E+00	0.00E+00	0.00E+00
[HexCer d38:2 -H]-	4.89E-06	1.23E-11	9.98E-01	1.38E-01	1.00E-03	0.00E+00	0.00E+00	0.00E+00	6.00E-01	2.27E-01	0.00E+00	0.00E+00	0.00E+00	0.00E+00	8.41E-01
[HexCer t35:1 -H]-	9.14E-03	1.65E-18	0.00E+00	0.00E+00	4.76E-01	5.55E-01	9.99E-01	2.09E-01	8.56E-01	8.10E-02	0.00E+00	0.00E+00	2.05E-01	9.00E-03	0.00E+00
[HexCer t35:2 -H]-	1.22E-01	9.12E-17	0.00E+00	0.00E+00	9.49E-01	8.12E-01	1.00E+00	1.73E-01	1.00E+00	5.10E-02	0.00E+00	0.00E+00	9.00E-03	8.00E-03	0.00E+00
[Lyso-PI 16:0-H]-	2.43E-06	4.39E-07	1.00E-03	8.80E-02	6.00E-03	9.00E-01	7.44E-01	8.66E-01	3.95E-01	7.78E-01	8.02E-01	7.40E-02	7.14E-01	1.30E-02	0.00E+00
[Lyso-PI 18:0-H]-	1.27E-01	4.66E-11	0.00E+00	1.98E-01	9.94E-01	9.97E-01	1.00E+00	3.41E-01	7.80E-01	6.20E-02	0.00E+00	0.00E+00	1.70E-01	0.00E+00	0.00E+00
[Lyso-PI 18:1-H]-	2.64E-08	3.07E-27	2.70E-02	9.70E-02	2.64E-01	5.04E-01	4.30E-02	1.12E-01	9.06E-01	1.00E+00	9.80E-01	9.87E-01	4.60E-02	1.00E+00	5.97E-01
[Lyso-PI 20:4-H]-	9.69E-06	7.71E-23	2.45E-01	3.40E-01	9.04E-01	8.80E-01	4.90E-02	9.99E-01	9.78E-01	1.00E+00	8.87E-01	1.00E+00	5.20E-02	9.90E-02	4.50E-02
[PC 32:0-CH3]-	2.63E-01	2.03E-27	0.00E+00	0.00E+00	0.00E+00	0.00E+00	1.00E-03	0.00E+00	7.32E-01	1.00E-03	6.50E-01	4.90E-02	9.13E-01	7.50E-01	0.00E+00
[PC 32:2-CH3]-	5.49E-11	2.47E-33	0.00E+00	0.00E+00	1.73E-01	1.00E+00	8.81E-01	9.43E-01	2.05E-01	0.00E+00	0.00E+00	0.00E+00	0.00E+00	0.00E+00	0.00E+00
[PC 33:2-CH3]-	6.19E-01	8.92E-06	4.85E-01	7.65E-01	1.40E-02	0.00E+00	1.00E-02	1.71E-01	5.76E-01	1.00E+00	7.00E-01	7.00E-03	1.47E-01	9.59E-01	1.00E+00
[PC 34:1-CH3]-	9.45E-05	8.43E-18	0.00E+00	1.00E+00	2.00E-02	1.00E-03	1.63E-01	8.96E-01	0.00E+00	1.00E-02	0.00E+00	0.00E+00	7.10E-02	4.10E-02	0.00E+00
[PC 34:4-CH3]-	1.21E-03	1.31E-31	0.00E+00	0.00E+00	0.00E+00	2.52E-01	9.97E-01	2.86E-01	1.00E+00	0.00E+00	0.00E+00	0.00E+00	0.00E+00	0.00E+00	0.00E+00
[PC 35:1-CH3]-	3.97E-03	1.15E-15	9.84E-01	9.84E-01	3.90E-02	0.00E+00	9.88E-01	6.56E-01	1.45E-01	1.00E+00	1.00E-02	0.00E+00	9.52E-01	3.50E-01	3.00E-02
[PC 35:2-CH3]-	1.67E-02	1.94E-03	2.00E-02	3.60E-01	3.27E-01	1.20E-02	6.12E-01	7.10E-01	9.95E-01	9.62E-01	9.57E-01	9.98E-01	9.77E-01	9.76E-01	6.00E-02
[PC 36:1-CH3]-	2.07E-03	5.43E-25	0.00E+00	0.00E+00	9.20E-02	8.04E-01	9.87E-01	7.00E-03	9.99E-01	0.00E+00	0.00E+00	0.00E+00	3.95E-01	3.30E-02	0.00E+00
[PC 36:2-CH3]-	3.91E-01	2.16E-10	2.20E-02	9.95E-01	7.09E-01	7.00E-03	3.20E-02	9.80E-01	7.40E-02	1.00E-03	0.00E+00	0.00E+00	0.00E+00	1.00E-02	0.00E+00
[PC 36:5-CH3]-	3.93E-04	5.71E-13	0.00E+00	5.00E-03	0.00E+00	3.70E-02	1.20E-02	1.00E-03	8.85E-01	1.30E-01	5.54E-01	1.00E+00	3.70E-02	9.99E-01	0.00E+00
[PC 36:6-CH3]-	5.83E-06	1.11E-32	0.00E+00	0.00E+00	1.00E+00	7.49E-01	1.08E-01	0.00E+00	2.21E-01	1.20E-02	0.00E+00	0.00E+00	0.00E+00	0.00E+00	0.00E+00
[PC 38:4-CH3]-	6.67E-02	8.09E-23	0.00E+00	0.00E+00	0.00E+00	0.00E+00	2.71E-01	0.00E+00	1.37E-01	2.00E-02	8.48E-01	4.38E-01	5.35E-01	9.80E-01	0.00E+00
[PC 38:5-CH3]-	5.02E-01	2.89E-08	2.10E-02	4.49E-01	7.83E-01	9.17E-01	1.60E-02	1.00E-03	9.80E-01	8.91E-01	6.44E-01	6.66E-01	0.00E+00	6.25E-01	1.00E-03
[PC 38:6-CH3]-	4.97E-04	1.26E-15	1.00E+00	1.02E-01	0.00E+00	0.00E+00	0.00E+00	0.00E+00	9.11E-01	6.22E-01	0.00E+00	0.00E+00	0.00E+00	0.00E+00	9.99E-01
[PC 38:7-CH3]-	1.72E-10	1.54E-10	4.50E-02	1.00E+00	4.13E-01	1.00E-02	6.00E-03	2.00E-03	3.80E-02	1.48E-01	1.00E-03	0.00E+00	0.00E+00	0.00E+00	0.00E+00
[PC O-32:2/ PC P-32:1-CH3]-	2.82E-01	8.17E-02													
[PC O-34:2/ PC P-34:1-CH3]-	3.13E-02	2.74E-13	0.00E+00	6.00E-03	3.08E-01	9.86E-01	1.00E+00	1.35E-01	1.00E+00	6.83E-01	1.00E-03	0.00E+00	2.26E-01	2.50E-02	0.00E+00
[PC O-34:3/ PC P-34:2-CH3]-	3.81E-01	4.18E-04	1.00E-03	4.60E-02	9.54E-01	1.00E+00	9.90E-01	3.41E-01	9.64E-01	9.10E-01	3.10E-02	1.50E-02	8.68E-01	9.36E-01	4.00E-02
[PC O-34:5/ PC P-34:4-CH3]-	2.37E-01	1.15E-04	1.00E-02	1.00E+00	9.82E-01	1.00E+00	9.99E-01	2.53E-01	1.00E+00	8.00E-03	0.00E+00	2.70E-02	9.27E-01	1.00E+00	1.00E-02
[PC O-36:5/ PC P-36:4-CH3]-	6.38E-05	3.28E-16	0.00E+00	3.20E-02	3.77E-01	8.26E-01	7.40E-01	6.90E-02	2.65E-01	1.61E-01	1.00E-03	2.00E-03	5.90E-02	0.00E+00	0.00E+00
[PC O-36:6/ PC P-36:5-CH3]-	4.30E-01	3.34E-15	0.00E+00	0.00E+00	0.00E+00	4.00E-03	6.60E-02	0.00E+00	3.16E-01	9.00E-03	1.00E-03	0.00E+00	5.44E-01	8.77E-01	0.00E+00
[PC O-36:7/ PC P-34:1-CH3]-	4.67E-02	4.82E-20	0.00E+00	0.00E+00	0.00E+00	0.00E+00	9.98E-01	0.00E+00	6.09E-01	5.54E-01	9.83E-01	1.00E+00	4.10E-02	1.00E-03	0.00E+00
[PC O-38:5/ PC P-38:4-CH3]-	5.58E-10	2.79E-25	0.00E+00	0.00E+00	0.00E+00	2.00E-03	0.00E+00	0.00E+00	1.00E+00	5.70E-02	1.10E-02	2.10E-02	8.23E-01	1.00E+00	0.00E+00
[PC O-38:6/ PC P-38:5-CH3]-	4.72E-13	3.75E-24	0.00E+00	0.00E+00	0.00E+00	0.00E+00	1.18E-01	0.00E+00	1.00E+00	4.60E-02	6.00E-02	8.00E-03	7.88E-01	9.04E-01	0.00E+00
[PC O-38:7/ PC P-38:6-CH3]-	4.64E-17	4.61E-33	0.00E+00	0.00E+00	0.00E+00	0.00E+00	0.00E+00	0.00E+00	8.40E-02	4.80E-02	6.50E-01	5.20E-02	1.00E+00	2.52E-01	0.00E+00
[PE 34:0-H]-	2.63E-01	2.03E-27	0.00E+00	0.00E+00	0.00E+00	0.00E+00	1.00E-03	0.00E+00	7.32E-01	1.00E-03	6.50E-01	4.90E-02	9.13E-01	7.50E-01	0.00E+00
[PE 34:1-H]-	4.58E-04	1.81E-08	9.94E-01	1.73E-01	0.00E+00	8.90E-02	3.00E-03	3.95E-01	1.00E+00	4.57E-01	0.00E+00	2.73E-01	5.00E-03	9.41E-01	1.00E+00
[PE 34:2-H]-	5.49E-11	2.47E-33	0.00E+00	0.00E+00	1.73E-01	1.00E+00	8.81E-01	9.43E-01	2.05E-01	0.00E+00	0.00E+00	0.00E+00	0.00E+00	0.00E+00	0.00E+00
[PE 35:2-H]-	6.19E-01	8.92E-06	4.85E-01	7.65E-01	1.40E-02	0.00E+00	1.00E-02	1.71E-01	5.76E-01	1.00E+00	7.00E-01	7.00E-03	1.47E-01	9.59E-01	1.00E+00
[PE 36:1-H]-	1.06E-04	4.57E-17	0.00E+00	1.00E+00	2.00E-02	1.00E-03	4.97E-01	8.96E-01	0.00E+00	1.00E-02	0.00E+00	0.00E+00	1.71E-01	4.10E-02	0.00E+00
[PE 36:2-H]-	8.35E-05	1.72E-05	2.30E-02	0.00E+00	2.05E-01	8.70E-02	0.00E+00	2.00E-03	1.00E+00	1.00E+00	8.94E-01	8.51E-01	1.00E+00	9.66E-01	1.90E-02
[PE 36:3-H]-	3.99E-02	3.54E-20	0.00E+00	0.00E+00	0.00E+00	3.00E-02	1.00E-02	0.00E+00	9.98E-01	2.69E-01	7.00E-03	1.39E-01	3.40E-02	1.00E+00	0.00E+00
[PE 36:4-H]-	1.21E-03	1.31E-31	0.00E+00	0.00E+00	0.00E+00	2.52E-01	9.97E-01	2.86E-01	1.00E+00	0.00E+00	0.00E+00	0.00E+00	0.00E+00	0.00E+00	0.00E+00
[PE 37:1-H]-	3.97E-03	1.15E-15	9.84E-01	9.84E-01	3.90E-02	0.00E+00	9.88E-01	6.56E-01	1.45E-01	1.00E+00	1.00E-02	0.00E+00	9.52E-01	3.50E-01	3.00E-02
[PE 37:2-H]-	1.67E-02	1.94E-03	2.00E-02	3.60E-01	3.27E-01	1.20E-02	6.12E-01	7.10E-01	9.95E-01	9.62E-01	9.57E-01	9.98E-01	9.77E-01	9.76E-01	6.00E-02
[PE 38:1-H]-	2.14E-07	1.38E-17	0.00E+00	0.00E+00	1.61E-01	8.04E-01	9.87E-01	9.83E-01	2.40E-01	0.00E+00	0.00E+00	0.00E+00	3.95E-01	2.85E-01	0.00E+00
[PE 38:2-H]-	2.58E-01	2.14E-10	3.30E-02	9.94E-01	6.78E-01	5.00E-03	2.40E-02	9.77E-01	5.90E-02	2.00E-03	0.00E+00	0.00E+00	0.00E+00	1.20E-02	0.00E+00
[PE 38:4-H]-	6.24E-02	2.60E-07	0.00E+00	1.20E-02	5.01E-01	9.89E-01	1.00E+00	1.00E+00	7.82E-01	3.25E-01	6.00E-03	1.00E-03	1.03E-01	0.00E+00	2.00E-03
[PE 38:5-H]-	3.93E-04	5.71E-13	0.00E+00	5.00E-03	0.00E+00	3.70E-02	1.20E-02	1.00E-03	8.85E-01	1.30E-01	5.54E-01	1.00E+00	3.70E-02	9.99E-01	0.00E+00
[PE 38:6-H]-	5.83E-06	1.11E-32	0.00E+00	0.00E+00	1.00E+00	7.49E-01	1.08E-01	0.00E+00	2.21E-01	1.20E-02	0.00E+00	0.00E+00	0.00E+00	0.00E+00	0.00E+00
[PE 40:4-H]-	6.67E-02	8.09E-23	0.00E+00	0.00E+00	0.00E+00	0.00E+00	2.71E-01	0.00E+00	1.37E-01	2.00E-02	8.48E-01	4.38E-01	5.35E-01	9.80E-01	0.00E+00
[PE 40:5-H]-	7.85E-01	4.49E-07	3.00E-03	3.97E-01	8.20E-01	9.90E-01	1.00E+00	4.10E-02	3.55E-01	5.79E-01	2.25E-01	1.33E-01	7.14E-01	1.00E+00	0.00E+00
[PE 40:6-H]-	4.97E-04	1.26E-15	1.00E+00	1.02E-01	0.00E+00	0.00E+00	0.00E+00	0.00E+00	9.11E-01	6.22E-01	0.00E+00	0.00E+00	0.00E+00	0.00E+00	9.99E-01
[PE 40:7-H]-	1.70E-10	1.56E-10	4.50E-02	1.00E+00	4.13E-01	1.00E-02	6.00E-03	2.00E-03	3.80E-02	1.48E-01	1.00E-03	0.00E+00	0.00E+00	0.00E+00	0.00E+00
[PE O-34:2/ PE P-34:1-H]-	2.82E-01	8.17E-02													
[PE O-34:3/ PE P-34:2-H]-	3.26E-02	7.73E-01													
[PE O-36:2/ PE P-36:1-H]-	2.09E-01	5.17E-02													
[PE O-36:3/ PE P-36:2-H]-	1.87E-01	7.54E-12	0.00E+00	0.00E+00	5.82E-01	1.00E+00	9.94E-01	9.80E-02	9.99E-01	3.20E-01	0.00E+00	0.00E+00	1.10E-01	1.44E-01	0.00E+00
[PE O-36:5/ PE P-36:4-H]-	2.35E-01	1.21E-04	1.00E-02	1.00E+00	9.86E-01	1.00E+00	9.99E-01	2.49E-01	1.00E+00	8.00E-03	0.00E+00	2.70E-02	9.27E-01	1.00E+00	1.00E-02
[PE O-38:5/ PE P-38:4-H]-	2.56E-07	6.00E-12	1.30E-02	1.17E-01	9.97E-01	5.36E-01	9.98E-01	4.08E-01	3.56E-01	9.33E-01	1.00E-03	4.00E-03	0.00E+00	3.00E-03	0.00E+00
[PE O-38:6/ PE P-38:5-H]-	4.95E-02	7.47E-15	0.00E+00	1.00E-03	9.50E-02	2.78E-01	1.00E+00	0.00E+00	9.74E-01	1.00E-03	1.00E-03	0.00E+00	4.00E-03	4.40E-02	0.00E+00
[PE O-38:7/ PE P-38:6-H]-	4.60E-03	1.51E-29	0.00E+00	0.00E+00	0.00E+00	0.00E+00	1.00E+00	0.00E+00	8.02E-01	2.79E-01	9.73E-01	9.98E-01	4.20E-02	1.00E-03	0.00E+00
[PE O-40:5/ PE P-40:4-H]-	3.99E-08	7.02E-32	0.00E+00	0.00E+00	0.00E+00	2.33E-01	8.20E-02	0.00E+00	9.79E-01	4.50E-02	3.10E-02	1.70E-02	4.80E-02	2.58E-01	0.00E+00
[PE O-40:6/ PE P-40:5-H]-	2.26E-07	4.56E-33	0.00E+00	0.00E+00	0.00E+00	7.10E-02	9.50E-02	0.00E+00	9.85E-01	2.88E-01	6.10E-02	1.18E-01	5.80E-02	1.00E+00	0.00E+00
[PE O-40:7/ PE P-40:6-H]-	5.51E-10	4.78E-36	0.00E+00	0.00E+00	0.00E+00	0.00E+00	2.18E-01	0.00E+00	9.91E-01	3.89E-01	3.07E-01	7.50E-02	6.10E-02	1.05E-01	0.00E+00
[PG40:7-H]-	2.16E-16	1.32E-40	4.35E-01	8.59E-01	9.99E-01	3.53E-01	5.10E-02	7.90E-01	9.98E-01	9.42E-01	7.83E-01	6.58E-01	5.20E-02	9.79E-01	2.90E-01
[PI 32:0-H]-	4.91E-09	3.24E-14	0.00E+00	2.60E-02	6.10E-02	6.20E-01	7.66E-01	4.00E-03	9.03E-01	3.50E-02	0.00E+00	2.00E-03	0.00E+00	0.00E+00	0.00E+00
[PI 34:1-H]-	2.68E-04	3.14E-16	0.00E+00	0.00E+00	0.00E+00	1.00E-03	4.20E-02	0.00E+00	1.00E+00	9.00E-03	1.00E+00	1.00E+00	9.99E-01	9.75E-01	0.00E+00
[PI 34:2-H]-	1.52E-02	1.14E-19	9.94E-01	9.99E-01	0.00E+00	0.00E+00	1.85E-01	0.00E+00	4.07E-01	1.00E+00	0.00E+00	0.00E+00	2.07E-01	2.00E-03	9.59E-01
[PI 36:1-H]-	1.63E-03	1.52E-24	0.00E+00	0.00E+00	0.00E+00	8.60E-02	2.94E-01	8.00E-03	9.24E-01	1.00E-03	0.00E+00	0.00E+00	7.40E-02	0.00E+00	0.00E+00
[PI 36:2-H]-	1.20E-02	5.45E-21	2.67E-01	8.87E-01	0.00E+00	0.00E+00	1.00E-02	0.00E+00	5.10E-01	9.98E-01	0.00E+00	1.00E-03	2.90E-02	0.00E+00	1.00E+00
[PI 36:3-H]-	3.27E-01	3.19E-17	5.80E-02	2.00E-02	0.00E+00	0.00E+00	0.00E+00	3.30E-02	9.79E-01	1.00E+00	2.56E-01	4.00E-02	0.00E+00	9.81E-01	5.26E-01
[PI 36:4-H]-	6.80E-02	8.36E-06	2.70E-02	9.94E-01	1.00E-03	1.00E+00	4.64E-01	1.00E+00	1.00E+00	1.75E-01	9.11E-01	1.33E-01	1.00E+00	6.50E-02	2.80E-02
[PI 38:2-H]-	9.31E-07	1.02E-17	0.00E+00	2.05E-01	9.95E-01	1.00E+00	6.60E-02	2.00E-03	1.00E-03	2.18E-01	0.00E+00	3.00E-03	1.60E-01	0.00E+00	0.00E+00
[PI 38:4-H]-	1.73E-02	3.44E-05	8.30E-01	9.96E-01	1.05E-01	9.85E-01	3.50E-02	1.80E-02	9.85E-01	4.20E-01	9.69E-01	1.00E+00	2.00E-03	1.00E-03	9.93E-01
[PI 38:5-H]-	3.19E-03	8.92E-10	1.77E-01	3.50E-02	1.00E+00	9.40E-01	6.08E-01	9.00E-03	7.33E-01	9.73E-01	2.00E-03	6.76E-01	1.80E-02	5.72E-01	1.00E-03
[PI 38:6-H]-	1.86E-06	1.95E-22	0.00E+00	5.00E-03	4.50E-02	1.00E+00	9.19E-01	2.00E-03	3.00E-03	1.60E-02	0.00E+00	0.00E+00	0.00E+00	0.00E+00	0.00E+00
[PI 40:4-H]-	1.57E-03	8.14E-27	5.74E-01	2.49E-01	7.38E-01	9.99E-01	5.70E-02	1.00E-03	1.24E-01	9.98E-01	1.00E+00	5.98E-01	5.20E-02	2.60E-02	9.91E-01
[PI 40:5-H]-	2.93E-01	1.80E-15	5.49E-01	8.29E-01	1.00E+00	1.00E+00	0.00E+00	5.03E-01	7.84E-01	1.00E+00	7.69E-01	7.96E-01	0.00E+00	9.99E-01	1.00E+00
[PI 40:6-H]-	2.07E-04	5.27E-15	0.00E+00	3.95E-01	9.97E-01	7.30E-01	3.15E-01	6.29E-01	0.00E+00	1.48E-01	0.00E+00	0.00E+00	4.00E-03	0.00E+00	0.00E+00
[PS 34:1-H]-	5.56E-05	7.60E-36	9.56E-01	9.96E-01	0.00E+00	1.00E-03	4.30E-02	0.00E+00	5.93E-01	7.79E-01	0.00E+00	4.00E-03	4.10E-02	0.00E+00	9.97E-01
[PS 36:1-H]-	2.68E-08	1.38E-26	0.00E+00	0.00E+00	0.00E+00	1.00E-03	0.00E+00	0.00E+00	9.77E-01	2.43E-01	0.00E+00	0.00E+00	1.11E-01	1.70E-02	0.00E+00
[PS 36:2-H]-	1.72E-08	1.33E-16	9.85E-01	1.00E+00	8.97E-01	9.99E-01	6.20E-02	2.40E-02	6.70E-02	9.79E-01	2.91E-01	9.36E-01	6.80E-02	1.00E-03	4.00E-03
[PS 36:4-H]-	2.09E-07	9.41E-12	0.00E+00	2.11E-01	4.00E-03	7.70E-01	4.79E-01	4.40E-02	9.62E-01	2.00E-02	1.10E-02	1.00E-03	6.00E-03	0.00E+00	0.00E+00
[PS 38:3-H]-	1.44E-13	6.68E-31	1.00E-03	2.00E-03	1.00E-03	7.08E-01	6.30E-02	1.00E-03	6.26E-01	9.72E-01	9.99E-01	7.28E-01	5.30E-02	1.00E+00	1.00E-03
[PS 38:4-H]-	4.74E-04	7.22E-14	9.39E-01	2.00E-03	0.00E+00	0.00E+00	1.30E-02	0.00E+00	7.09E-01	1.80E-02	0.00E+00	0.00E+00	8.20E-02	0.00E+00	1.00E+00
[PS 38:5-H]-	1.63E-07	4.14E-05	9.93E-01	9.98E-01	7.10E-01	1.11E-01	3.80E-02	1.20E-02	6.00E-03	1.00E+00	1.19E-01	4.00E-03	1.00E-03	0.00E+00	0.00E+00
[PS 40:7-H]-	1.56E-06	2.22E-20	0.00E+00	5.46E-01	4.20E-02	2.00E-03	9.60E-02	0.00E+00	6.80E-02	2.10E-02	0.00E+00	0.00E+00	0.00E+00	0.00E+00	0.00E+00
[PS O-38:2/ PS P-38:1-H]-	4.92E-09	6.29E-13	3.90E-02	1.90E-02	0.00E+00	0.00E+00	0.00E+00	0.00E+00	5.00E-03	9.88E-01	0.00E+00	0.00E+00	1.00E-03	0.00E+00	6.14E-01
[SM d32:1-CH3]-	4.13E-05	4.87E-24	1.41E-06	1.63E-03	1.71E-08	2.06E-09	8.01E-10	3.54E-10	2.69E-05	3.57E-01	1.32E-02	3.83E-06	1.94E-06	1.47E-06	9.94E-01
[SM d33:1-CH3]-	4.88E-06	2.68E-27	4.85E-06	6.04E-03	7.52E-09	1.35E-09	9.60E-10	7.63E-10	4.87E-08	5.28E-01	1.81E-06	1.38E-08	4.36E-08	3.16E-09	1.31E-01
[SM d34:1-CH3]-	4.20E-05	8.68E-26	3.39E-06	7.19E-03	1.07E-08	2.69E-09	3.01E-08	9.44E-10	1.00E-02	3.11E-01	3.39E-05	3.36E-07	5.11E-03	2.12E-04	3.53E-03
[SM d34:2-CH3]-	3.32E-06	6.55E-25	5.95E-07	2.32E-01	2.27E-10	8.40E-11	1.40E-09	2.12E-11	1.49E-04	1.46E-01	1.36E-08	2.74E-08	4.03E-04	3.62E-12	8.58E-01
[SM d35:1-CH3]-	3.09E-07	1.35E-26	6.95E-06	7.30E-05	3.30E-08	1.36E-08	4.18E-09	5.16E-09	1.50E-03	9.46E-01	8.55E-08	6.00E-08	1.79E-06	2.45E-11	6.19E-01
[SM d36:1-CH3]-	5.66E-10	1.19E-23	1.00E+00	1.72E-01	4.10E-07	4.62E-08	1.18E-07	3.76E-09	1.48E-02	1.35E-01	8.08E-07	1.35E-07	6.02E-08	7.40E-09	1.62E-02
[SM d36:2-CH3]-	1.19E-07	5.22E-19	8.92E-01	9.97E-01	4.20E-07	1.40E-07	5.91E-08	7.41E-08	4.09E-04	6.72E-01	1.87E-07	6.80E-08	2.64E-08	2.99E-08	5.85E-05
[SM d38:1-CH3]-	1.57E-03	3.35E-11	3.85E-02	1.00E+00	9.99E-01	1.25E-03	2.00E-05	6.83E-07	5.09E-01	3.78E-01	1.18E-02	1.30E-06	7.16E-08	1.46E-08	4.17E-04
[SM d38:2-CH3]-	1.55E-11	6.86E-17	8.90E-04	3.51E-01	1.71E-01	6.34E-04	2.17E-04	2.41E-04	2.51E-03	7.65E-01	2.05E-06	9.40E-08	5.32E-08	5.65E-08	1.41E-07
[SM d40:1-CH3]-	4.00E-01	1.89E-09	8.10E-01	7.97E-01	9.97E-01	1.25E-02	9.42E-02	4.42E-04	1.00E+00	1.00E+00	3.47E-01	5.08E-05	9.65E-03	1.64E-06	9.33E-01
[SM d40:2-CH3]-	2.79E-02	6.81E-26	9.94E-01	1.00E+00	6.67E-09	2.26E-05	1.86E-01	3.75E-11	2.73E-03	9.98E-01	1.61E-08	6.18E-06	2.15E-01	6.97E-11	1.13E-03
[SM d42:1-CH3]-	2.82E-09	4.98E-01													
[SM d42:2-CH3]-	1.93E-02	1.28E-32	8.40E-03	1.38E-01	1.25E-12	9.37E-13	5.95E-05	1.09E-12	2.84E-02	1.00E+00	3.62E-10	1.53E-12	4.43E-04	9.66E-13	1.00E+00
[SM d42:3-CH3]-	6.21E-04	5.36E-45	7.12E-04	6.30E-04	1.06E-12	1.03E-12	1.59E-06	1.08E-12	3.78E-07	1.00E+00	1.04E-12	9.41E-13	3.79E-05	1.06E-12	8.13E-02
[Sulfatide d34:1 -H]-	3.79E-04	5.95E-14	1.00E+00	1.66E-02	1.37E-04	2.47E-02	4.11E-01	1.98E-04	1.63E-03	3.73E-02	2.31E-04	3.33E-02	4.45E-01	1.80E-04	6.21E-03
[Sulfatide d34:2 -H]-	1.69E-10	1.01E-37	9.50E-01	6.21E-02	6.32E-01	5.51E-01	5.77E-02	1.00E+00	5.98E-01	4.66E-01	1.00E+00	7.54E-01	5.73E-02	9.84E-01	6.06E-01
[Sulfatide d34:3 -H]-	2.78E-02	3.66E-07	9.79E-01	1.00E+00	9.87E-01	8.10E-01	3.06E-03	4.22E-01	6.30E-01	9.97E-01	1.00E+00	2.65E-01	6.71E-03	3.53E-03	6.10E-02
[Sulfatide d36:3 -H]-	4.82E-09	1.67E-29	6.24E-01	1.00E+00	1.00E+00	3.04E-01	3.69E-02	9.86E-03	2.31E-01	8.16E-01	8.41E-01	1.23E-01	3.69E-02	1.29E-05	1.41E-02
[Sulfatide d36:4 -H]-	3.03E-10	5.59E-27	4.26E-02	1.24E-01	5.57E-01	2.10E-01	3.61E-02	4.40E-08	1.78E-01	1.00E+00	9.81E-01	4.06E-01	3.78E-02	4.25E-08	4.38E-01
[Sulfatide d38:1 -H]-	5.18E-13	5.17E-11	9.97E-01	7.79E-01	1.50E-03	6.27E-02	6.79E-01	3.64E-02	3.40E-01	9.30E-01	2.31E-03	7.55E-02	6.92E-01	4.19E-02	4.68E-01
[Sulfatide d40:1 -H]-	8.88E-09	1.22E-31	9.91E-01	4.71E-03	8.14E-06	3.69E-03	5.27E-03	4.75E-05	4.86E-04	1.48E-02	1.24E-05	4.39E-03	5.07E-03	5.07E-05	8.82E-04
[Sulfatide d40:2 -H]-	3.14E-07	1.59E-10	1.94E-02	3.53E-02	3.93E-06	1.10E-03	2.11E-03	9.80E-06	3.26E-02	5.25E-01	2.54E-01	3.93E-01	1.04E-01	2.35E-06	3.87E-01
[Sulfatide d41:1 -H]-	1.16E-13	2.66E-32	9.98E-01	6.52E-02	1.64E-04	2.94E-03	8.84E-03	2.03E-04	2.63E-03	3.40E-02	1.35E-04	2.63E-03	9.36E-03	1.97E-04	1.93E-03
[Sulfatide d41:2 -H]-	1.82E-09	7.86E-11	9.63E-01	6.77E-01	1.65E-02	3.98E-01	9.93E-01	4.05E-02	1.00E+00	8.72E-01	5.37E-02	6.76E-01	1.00E+00	4.81E-02	9.96E-01
[Sulfatide d42:1 -H]-	1.01E-11	7.90E-22	4.83E-01	5.96E-04	1.69E-06	3.12E-02	3.50E-03	1.15E-04	2.23E-02	2.45E-02	6.44E-06	4.83E-02	2.78E-03	1.55E-04	8.95E-02
[Sulfatide d42:2 -H]-	3.30E-06	1.49E-13	1.45E-02	6.54E-04	4.91E-08	1.38E-02	8.83E-03	2.98E-06	5.84E-01	6.71E-01	3.05E-01	5.36E-01	4.62E-04	2.96E-03	7.31E-01
[Sulfatide d42:3 -H]-	8.44E-04	2.28E-08	8.61E-01	7.44E-01	1.00E+00	1.00E+00	1.92E-02	9.63E-01	9.08E-01	1.00E+00	2.26E-01	6.68E-01	4.86E-02	4.66E-02	2.67E-02
[Sulfatide d43:3 -H]-	7.17E-09	9.56E-05	9.99E-01	9.84E-01	6.81E-02	1.82E-01	6.40E-01	8.04E-02	9.73E-01	1.00E+00	1.36E-01	2.67E-01	7.02E-01	1.36E-01	1.00E+00
[Sulfatide t41:0 -H]-	3.29E-23	3.35E-19	7.79E-01	1.00E+00	7.91E-01	1.66E-01	2.38E-01	7.09E-02	6.82E-01	7.26E-01	7.31E-01	1.60E-01	2.37E-01	7.04E-02	6.64E-01
[Sulfatide t41:1 -H]-	1.56E-16	1.96E-23	9.54E-01	3.20E-01	3.28E-03	2.97E-02	4.28E-05	2.13E-04	9.09E-03	8.08E-02	2.14E-03	2.42E-02	5.27E-03	2.11E-04	2.65E-03
[Sulfatide t42:0 -H]-	1.39E-17	6.69E-19	9.14E-01	9.21E-01	7.10E-01	4.51E-02	3.40E-01	1.13E-01	4.35E-01	1.00E+00	3.15E-01	3.85E-02	3.37E-01	1.09E-01	3.62E-01
[Sulfatide t42:1 -H]-	1.16E-14	4.92E-27	1.00E+00	2.30E-01	6.64E-04	1.40E-02	5.65E-02	6.75E-04	2.50E-02	2.06E-01	6.27E-04	1.36E-02	5.62E-02	6.68E-04	2.33E-02

Supplementary Table S1. Continued

Lipid specie	Levene test	ANOVA	PTII vs CDT	PTII vs CMT	PTII vs MTI	PTII vs MTII	PTII vs ITS	CDT vs CMT	CDT vs MTI	CDT vs MTII	CDT vs ITS	CMT vs MTI	CMT vs MTII	CMT vs ItS	MTI vs MTII	MTI vs ITS	MTII vs ITS
[HexCer d28:0 -H]-	1.50E-04	1.45E-17	0.00E+00	0.00E+00	4.00E-03	0.00E+00	9.88E-01	1.00E+00	6.38E-01	1.10E-02	1.00E-03	7.09E-01	3.40E-02	1.00E-03	9.81E-01	1.00E-03	0.00E+00
[HexCer d29:0 -H]-	3.73E-09	5.55E-18	1.30E-02	4.00E-03	1.00E-03	1.00E-03	8.15E-01	8.40E-01	2.45E-01	3.00E-03	1.83E-01	8.94E-01	6.35E-01	6.10E-02	1.00E+00	1.70E-02	9.00E-03
[HexCer d30:0 -H]-	3.35E-06	1.50E-16	0.00E+00	0.00E+00	0.00E+00	0.00E+00	1.00E+00	1.00E+00	1.43E-01	0.00E+00	2.00E-03	1.70E-01	4.00E-03	2.00E-03	9.99E-01	0.00E+00	0.00E+00
[HexCer d30:1 -H]-	1.91E-06	1.10E-33	0.00E+00	0.00E+00	3.20E-02	0.00E+00	6.45E-01	8.35E-01	6.98E-01	0.00E+00	0.00E+00	9.12E-01	2.50E-02	0.00E+00	9.99E-01	9.00E-03	0.00E+00
[HexCer d32:0 -H]-	1.56E-04	3.18E-15	4.90E-01	1.00E-03	0.00E+00	0.00E+00	2.10E-02	1.70E-02	0.00E+00	0.00E+00	4.69E-01	1.32E-01	4.49E-01	8.95E-01	9.64E-01	4.00E-02	1.12E-01
[HexCer d34:1 -H]-	1.82E-07	6.92E-19	3.00E-03	0.00E+00	0.00E+00	0.00E+00	0.00E+00	1.60E-02	1.00E-03	1.00E-03	7.60E-01	3.93E-01	3.98E-01	3.50E-01	1.00E+00	1.70E-02	1.50E-02
[HexCer d36:1 -H]-	9.49E-03	1.14E-19	0.00E+00	0.00E+00	0.00E+00	0.00E+00	9.95E-01	7.90E-02	7.00E-03	3.40E-02	1.47E-01	7.70E-02	8.57E-01	9.00E-03	7.57E-01	0.00E+00	2.00E-03
[HexCer d36:2 -H]-	3.44E-06	1.95E-21	0.00E+00	0.00E+00	0.00E+00	0.00E+00	1.70E-02	8.60E-02	8.50E-02	1.00E-03	1.04E-01	9.10E-01	3.26E-01	5.00E-03	1.00E+00	2.00E-03	1.00E-03
[HexCer d38:2 -H]-	4.89E-06	1.23E-11	1.63E-01	6.00E-03	6.00E-03	0.00E+00	9.83E-01	1.74E-01	2.13E-01	0.00E+00	3.80E-02	9.15E-01	1.57E-01	2.00E-03	9.98E-01	2.00E-03	0.00E+00
[HexCer t35:1 -H]-	9.14E-03	1.65E-18	4.30E-02	0.00E+00	2.96E-01	2.37E-01	0.00E+00	1.50E-02	7.28E-01	9.98E-01	4.50E-02	9.83E-01	6.00E-03	1.00E+00	6.05E-01	9.99E-01	1.70E-02
[HexCer t35:2 -H]-	1.22E-01	9.12E-17	5.00E-03	0.00E+00	3.94E-01	8.91E-01	0.00E+00	2.03E-01	1.00E+00	7.01E-01	8.35E-01	9.24E-01	9.00E-03	9.46E-01	9.31E-01	9.99E-01	1.01E-01
[Lyso-PI 16:0-H]-	2.43E-06	4.39E-07	1.00E+00	8.06E-01	9.99E-01	4.90E-01	7.00E-03	5.26E-01	9.93E-01	1.48E-01	0.00E+00	9.91E-01	1.00E+00	2.82E-01	9.54E-01	5.09E-01	9.50E-02
[Lyso-PI 18:0-H]-	1.27E-01	4.66E-11	6.70E-01	6.60E-02	9.54E-01	1.00E-03	2.00E-03	8.52E-01	1.00E+00	9.20E-02	3.03E-01	9.99E-01	8.10E-01	9.97E-01	7.91E-01	9.72E-01	9.82E-01
[Lyso-PI 18:1-H]-	2.64E-08	3.07E-27	9.87E-01	9.90E-01	4.30E-02	1.00E+00	7.07E-01	9.14E-01	4.40E-02	9.33E-01	9.80E-01	2.00E-02	9.97E-01	7.57E-01	4.30E-02	4.20E-02	5.57E-01
[Lyso-PI 20:4-H]-	9.69E-06	7.71E-23	8.63E-01	1.00E+00	4.70E-02	1.85E-01	1.15E-01	9.93E-01	5.10E-02	6.13E-01	3.68E-01	2.90E-02	7.81E-01	7.07E-01	4.90E-02	4.90E-02	1.00E+00
[PC 32:0-CH3]-	2.63E-01	2.03E-27	2.19E-01	9.96E-01	9.85E-01	6.54E-01	0.00E+00	8.23E-01	1.00E+00	1.00E+00	0.00E+00	1.00E+00	9.70E-01	0.00E+00	1.00E+00	0.00E+00	0.00E+00
[PC 32:2-CH3]-	5.49E-11	2.47E-33	2.00E-03	0.00E+00	2.30E-02	0.00E+00	0.00E+00	6.00E-03	3.54E-01	3.00E-03	0.00E+00	9.25E-01	9.80E-01	1.43E-01	9.95E-01	1.00E+00	6.86E-01
[PC 33:2-CH3]-	6.19E-01	8.92E-06	4.66E-01	3.00E-03	9.50E-02	8.66E-01	1.00E+00	3.90E-01	6.31E-01	1.00E+00	7.78E-01	1.00E+00	4.85E-01	1.40E-02	6.17E-01	1.67E-01	9.69E-01
[PC 34:1-CH3]-	9.45E-05	8.43E-18	5.60E-02	1.00E-03	1.30E-01	9.08E-01	3.00E-03	5.24E-01	6.62E-01	9.32E-01	1.00E+00	1.00E+00	2.04E-01	4.17E-01	3.56E-01	6.60E-01	7.85E-01
[PC 34:4-CH3]-	1.21E-03	1.31E-31	2.36E-01	1.00E-03	0.00E+00	3.00E-03	0.00E+00	2.10E-02	1.10E-02	1.16E-01	0.00E+00	6.20E-01	1.00E+00	1.94E-01	5.92E-01	9.82E-01	2.27E-01
[PC 35:1-CH3]-	3.97E-03	1.15E-15	1.10E-02	0.00E+00	9.48E-01	3.54E-01	3.40E-02	1.90E-02	9.55E-01	8.55E-01	9.73E-01	3.13E-01	1.00E-03	0.00E+00	1.00E+00	9.99E-01	9.99E-01
[PC 35:2-CH3]-	1.67E-02	1.94E-03	1.00E+00	7.27E-01	8.84E-01	1.00E+00	6.48E-01	7.08E-01	8.83E-01	1.00E+00	6.14E-01	9.97E-01	8.23E-01	3.10E-02	8.89E-01	6.70E-01	8.89E-01
[PC 36:1-CH3]-	2.07E-03	5.43E-25	2.00E-03	0.00E+00	6.26E-01	1.00E+00	0.00E+00	9.48E-01	8.95E-01	1.62E-01	2.33E-01	9.49E-01	5.00E-02	7.54E-01	6.57E-01	9.96E-01	6.00E-03
[PC 36:2-CH3]-	3.91E-01	2.16E-10	9.80E-01	4.50E-02	8.60E-02	1.00E+00	3.18E-01	3.44E-01	2.62E-01	1.00E+00	8.90E-01	9.64E-01	3.55E-01	9.77E-01	2.27E-01	7.15E-01	8.33E-01
[PC 36:5-CH3]-	3.93E-04	5.71E-13	2.00E-03	9.05E-01	1.00E+00	2.13E-01	1.94E-01	7.52E-01	1.00E-03	9.70E-01	0.00E+00	8.29E-01	9.94E-01	1.35E-01	1.31E-01	1.02E-01	5.00E-03
[PC 36:6-CH3]-	5.83E-06	1.11E-32	0.00E+00	0.00E+00	0.00E+00	0.00E+00	0.00E+00	3.59E-01	7.80E-02	0.00E+00	5.40E-02	4.03E-01	1.70E-02	9.65E-01	1.00E+00	8.29E-01	2.79E-01
[PC 38:4-CH3]-	6.67E-02	8.09E-23	5.80E-01	9.88E-01	1.00E+00	1.40E-02	0.00E+00	9.94E-01	9.31E-01	4.79E-01	0.00E+00	9.96E-01	1.96E-01	0.00E+00	2.83E-01	6.00E-03	0.00E+00
[PC 38:5-CH3]-	5.02E-01	2.89E-08	1.00E+00	9.99E-01	0.00E+00	1.14E-01	6.80E-02	1.00E+00	1.00E-03	4.90E-02	2.26E-01	2.00E-03	6.10E-02	4.34E-01	0.00E+00	7.50E-02	0.00E+00
[PC 38:6-CH3]-	4.97E-04	1.26E-15	2.00E-03	5.00E-03	0.00E+00	0.00E+00	8.21E-01	1.00E+00	9.99E-01	6.00E-03	0.00E+00	9.29E-01	2.00E-03	0.00E+00	4.00E-03	0.00E+00	0.00E+00
[PC 38:7-CH3]-	1.72E-10	1.54E-10	3.61E-01	2.00E-02	1.00E-02	7.00E-03	5.70E-02	3.66E-01	1.69E-01	5.70E-02	8.22E-01	9.85E-01	9.71E-01	9.85E-01	1.00E+00	7.12E-01	4.91E-01
[PC O-32:2/ PC P-32:1-CH3]-	2.82E-01	8.17E-02															
[PC O-34:2/ PC P-34:1-CH3]-	3.13E-02	2.74E-13	2.44E-01	0.00E+00	3.29E-01	6.27E-01	0.00E+00	1.70E-02	7.40E-01	9.97E-01	5.00E-02	9.83E-01	6.00E-03	1.00E+00	6.05E-01	9.99E-01	1.70E-02
[PC O-34:3/ PC P-34:2-CH3]-	3.81E-01	4.18E-04	5.00E-01	2.74E-01	9.95E-01	1.00E+00	5.28E-01	9.99E-01	1.00E+00	8.80E-01	1.00E+00	9.99E-01	6.71E-01	9.99E-01	9.99E-01	1.00E+00	8.85E-01
[PC O-34:5/ PC P-34:4-CH3]-	2.37E-01	1.15E-04	9.78E-01	1.00E+00	9.99E-01	2.38E-01	1.00E+00	9.94E-01	9.56E-01	4.30E-02	9.94E-01	9.98E-01	3.06E-01	1.00E+00	9.87E-01	9.98E-01	2.26E-01
[PC O-36:5/ PC P-36:4-CH3]-	6.38E-05	3.28E-16	6.79E-01	1.70E-02	7.60E-02	9.64E-01	0.00E+00	8.90E-02	1.59E-01	9.41E-01	0.00E+00	1.00E+00	3.30E-02	1.00E+00	1.40E-01	1.00E+00	0.00E+00
[PC O-36:6/ PC P-36:5-CH3]-	4.30E-01	3.34E-15	9.98E-01	8.35E-01	1.00E+00	8.37E-01	1.20E-02	9.89E-01	1.00E+00	5.44E-01	8.50E-02	9.97E-01	2.07E-01	6.35E-01	9.78E-01	6.55E-01	1.00E-03
[PC O-36:7/ PC P-34:1-CH3]-	4.67E-02	4.82E-20	9.71E-01	7.70E-01	3.20E-02	1.60E-02	6.00E-03	9.99E-01	2.00E-02	5.00E-02	0.00E+00	3.00E-02	2.70E-02	0.00E+00	3.80E-02	9.22E-01	0.00E+00
[PC O-38:5/ PC P-38:4-CH3]-	5.58E-10	2.79E-25	1.00E+00	4.40E-01	6.27E-01	1.61E-01	0.00E+00	3.09E-01	6.00E-01	8.10E-02	0.00E+00	7.20E-02	2.80E-02	1.00E-03	9.71E-01	0.00E+00	0.00E+00
[PC O-38:6/ PC P-38:5-CH3]-	4.72E-13	3.75E-24	9.99E-01	1.00E+00	9.94E-01	5.98E-01	0.00E+00	9.30E-01	9.76E-01	8.35E-01	0.00E+00	9.99E-01	2.47E-01	0.00E+00	8.86E-01	1.52E-01	0.00E+00
[PC O-38:7/ PC P-38:6-CH3]-	4.64E-17	4.61E-33	7.33E-01	9.99E-01	1.67E-01	1.00E-03	0.00E+00	4.84E-01	7.22E-01	1.90E-02	0.00E+00	1.06E-01	6.00E-03	0.00E+00	8.65E-01	0.00E+00	0.00E+00
[PE 34:0-H]-	2.63E-01	2.03E-27	2.19E-01	9.96E-01	9.85E-01	6.54E-01	0.00E+00	8.23E-01	1.00E+00	1.00E+00	0.00E+00	1.00E+00	9.70E-01	0.00E+00	1.00E+00	0.00E+00	0.00E+00
[PE 34:1-H]-	4.58E-04	1.81E-08	2.46E-01	1.00E+00	3.11E-01	7.81E-01	2.52E-01	3.27E-01	1.00E+00	0.00E+00	0.00E+00	3.95E-01	5.51E-01	1.35E-01	3.60E-02	4.00E-03	6.08E-01
[PE 34:2-H]-	5.49E-11	2.47E-33	2.00E-03	0.00E+00	2.30E-02	0.00E+00	0.00E+00	6.00E-03	3.54E-01	3.00E-03	0.00E+00	9.25E-01	9.80E-01	1.43E-01	9.95E-01	1.00E+00	6.86E-01
[PE 35:2-H]-	6.19E-01	8.92E-06	4.66E-01	3.00E-03	9.50E-02	8.66E-01	1.00E+00	3.90E-01	6.31E-01	1.00E+00	7.78E-01	1.00E+00	4.85E-01	1.40E-02	6.17E-01	1.67E-01	9.69E-01
[PE 36:1-H]-	1.06E-04	4.57E-17	5.60E-02	1.00E-03	4.92E-01	9.08E-01	3.00E-03	5.24E-01	1.00E+00	9.32E-01	1.00E+00	9.16E-01	2.04E-01	4.17E-01	9.44E-01	1.00E+00	7.85E-01
[PE 36:2-H]-	8.35E-05	1.72E-05	4.57E-01	3.08E-01	1.00E+00	4.75E-01	0.00E+00	1.00E+00	3.65E-01	9.98E-01	1.64E-01	1.68E-01	9.93E-01	5.70E-02	1.58E-01	0.00E+00	0.00E+00
[PE 36:3-H]-	3.99E-02	3.54E-20	9.06E-01	9.92E-01	1.20E-02	4.10E-01	0.00E+00	1.00E+00	1.60E-02	2.70E-02	0.00E+00	4.00E-03	2.14E-01	1.50E-02	3.40E-02	8.00E-03	0.00E+00
[PE 36:4-H]-	1.21E-03	1.31E-31	2.36E-01	1.00E-03	0.00E+00	3.00E-03	0.00E+00	2.10E-02	1.10E-02	1.16E-01	0.00E+00	6.20E-01	1.00E+00	1.94E-01	5.92E-01	9.82E-01	2.27E-01
[PE 37:1-H]-	3.97E-03	1.15E-15	1.10E-02	0.00E+00	9.48E-01	3.54E-01	3.40E-02	1.90E-02	9.55E-01	8.55E-01	9.73E-01	3.13E-01	1.00E-03	0.00E+00	1.00E+00	9.99E-01	9.99E-01
[PE 37:2-H]-	1.67E-02	1.94E-03	1.00E+00	7.27E-01	8.84E-01	1.00E+00	6.48E-01	7.08E-01	8.83E-01	1.00E+00	6.14E-01	9.97E-01	8.23E-01	3.10E-02	8.89E-01	6.70E-01	8.89E-01
[PE 38:1-H]-	2.14E-07	1.38E-17	1.00E-03	0.00E+00	6.26E-01	9.30E-01	1.00E-03	9.83E-01	9.06E-01	1.00E+00	4.00E-02	9.49E-01	1.00E+00	9.10E-02	9.38E-01	1.00E+00	4.06E-01
[PE 38:2-H]-	2.58E-01	2.14E-10	9.77E-01	3.50E-02	6.90E-02	1.00E+00	2.82E-01	3.07E-01	2.28E-01	1.00E+00	8.74E-01	9.58E-01	3.17E-01	9.72E-01	1.96E-01	6.83E-01	8.11E-01
[PE 38:4-H]-	6.24E-02	2.60E-07	7.83E-01	2.88E-01	6.95E-01	4.00E-02	5.64E-01	9.83E-01	9.86E-01	5.46E-01	1.00E+00	1.00E+00	9.71E-01	9.99E-01	1.00E+00	9.97E-01	7.68E-01
[PE 38:5-H]-	3.93E-04	5.71E-13	2.00E-03	9.05E-01	1.00E+00	2.13E-01	1.94E-01	7.52E-01	1.00E-03	9.70E-01	0.00E+00	8.29E-01	9.94E-01	1.35E-01	1.31E-01	1.02E-01	5.00E-03
[PE 38:6-H]-	5.83E-06	1.11E-32	0.00E+00	0.00E+00	0.00E+00	0.00E+00	0.00E+00	3.59E-01	7.80E-02	0.00E+00	5.40E-02	4.03E-01	1.70E-02	9.65E-01	1.00E+00	8.29E-01	2.79E-01
[PE 40:4-H]-	6.67E-02	8.09E-23	5.80E-01	9.88E-01	1.00E+00	1.40E-02	0.00E+00	9.94E-01	9.31E-01	4.79E-01	0.00E+00	9.96E-01	1.96E-01	0.00E+00	2.83E-01	6.00E-03	0.00E+00
[PE 40:5-H]-	7.85E-01	4.49E-07	9.98E-01	9.64E-01	9.96E-01	8.00E-01	1.00E-03	1.00E+00	1.00E+00	5.00E-01	9.00E-03	1.00E+00	3.30E-01	1.00E-01	7.58E-01	7.89E-01	0.00E+00
[PE 40:6-H]-	4.97E-04	1.26E-15	2.00E-03	5.00E-03	0.00E+00	0.00E+00	8.21E-01	1.00E+00	9.99E-01	6.00E-03	0.00E+00	9.29E-01	2.00E-03	0.00E+00	4.00E-03	0.00E+00	0.00E+00
[PE 40:7-H]-	1.70E-10	1.56E-10	3.61E-01	2.00E-02	1.00E-02	7.00E-03	5.80E-02	3.66E-01	1.69E-01	5.70E-02	8.30E-01	9.85E-01	9.71E-01	9.83E-01	1.00E+00	7.02E-01	4.75E-01
[PE O-34:2/ PE P-34:1-H]-	2.82E-01	8.17E-02															
[PE O-34:3/ PE P-34:2-H]-	3.26E-02	7.73E-01															
[PE O-36:2/ PE P-36:1-H]-	2.09E-01	5.17E-02															
[PE O-36:3/ PE P-36:2-H]-	1.87E-01	7.54E-12	9.10E-02	0.00E+00	7.14E-01	9.84E-01	2.00E-03	4.05E-01	1.00E+00	8.82E-01	9.14E-01	9.72E-01	6.10E-02	9.81E-01	9.75E-01	1.00E+00	2.88E-01
[PE O-36:5/ PE P-36:4-H]-	2.35E-01	1.21E-04	9.79E-01	1.00E+00	9.99E-01	2.45E-01	1.00E+00	9.95E-01	9.59E-01	4.50E-02	9.94E-01	9.98E-01	3.10E-01	1.00E+00	9.87E-01	9.98E-01	2.29E-01
[PE O-38:5/ PE P-38:4-H]-	2.56E-07	6.00E-12	7.50E-02	1.10E-02	0.00E+00	7.13E-01	0.00E+00	2.03E-01	3.89E-01	3.50E-01	1.30E-02	5.79E-01	3.40E-02	1.00E+00	1.00E-03	1.60E-01	0.00E+00
[PE O-38:6/ PE P-38:5-H]-	4.95E-02	7.47E-15	9.36E-01	3.84E-01	2.80E-02	2.35E-01	1.90E-02	9.94E-01	1.89E-01	6.90E-02	4.95E-01	4.27E-01	4.00E-03	8.57E-01	2.60E-02	9.95E-01	0.00E+00
[PE O-38:7/ PE P-38:6-H]-	4.60E-03	1.51E-29	9.29E-01	7.80E-01	4.40E-02	0.00E+00	0.00E+00	1.00E+00	3.20E-02	8.00E-03	0.00E+00	3.00E-02	2.70E-02	0.00E+00	3.80E-02	9.22E-01	0.00E+00
[PE O-40:5/ PE P-40:4-H]-	3.99E-08	7.02E-32	9.99E-01	1.87E-01	5.00E-02	7.85E-01	0.00E+00	1.12E-01	5.10E-02	9.37E-01	0.00E+00	4.40E-02	5.40E-02	1.80E-02	5.00E-02	1.17E-01	0.00E+00
[PE O-40:6/ PE P-40:5-H]-	2.26E-07	4.56E-33	1.00E+00	4.97E-01	6.00E-02	3.55E-01	0.00E+00	6.08E-01	6.10E-02	8.90E-02	0.00E+00	5.40E-02	1.23E-01	8.00E-02	5.80E-02	1.04E-01	0.00E+00
[PE O-40:7/ PE P-40:6-H]-	5.51E-10	4.78E-36	1.00E+00	6.56E-01	6.10E-02	3.10E-02	0.00E+00	5.14E-01	6.30E-02	1.00E-02	0.00E+00	6.10E-02	2.00E-02	0.00E+00	5.80E-02	1.86E-01	0.00E+00
[PG40:7-H]-	2.16E-16	1.32E-40	9.99E-01	4.65E-01	5.20E-02	1.00E+00	6.10E-01	4.06E-01	5.10E-02	9.93E-01	9.71E-01	3.40E-02	4.92E-01	3.23E-01	5.20E-02	5.10E-02	5.70E-01
[PI 32:0-H]-	4.91E-09	3.24E-14	6.49E-01	8.92E-01	1.60E-01	9.87E-01	8.00E-03	1.00E+00	7.29E-01	8.21E-01	3.00E-03	9.62E-01	9.91E-01	3.46E-01	1.39E-01	3.41E-01	1.00E-03
[PI 34:1-H]-	2.68E-04	3.14E-16	0.00E+00	7.40E-02	1.56E-01	7.00E-03	1.50E-02	1.00E+00	1.00E+00	9.99E-01	0.00E+00	1.00E+00	9.99E-01	2.00E-03	9.99E-01	3.10E-02	0.00E+00
[PI 34:2-H]-	1.52E-02	1.14E-19	0.00E+00	0.00E+00	1.97E-01	1.00E-03	8.64E-01	4.13E-01	9.90E-01	7.70E-02	0.00E+00	1.00E+00	9.51E-01	1.00E-03	9.55E-01	3.04E-01	2.50E-02
[PI 36:1-H]-	1.63E-03	1.52E-24	2.01E-01	6.52E-01	9.16E-01	6.04E-01	0.00E+00	1.00E+00	9.98E-01	1.00E+00	0.00E+00	1.00E+00	1.00E+00	3.00E-02	1.00E+00	2.28E-01	2.00E-03
[PI 36:2-H]-	1.20E-02	5.45E-21	0.00E+00	0.00E+00	9.00E-03	0.00E+00	1.00E+00	7.69E-01	1.00E+00	1.00E+00	0.00E+00	7.64E-01	3.17E-01	1.00E-03	1.00E+00	1.80E-02	0.00E+00
[PI 36:3-H]-	3.27E-01	3.19E-17	5.44E-01	1.23E-01	0.00E+00	9.99E-01	2.93E-01	9.74E-01	0.00E+00	9.84E-01	2.00E-03	0.00E+00	6.98E-01	0.00E+00	0.00E+00	0.00E+00	2.50E-01
[PI 36:4-H]-	6.80E-02	8.36E-06	8.00E-03	1.00E+00	7.21E-01	9.74E-01	9.91E-01	8.00E-03	1.00E+00	4.00E-03	1.00E-03	5.98E-01	9.99E-01	1.00E+00	4.08E-01	4.47E-01	1.00E+00
[PI 38:2-H]-	9.31E-07	1.02E-17	9.50E-02	6.06E-01	6.60E-02	3.00E-03	2.00E-03	9.95E-01	6.80E-02	0.00E+00	0.00E+00	4.70E-02	2.49E-01	1.85E-01	5.80E-02	5.70E-02	6.34E-01
[PI 38:4-H]-	1.73E-02	3.44E-05	1.10E-02	8.64E-01	1.42E-01	5.60E-02	6.74E-01	9.73E-01	0.00E+00	0.00E+00	3.40E-01	1.42E-01	3.30E-02	1.00E+00	5.24E-01	0.00E+00	3.00E-03
[PI 38:5-H]-	3.19E-03	8.92E-10	0.00E+00	1.82E-01	1.60E-02	9.86E-01	0.00E+00	5.01E-01	5.10E-01	0.00E+00	6.69E-01	9.20E-02	4.20E-02	7.40E-02	2.40E-02	1.00E+00	0.00E+00
[PI 38:6-H]-	1.86E-06	1.95E-22	5.56E-01	1.80E-02	3.10E-02	0.00E+00	0.00E+00	2.53E-01	2.13E-01	0.00E+00	0.00E+00	8.75E-01	3.30E-02	4.50E-02	9.20E-01	9.49E-01	1.00E+00
[PI 40:4-H]-	1.57E-03	8.14E-27	9.26E-01	3.80E-01	5.00E-02	2.00E-01	1.00E+00	7.16E-01	5.60E-02	1.00E-03	7.67E-01	4.40E-02	4.30E-02	3.21E-01	5.00E-02	5.40E-02	1.00E-02
[PI 40:5-H]-	2.93E-01	1.80E-15	9.51E-01	9.50E-01	0.00E+00	9.90E-01	1.00E+00	1.00E+00	0.00E+00	6.78E-01	9.23E-01	0.00E+00	6.90E-01	9.24E-01	0.00E+00	0.00E+00	9.97E-01
[PI 40:6-H]-	2.07E-04	5.27E-15	2.09E-01	7.80E-02	5.10E-02	8.60E-02	0.00E+00	9.48E-01	4.46E-01	8.56E-01	1.00E-03	9.39E-01	1.00E+00	5.66E-01	9.97E-01	1.00E+00	9.41E-01
[PS 34:1-H]-	5.56E-05	7.60E-36	0.00E+00	1.00E-03	4.20E-02	0.00E+00	3.44E-01	9.98E-01	6.30E-02	3.17E-01	0.00E+00	5.00E-02	4.88E-01	1.00E-02	5.90E-02	4.20E-02	6.00E-03
[PS 36:1-H]-	2.68E-08	1.38E-26	0.00E+00	3.00E-03	5.68E-01	7.57E-01	0.00E+00	1.00E+00	2.71E-01	8.80E-02	1.00E-03	2.31E-01	1.03E-01	7.00E-03	1.00E+00	0.00E+00	0.00E+00
[PS 36:2-H]-	1.72E-08	1.33E-16	1.00E+00	1.00E+00	4.30E-02	6.05E-01	7.18E-01	1.00E+00	6.30E-02	4.80E-02	2.46E-01	3.80E-02	8.46E-01	9.08E-01	5.70E-02	5.40E-02	1.00E+00
[PS 36:4-H]-	2.09E-07	9.41E-12	1.00E+00	9.70E-01	9.92E-01	9.98E-01	8.60E-02	6.78E-01	7.78E-01	8.17E-01	1.00E-03	1.00E+00	9.97E-01	4.52E-01	1.00E+00	3.21E-01	8.00E-03
[PS 38:3-H]-	1.44E-13	6.68E-31	1.00E+00	8.42E-01	5.40E-02	9.26E-01	3.00E-03	7.89E-01	5.40E-02	9.95E-01	2.00E-03	3.40E-02	7.12E-01	9.99E-01	5.30E-02	6.20E-02	1.00E-03
[PS 38:4-H]-	4.74E-04	7.22E-14	6.47E-01	8.43E-01	9.99E-01	1.50E-02	1.20E-02	1.00E+00	3.54E-01	1.50E-01	0.00E+00	4.96E-01	1.30E-01	0.00E+00	1.01E-01	1.07E-01	0.00E+00
[PS 38:5-H]-	1.63E-07	4.14E-05	6.03E-01	1.77E-01	9.60E-02	5.20E-02	3.60E-02	7.30E-01	2.88E-01	5.90E-02	1.90E-02	9.98E-01	8.64E-01	5.90E-01	9.72E-01	7.43E-01	9.97E-01
[PS 40:7-H]-	1.56E-06	2.22E-20	5.00E-03	1.00E-03	7.00E-03	0.00E+00	7.00E-03	6.93E-01	9.22E-01	4.00E-02	1.00E+00	1.00E+00	6.20E-01	3.42E-01	9.59E-01	8.13E-01	6.00E-03
[[PS O-38:2/ PS P-38:1-H]-	4.92E-09	6.29E-13	2.20E-01	3.60E-02	1.50E-02	4.00E-03	9.94E-01	6.37E-01	2.84E-01	3.00E-03	7.09E-01	9.70E-01	8.92E-01	2.00E-01	1.00E+00	7.80E-02	3.20E-02
[SM d32:1-CH3]-	4.13E-05	4.87E-24	1.30E-03	1.92E-05	1.42E-06	2.52E-06	8.83E-01	8.05E-02	1.83E-03	6.48E-03	3.44E-02	1.66E-01	6.68E-01	4.60E-04	9.87E-01	2.91E-05	5.79E-05
[SM d33:1-CH3]-	4.88E-06	2.68E-27	7.41E-05	5.96E-06	2.31E-06	2.32E-06	1.17E-02	4.47E-02	2.08E-02	2.80E-03	1.83E-01	7.56E-01	8.94E-01	5.68E-03	1.00E+00	1.29E-03	1.27E-03
[SM d34:1-CH3]-	4.20E-05	8.68E-26	2.56E-04	2.74E-05	4.87E-05	1.34E-05	9.95E-01	5.83E-01	2.70E-01	2.39E-01	7.71E-08	8.28E-01	8.54E-01	8.07E-09	1.00E+00	2.67E-05	2.45E-07
[SM d34:2-CH3]-	3.32E-06	6.55E-25	7.87E-05	4.46E-05	4.61E-06	2.76E-06	7.17E-01	9.96E-01	1.38E-01	3.75E-04	5.28E-05	2.25E-01	1.37E-02	2.43E-05	1.00E+00	1.19E-05	6.54E-07
[SM d35:1-CH3]-	3.09E-07	1.35E-26	1.08E-04	3.22E-05	2.07E-06	9.85E-07	9.94E-01	9.55E-01	4.75E-02	3.50E-04	5.35E-04	1.75E-01	5.73E-02	2.18E-04	1.00E+00	2.31E-05	2.29E-05
[SM d36:1-CH3]-	5.66E-10	1.19E-23	9.55E-08	1.65E-08	1.16E-05	4.62E-11	8.93E-01	7.22E-01	1.17E-01	2.09E-05	1.56E-08	3.21E-01	4.03E-02	2.24E-08	1.00E+00	4.36E-04	8.95E-12
[SM d36:2-CH3]-	1.19E-07	5.22E-19	2.51E-04	9.04E-05	2.97E-05	3.08E-05	5.48E-02	5.05E-01	4.25E-03	2.12E-04	1.26E-02	2.25E-01	1.04E-01	1.96E-03	1.00E+00	2.58E-04	2.66E-04
[SM d38:1-CH3]-	1.57E-03	3.35E-11	9.86E-01	1.70E-02	4.39E-04	2.45E-04	5.75E-01	3.63E-03	4.59E-05	1.07E-06	8.11E-01	2.14E-01	9.77E-02	7.98E-02	1.00E+00	6.87E-04	1.49E-05
[SM d38:2-CH3]-	1.55E-11	6.86E-17	9.47E-03	4.08E-04	2.17E-04	2.30E-04	8.30E-04	5.79E-03	6.70E-04	8.15E-04	1.20E-01	7.80E-02	1.39E-01	8.58E-01	7.86E-01	1.40E-01	1.79E-01
[SM d40:1-CH3]-	4.00E-01	1.89E-09	3.42E-01	5.89E-05	9.40E-03	1.90E-06	9.24E-01	7.68E-02	2.14E-01	3.53E-03	9.84E-01	9.95E-01	9.19E-01	7.94E-03	1.00E+00	7.17E-02	2.81E-04
[SM d40:2-CH3]-	2.79E-02	6.81E-26	8.99E-08	2.57E-05	1.83E-01	3.89E-10	6.38E-03	1.00E+00	6.71E-02	9.32E-07	3.04E-03	3.76E-02	3.97E-01	6.96E-02	5.11E-02	8.68E-02	1.73E-06
[SM d42:1-CH3]-	2.82E-09	4.98E-01															
[SM d42:2-CH3]-	1.93E-02	1.28E-32	1.37E-06	5.64E-09	2.84E-05	6.92E-10	1.00E+00	7.18E-03	1.75E-01	4.40E-06	5.19E-07	9.98E-01	3.28E-01	2.28E-09	9.27E-01	1.35E-04	4.52E-10
[SM d42:3-CH3]-	6.21E-04	5.36E-45	1.55E-10	5.22E-12	1.03E-05	2.72E-12	3.09E-01	1.20E-02	2.62E-01	2.38E-08	7.49E-08	9.31E-01	1.44E-02	1.92E-09	9.48E-01	1.36E-04	5.96E-10
[Sulfatide d34:1 -H]-	3.79E-04	5.95E-14	3.97E-01	7.98E-01	9.97E-01	2.48E-03	1.00E+00	1.00E+00	9.59E-01	1.30E-01	1.48E-01	9.94E-01	2.32E-01	6.12E-01	2.04E-01	9.79E-01	1.74E-03
[Sulfatide d34:2 -H]-	1.69E-10	1.01E-37	5.09E-01	9.98E-01	6.06E-02	8.65E-02	2.05E-02	7.90E-01	5.87E-02	7.99E-01	1.60E-01	4.34E-02	5.82E-01	4.44E-01	5.78E-02	5.68E-02	5.17E-01
[Sulfatide d34:3 -H]-	2.78E-02	3.66E-07	9.98E-01	9.04E-01	5.55E-04	7.75E-01	8.33E-01	2.80E-01	9.80E-03	1.81E-03	5.88E-02	3.73E-03	1.00E+00	1.00E+00	3.71E-02	1.01E-02	1.00E+00
[Sulfatide d36:3 -H]-	4.82E-09	1.67E-29	1.00E+00	3.17E-01	3.44E-02	3.76E-02	3.01E-01	2.33E-01	3.73E-02	9.84E-04	1.04E-01	1.83E-02	9.38E-01	9.52E-01	4.59E-02	3.67E-02	1.00E+00
[Sulfatide d36:4 -H]-	3.03E-10	5.59E-27	9.98E-01	3.80E-01	3.74E-02	2.57E-08	4.03E-01	3.29E-01	3.66E-02	8.73E-09	3.33E-01	1.35E-02	1.00E+00	1.00E+00	5.10E-02	1.86E-02	9.86E-01
[Sulfatide d38:1 -H]-	5.18E-13	5.17E-11	1.08E-02	1.34E-01	7.27E-01	6.17E-02	9.39E-01	9.99E-01	9.59E-01	8.49E-01	1.50E-01	9.89E-01	9.95E-01	3.36E-01	1.00E+00	7.88E-01	1.29E-01
[Sulfatide d40:1 -H]-	8.88E-09	1.22E-31	8.37E-04	2.08E-02	3.71E-04	6.75E-05	2.33E-01	8.53E-01	4.51E-01	3.88E-04	1.98E-01	1.00E+00	1.05E-02	1.40E-01	2.70E-03	9.82E-04	9.81E-05
[Sulfatide d40:2 -H]-	3.14E-07	1.59E-10	9.90E-01	9.95E-01	9.83E-01	1.10E-01	1.11E-01	1.00E+00	1.00E+00	9.57E-05	1.04E-04	1.00E+00	2.31E-04	7.59E-03	3.32E-04	7.51E-05	9.07E-06
[Sulfatide d41:1 -H]-	1.16E-13	2.66E-32	1.93E-03	1.17E-02	6.22E-04	2.66E-04	1.41E-01	9.98E-01	9.96E-01	1.56E-03	2.11E-01	1.00E+00	3.20E-03	1.78E-01	3.67E-03	3.60E-03	4.41E-04
[Sulfatide d41:2 -H]-	1.82E-09	7.86E-11	9.79E-01	1.00E+00	9.95E-01	1.12E-01	7.34E-01	9.60E-01	7.36E-01	1.95E-01	2.26E-02	9.88E-01	1.08E-01	4.67E-01	7.53E-02	9.97E-01	4.21E-02
[Sulfatide d42:1 -H]-	1.01E-11	7.90E-22	8.34E-04	1.60E-01	6.71E-04	3.58E-04	9.42E-01	9.73E-01	2.43E-05	4.51E-02	1.27E-01	7.64E-03	9.62E-01	3.58E-01	2.28E-03	1.90E-06	6.58E-04
[Sulfatide d42:2 -H]-	3.30E-06	1.49E-13	1.00E+00	9.99E-01	3.61E-04	6.05E-01	5.24E-02	9.97E-01	5.91E-03	1.96E-01	2.60E-03	9.44E-04	9.96E-01	9.69E-02	1.03E-02	4.96E-04	2.31E-05
[Sulfatide d42:3 -H]-	8.44E-04	2.28E-08	3.26E-02	3.80E-01	6.67E-02	2.79E-03	1.30E-03	9.94E-01	6.36E-02	2.42E-02	3.14E-04	4.50E-02	5.46E-01	3.44E-01	5.75E-02	5.67E-02	6.81E-01
[Sulfatide d43:3 -H]-	7.17E-09	9.56E-05	2.53E-01	3.71E-01	7.57E-01	2.21E-01	1.00E+00	1.00E+00	1.00E+00	1.00E+00	1.92E-01	1.00E+00	1.00E+00	3.30E-01	1.00E+00	7.42E-01	1.81E-01
[Sulfatide t41:0 -H]-	3.29E-23	3.35E-19	7.95E-01	1.67E-01	2.38E-01	7.09E-02	6.83E-01	3.12E-01	2.49E-01	7.84E-02	9.10E-01	3.41E-01	1.54E-01	9.73E-01	9.92E-01	2.81E-01	1.08E-01
[Sulfatide t41:1 -H]-	1.56E-16	1.96E-23	2.53E-02	7.70E-02	1.15E-05	2.66E-04	7.00E-01	9.89E-01	9.87E-01	2.29E-03	1.50E-01	7.89E-01	3.56E-02	1.86E-01	2.06E-03	2.99E-03	4.13E-04
[Sulfatide t42:0 -H]-	1.39E-17	6.69E-19	3.20E-01	3.87E-02	3.37E-01	1.09E-01	3.63E-01	7.62E-02	3.50E-01	1.29E-01	7.06E-01	4.93E-01	5.04E-01	6.48E-01	9.65E-01	3.93E-01	2.18E-01
[Sulfatide t42:1 -H]-	1.16E-14	4.92E-27	8.59E-03	4.78E-02	5.03E-02	8.72E-04	4.69E-01	9.96E-01	1.87E-01	5.27E-03	4.01E-01	6.04E-01	1.39E-02	3.38E-01	1.33E-01	5.29E-02	1.46E-03

One-way ANOVA and multiple testing correction. The table shows the p-values for Levene test to determine the homogeneity (H_0 = groups have equivalent variance) to choose the post hoc method (Tukey or Games-Howell); one-way ANOVA p-values for the seven pair-wise (p-value < 0.05 appears in green shadow); Tukey or Games-Howell P-values for the seven pair-wise. Abbreviations: ANOVA, analysis of variance; Hex Cer, Hexocyl Ceramide; LPI, Lyso phosphatidylinositol; PC, phosphatidylcholine; PC O/PC P; ether/vynil-ether phosphatydilcholine; PE, phosphatidylethanolamine; PE O/ PE P, ether/vynil-ether phosphatidylethanolamine; PG, phosphatydilglyerol; PI, phosphatidylinositol; PS, phosphatidylserine; PS O/ PS P, ether/vynil-ether phosphatidylserine; SM, sphingomyelin; SULF, Sulfatide; G, Glomeruli; PTI, Proximal tubule I; PTII, Proximal tubule II; CDT, cortical distal tubule; CMT, Corticomedular tubule; MTI, Medullary distal tubule I; MTII, Medullary distal tubule II; ITS, Interstitial structure.

Supplementary Table S2. Statistical analysis of the lipid families fingerprint of the discovery group

Lipid family	Leven test	ANOVA	GvsPTI	GvsPTII	GvsCDT	GvsCMT	GvsMTI	GvsMTII	GvsITS	PTIvsPTII	PTIvsCDT	PTIvsCMT	PTIvsMTI	PTIvsMTII	PTIvsITS	PTIIvsCDT
**Hex Cer**	1.76E-04	0.00E+00	5.83E-05	2.10E-04	1.33E-07	1.71E-07	1.54E-05	2.06E-09	9.37E-02	9.99E-01	2.78E-04	1.76E-04	4.34E-02	3.77E-11	2.57E-01	8.99E-04
**LPI**	1.01E-02	8.33E-12	7.41E-04	1.22E-01	6.23E-01	9.90E-01	8.17E-02	3.54E-01	7.22E-01	7.32E-01	4.37E-02	2.30E-02	2.36E-01	1.31E-05	2.89E-05	8.60E-01
**PC**	2.35E-02	9.43E-03	7.91E-01	8.17E-01	3.61E-01	9.61E-02	1.97E-01	9.92E-01	5.76E-03	1.00E+00	9.85E-01	6.96E-01	5.71E-01	5.74E-01	5.56E-01	9.93E-01
**PC O/ PC P**	5.46E-02	3.76E-21	6.47E-13	1.01E-07	3.62E-04	2.96E-01	7.14E-01	2.54E-09	1.00E+00	1.06E-03	9.24E-07	2.52E-09	7.47E-03	9.61E-01	6.48E-13	6.64E-01
**PE**	1.76E-02	2.74E-03	5.55E-03	6.82E-03	5.22E-02	1.14E-01	9.26E-02	9.95E-01	9.06E-02	1.00E+00	9.97E-01	9.98E-01	9.96E-01	1.62E-01	6.96E-01	1.00E+00
**PE O/ PE P**	7.02E-03	5.00E-19	4.73E-09	1.95E-04	1.07E-01	8.05E-01	1.00E+00	8.78E-08	1.00E+00	4.93E-03	1.75E-05	8.95E-07	5.47E-04	6.91E-01	4.16E-07	3.83E-01
**PG**	2.16E-16	1.32E-40	4.35E-01	8.59E-01	9.99E-01	3.53E-01	5.11E-02	7.90E-01	9.98E-01	9.42E-01	7.83E-01	6.58E-01	5.18E-02	9.79E-01	2.90E-01	9.99E-01
**PI**	5.18E-02	2.55E-08	1.39E-04	9.76E-01	4.46E-05	9.76E-02	7.56E-01	9.84E-01	9.98E-01	4.22E-03	1.00E+00	8.85E-01	9.93E-01	1.85E-04	3.26E-03	1.35E-03
**PS**	3.28E-04	4.96E-15	5.27E-03	3.60E-05	3.55E-05	2.23E-04	7.80E-01	1.40E-07	2.62E-01	1.94E-01	2.02E-01	6.24E-01	9.38E-02	1.47E-06	4.42E-01	9.89E-01
**PS O/ PS P**	4.92E-09	6.29E-13	3.94E-02	1.94E-02	3.04E-04	7.31E-05	4.30E-05	3.52E-05	4.60E-03	9.88E-01	1.94E-04	2.08E-05	1.07E-03	9.37E-08	6.14E-01	2.20E-01
**SM**	1.03E-03	5.28E-33	4.62E-06	3.93E-03	1.39E-10	6.26E-12	3.35E-11	4.05E-12	5.10E-04	6.40E-01	4.95E-08	7.96E-08	2.37E-11	9.82E-08	4.08E-01	1.30E-06
**SULF**	7.56E-11	6.33E-35	9.64E-01	9.28E-04	6.68E-06	6.20E-04	1.91E-02	1.79E-04	4.78E-03	1.64E-02	1.54E-05	8.80E-04	1.77E-02	1.92E-04	1.53E-02	4.02E-03

Lipid family	Leven test	ANOVA	PTIIvsCMT	PTIIvsMTI	PTIIvsMTII	PTIIvsITS	CDTvsCMT	CDTvsMTI	CDTvsMTII	CDTvsITS	CMTvsMTI	CMTvsMTII	CMTvsITS	MTIvsMTII	MTIvsITS	MTIIvsITS
**Hex Cer**	1.76E-04	0.00E+00	7.17E-04	2.12E-02	8.48E-09	5.30E-01	1.00E+00	5.17E-01	1.48E-05	4.61E-04	5.03E-01	1.32E-05	4.65E-04	7.21E-01	1.32E-03	1.42E-06
**LPI**	1.01E-02	8.33E-12	6.10E-01	1.24E-01	3.35E-03	8.33E-03	9.97E-01	1.01E-01	9.29E-03	4.29E-02	7.74E-02	2.65E-01	4.55E-01	7.41E-02	6.75E-02	1.00E+00
**PC**	2.35E-02	9.43E-03	7.73E-01	6.37E-01	5.96E-01	7.08E-01	9.95E-01	9.66E-01	2.56E-01	9.99E-01	1.00E+00	7.90E-02	1.00E+00	1.19E-01	9.94E-01	4.62E-02
**PC O/ PC P**	5.46E-02	3.76E-21	2.20E-02	7.45E-01	3.79E-01	5.90E-07	6.36E-01	9.96E-01	1.51E-02	1.18E-03	1.00E+00	1.65E-04	4.22E-01	1.09E-01	7.78E-01	1.01E-08
**PE**	1.76E-02	2.74E-03	1.00E+00	1.00E+00	2.48E-01	8.76E-01	1.00E+00	1.00E+00	4.65E-01	9.85E-01	1.00E+00	5.49E-01	9.91E-01	3.62E-01	9.28E-01	7.80E-01
**PE O/ PE P**	7.02E-03	5.00E-19	1.15E-02	1.20E-03	7.62E-02	1.24E-03	7.99E-01	9.90E-02	2.92E-04	1.91E-01	7.85E-01	1.15E-05	8.80E-01	1.91E-03	1.00E+00	3.44E-06
**PG**	2.16E-16	1.32E-40	4.65E-01	5.17E-02	1.00E+00	6.10E-01	4.06E-01	5.09E-02	9.93E-01	9.71E-01	3.44E-02	4.92E-01	3.23E-01	5.17E-02	5.07E-02	5.70E-01
**PI**	5.18E-02	2.55E-08	4.82E-01	9.56E-01	6.83E-01	1.00E+00	6.73E-01	9.66E-01	6.45E-05	1.07E-03	1.00E+00	3.63E-02	3.74E-01	4.63E-01	9.21E-01	8.45E-01
**PS**	3.28E-04	4.96E-15	9.79E-01	2.36E-02	3.55E-01	3.75E-03	1.00E+00	6.32E-02	1.91E-04	1.22E-03	4.64E-02	1.60E-02	2.14E-02	4.34E-02	1.90E-01	1.97E-07
**PS O/ PS P**	4.92E-09	6.29E-13	3.56E-02	1.47E-02	4.11E-03	9.94E-01	6.37E-01	2.84E-01	3.40E-03	7.09E-01	9.70E-01	8.92E-01	2.00E-01	1.00E+00	7.77E-02	3.16E-02
**SM**	1.03E-03	5.28E-33	9.60E-08	9.24E-09	5.57E-09	1.00E+00	4.72E-01	6.93E-06	4.23E-03	1.82E-09	3.96E-01	1.27E-01	1.83E-09	5.83E-01	1.38E-10	1.02E-08
**SULF**	7.56E-11	6.33E-35	7.40E-03	1.79E-02	3.58E-04	7.96E-01	6.17E-01	1.40E-02	1.61E-03	3.06E-01	1.37E-02	1.20E-02	4.83E-02	1.00E+00	8.81E-03	4.16E-04

One-way ANOVA and multiple testing correction. The table shows the p-values for Levene test to determine the homogeneity (H_0 = groups have equivalent variance) to choose the post hoc method (Tukey or Games-Howell); one-way ANOVA p-values; Tukey or Games-Howell P-values for the seven pair-wise (p-value < 0.05 appears in green shadow). Abbreviations: ANOVA, analysis of variance; Hex Cer, Hexocyl Ceramide; LPI, Lyso phosphatidylinositol; PC, phosphatidylcholine; PC O/ PC P; ether/vynil-ether phosphatydilcholine; PE, phosphatidylethanolamine; PE O/ PE P, ether/vynil-ether phosphatidylethanolamine; PG, phosphatydilglyerol; PI, phosphatidylinositol; PS, phosphatidylserine; PSO, ether/vynil-ether phosphatidylserine; SM, sphingomyelin; SULF, Sulfatide; G, Glomeruli; PTI, Proximal tubule I; PTII, Proximal tubule II; CDT, cortical distal tubule; CMT, Corticomedular tubule; MTI, Medullary distal tubule I; MTII, Medullary distal tubule II; ITS, Interstitial structure.

Supplementary Table S3. Acylic chains of the detected lipids species

Lipid specie	Acylic chains
HexCer d28:0	
HexCer d29:0	
HexCer d30:0	
HexCer d30:1	
HexCer d32:0	
HexCer d34:1	HexCer d18:1/16:0
HexCer d36:1	
HexCer d36:2	
HexCer d38:2	
HexCer t35:1	
HexCer t35:2	
LysoPI 16:0	
LysoPI 18:0	
LysoPI 18:1	
LysoPI 20:4	
PC 32:0	PC 16:0/16:0
PC 32:2	PC 16:1/16:1 PC 14:0/18:2
PC 33:2	PC 15:0/18:2
PC 34:1	PC 16:0/18:1
PC 34:4	PC 14:0/20:4
PC 35:1	PC 17:0/18:1
PC 35:2	
PC 36:1	PC 18:0/18:1
PC 36:2	PC 18:0/18:2
PC 36:5	PC 16:0/20:5
PC 36:6	PC 14:0/22:6
PC 38:4	PC 18:0/20:4
PC 38:5	PC 18:1/20:4
PC 38:6	PC 18:2/20:4
PC 38:7	PC 16:1/22:6
PC O-32:2	PC O-16:1/16:1 //PC P-16:0/16:1
PC O-34:2	PC O-16:1/18:1 // PC P-16:0/18:1
PC O-34:3	PC O-16:1/18:2 // PC P-16:0/18:2
PC O-34:5	
PC O-36:5	PC O-16:1/20:4 // PC P- 16:0/20:4
PC O-36:6	
PC O-36:7	
PC O-38:5	PC O-18:1/20:4 //PC P-18:0/20:4
PC O-38:6	PC O-16:0/22:6// PC P-18:1/20:4
PC O-38:7	
PE 34:0	PE 16:0/18:0
PE 34:1	PE 16:0/18:1
PE 34:2	PE 16:0/18:2
PE 35:2	PE 17:0/18:2
PE 36:1	PE 18:0/18:1
PE 36:2	PE 18:0/18:2
PE 36:3	PE 18:1/18:2
PE 36:4	PE 16:0/20:4
PE 37:1	
PE 37:2	
PE 38:1	
PE 38:2	PE 20:1/18:1
PE 38:4	PE 20:4/18:0
PE 38:5	PE 18:1/20:4
PE 38:6	PE 18:2/20:4
PE 40:4	PE 22:4/18:0
PE 40:5	PE 22:4/18:1
PE 40:6	PE 22:6/18:0
PE 40:7	PE 22:6/18:1

The assigment of the lipid species included in this work was validated by uHPLC.

Lipid specie	Acylic chains
PE O-34:2	PE O-16:1/18:1//PE P-16:0/18:1
PE O-34:3	PE O-16:1/18:2 //PE P-16:0/18:2
PE O-36:2	PE O-18:1/18:1 //PE P-18:0/18:1
PE O-36:3	PE O-16:1/20:4//PE-P 16:0/20:4
PE O-36:5	
PE O-38:5	PE O-18:1/20:4 //PE P-18:0/20:4
PE O-38:6	PE O-18:2/20:4 //PE P-18:1/20:4
PE O-38:7	PE O-16:1/22:6//PE P-16:0/20:4
PE O-40:5	
PE O-40:6	PE O-20:2/20:4// PE P-20:1/20:4
PE O-40:7	PE O-18:1/22:6// PE P 18:0/22:6
PG40:7	PG 18:1/22:6
PI 32:0	PI 16:0/16:0
PI 34:1	PI 16:0/18:1
PI 34:2	PI 16:1/18:1
PI 36:1	PI 18:0/18:1
PI 36:2	PI 18:0/18:0
PI 36:3	PI 18:1/18:2
PI 36:4	PI 16:0/20:4
PI 38:2	PI 20:1/18:1
PI 38:4	PI 18:0/20:4
PI 38:5	PI 16:0/22:5
PI 38:6	PI 16:1/22:5
PI 40:4	PI 20:0/20:4
PI 40:5	PI 18:0/22:5
PI 40:6	PI 18:0/22:6
PS 34:1	PS 16:0/18:1
PS 36:1	PS 18:1/18:0
PS 36:2	PS 18:1/18:1
PS 36:4	PS 18:2/18:2
PS 38:3	PS 18:0/20:3
PS 38:4	PS 18:0/20:4
PS 38:5	PS 18:2/20:3
PS 40:7	PS 20:4/20:3
PS O-38:2	
SM d32:1	SM d18:1/14:0
SM d33:1	
SM d34:1	SM d18:1/16:0
SM d34:2	
SM d35:1	
SM d36:1	SM d18:1/18:0
SM d36:2	SM d18:1/18:1
SM d38:1	SM d18:1/20:0
SM d38:2	
SM d40:1	SM d18:1/22:0
SM d40:2	
SM d42:1	SM d18:1/24:0
SM d42:2	SM d18:1/24:1
SM d42:3	SM d18:2/24:1
Sulfatide d34:1	Sulfatide d18:1/16:0
Sulfatide d34:2	
Sulfatide d34:3	
Sulfatide d36:3	
Sulfatide d36:4	
Sulfatide d38:1	
Sulfatide d40:1	
Sulfatide d40:2	
Sulfatide d41:1	
Sulfatide d41:2	
Sulfatide d42:1	
Sulfatide d42:2	
Sulfatide d42:3	
Sulfatide d43:3	
Sulfatide t41:0	
Sulfatide t41:1	
Sulfatide t42:0	
Sulfatide t42:1	

Supplementary Table S4. m/z of the detected lipid species

Lipid specie	Experimental m/z	Theoretical m/z
HexCer d28:0-H-	616.472	616.479
HexCer d29:0-H-	630.489	630.495
HexCer d30:0-H-	644.504	644.511
HexCer d30:1-H-	642.489	642.495
HexCer d32:0-H-	672.537	672.542
HexCer d34:1-H-	698.550	698.558
HexCer d36:1-H-	726.581	726.589
HexCer d36:2-H-	724.565	724.573
HexCer d38:2-H-	752.597	752.605
HexCer t33:1-H-	700.530	700.537
HexCer t35:1-H-	728.562	728.568
HexCer t35:2-H-	726.548	726.552
HexCer t39:4-H-	778.574	778.584
Lyso-PI 16:0-H-	571.291	571.289
Lyso-PI 18:0-H-	599.321	599.320
Lyso-PI 18:1-H-	597.303	597.305
Lyso-PI 20:4-H-	619.290	619.289
Lyso-PI-O 16:2/ Lyso-PI-P 16:1-H-	553.279	553.278
Lyso-PI-O 18:2/Lyso-PI-P 18:1-H-	581.310	581.310
PC 32:0-CH3-	718.540	718.539
PC 32:2-CH3-	714.510	714.508
PC 33:2-CH3-	728.528	728.524
PC 34:1-CH3-	744.555	744.555
PC 34:4-CH3-	738.502	738.508
PC 35:1-CH3-	758.571	758.571
PC 35:2-CH3-	756.557	756.555
PC 36:1-CH3-	772.584	772.586
PC 36:2-CH3-	770.576	770.571
PC 36:5-CH3-	764.523	764.524
PC 36:6-CH3-	762.510	762.508
PC 38:4-CH3-	794.571	794.571
PC 38:5-CH3-	792.556	792.555
PC 38:6-CH3-	790.542	790.539
PC 38:7-CH3-	788.522	788.524
PC-O 32:2/PC-P 32:1-CH3-	700.530	700.529
PC-O 34:2/PC-P 34:1-CH3-	728.562	728.560
PC-O 34:3/PC-P34:2-CH3-	726.548	726.544
PC-O 34:5/PC-P 34:4-CH3-	722.512	722.513
PC-O 36:5/PC-P 36:4-CH3-	750.542	750.544
PC-O 36:6/PC-P 36:5-CH3-	748.528	748.529
PC-O 36:7/PC-P 36:6-CH3-	746.513	746.513
PC-O 38:5/PC-P 38:4-CH3-	778.574	778.576
PC-O 38:6/PC-P 38:5-CH3-	776.565	776.560
PC-O 38:7/PC-P 38:6-CH3-	774.543	774.544
PE 34:0-H-	718.540	718.539
PE 34:1-H-	716.523	716.524
PE 34:2-H-	714.510	714.508
PE 35:2-H-	728.528	728.524
PE 36:1-H-	744.555	744.555
PE 36:2-H-	742.540	742.539
PE 36:3-H-	740.525	740.524
PE 36:4-H-	738.502	738.508
PE 37:1-H-	758.571	758.571
PE 37:2-H-	756.557	756.555
PE 38:1-H-	772.584	772.586
PE 38:2-H-	770.576	770.571
PE 38:4-H-	766.537	766.539
PE 38:5-H-	764.523	764.524
PE 38:6-H-	762.510	762.508
PE 40:4-H-	794.571	794.571
PE 40:5-H-	792.556	792.555
PE 40:6-H-	790.542	790.539
PE 40:7-H-	788.522	788.524

Lipid specie	Experimental m/z	Theoretical m/z
PE-O-34:2/PE-P 34:1-H-	700.530	700.529
PE-O-34:3/PE-P 34:2-H-	698.514	698.513
PE-O-36:2/PE-P 36:1-H-	728.562	728.560
PE-O-36:3/PE-P 36:2-H-	726.548	726.544
PE-O-36:5/PE-P 36:4-H-	722.512	722.513
PE-O-38:5/PE-P 38:4-H-	750.542	750.544
PE-O-38:6 /PE-P 38:5-H-	748.528	748.529
PE-O-38:7 /PE-P 38:6-H-	746.513	746.513
PE-O-40:5/PE-P 40:4-H-	778.574	778.576
PE-O-40:6/PE-P 40:5-H-	776.565	776.560
PE-O-40:7/PE-P 40:6-H-	774.543	774.544
PG 40:7-H-	819.517	819.518
PI 32:0-H-	809.520	809.519
PI 34:1-H-	835.533	835.534
PI 34:2-H-	833.519	833.519
PI 36:1-H-	863.566	863.566
PI 36:2-H-	861.550	861.550
PI 36:3-H-	859.537	859.534
PI 36:4-H-	857.515	857.519
PI 38:2-H-	889.585	889.581
PI 38:4-H-	885.547	885.550
PI 38:5-H-	883.534	883.534
PI 38:6-H-	881.522	881.519
PI 40:4-H-	913.580	913.581
PI 40:5-H-	911.563	911.566
PI 40:6-H-	909.550	909.550
PS 34:1-H-	760.513	760.513
PS 36:1-H-	788.545	788.545
PS 36:2-H-	786.527	786.529
PS 36:4-H-	782.498	782.498
PS 38:3-H-	812.543	812.545
PS 38:4-H-	810.527	810.529
PS 38:5-H-	808.516	808.513
PS 40:7-H-	832.510	832.513
PS-O 38:2/PS-P 38:1-H-	800.573	800.581
SM d32:1-CH3-	659.513	659.513
SM d33:1-CH3-	673.531	673.529
SM d34:1-CH3-	687.547	687.545
SM d34:2-CH3-	685.527	685.529
SM d35:1-CH3-	701.561	701.560
SM d36:1-CH3-	715.575	715.576
SM d36:2-CH3-	713.556	713.560
SM d38:1-CH3-	743.608	743.607
SM d38:2-CH3-	741.587	741.592
SM d40:1-CH3-	771.640	771.639
SM d40:2-CH3-	769.619	769.623
SM d42:1-CH3-	799.675	799.670
SM d42:2-CH3-	797.657	797.654
SM d42:3-CH3-	795.639	795.639
SM t32:2-CH3-	673.481	673.493
Sulfatide d34:1-H-	778.511	778.514
Sulfatide d34:2-H-	776.495	776.499
Sulfatide d34:3-H-	774.483	774.483
Sulfatide d36:3-H-	802.515	802.514
Sulfatide d36:4-H-	800.496	800.499
Sulfatide d38:1-H-	834.578	834.577
Sulfatide d40:1-H-	862.608	862.608
Sulfatide d40:2-H-	860.591	860.593
Sulfatide d41:1-H-	876.620	876.624
Sulfatide d41:2-H-	874.605	874.608
Sulfatide d42:1-H-	890.639	890.640
Sulfatide d42:2-H-	888.624	888.624
Sulfatide d42:3-H-	886.603	886.608
Sulfatide d43:3-H-	900.622	900.624
Sulfatide t35:0-H-	810.527	810.541
Sulfatide t41:0-H-	894.635	894.635
Sulfatide t41:1-H-	892.616	892.619
Sulfatide t42:0-H-	908.648	908.650
Sulfatide t42:1-H-	906.635	906.635
